# The role of COPD and inhaled corticosteroids in major adverse cardiovascular events in cardiovascular-kidney-metabolic populations

**DOI:** 10.1186/s12916-026-04754-7

**Published:** 2026-03-04

**Authors:** Anne E. Ioannides, Emily L. Graul, Constantinos Kallis, Upasana Tayal, Hana Müllerová, Jennifer K. Quint

**Affiliations:** 1 School of Public Health, Imperial College London, White City Campus, 90 Wood Lane, London, W12 0BZ UK; 2 Medical Scientist Training Program, Emory University School of Medicine, Atlanta, GA USA; 3 National Heart Lung Institute, Imperial College London, London, UK; 4 Royal Brompton & Harefield Hospitals, Guy’s and St Thomas’ NHS Foundation Trust, London, UK; 5 The George Institute for Global Health, London, UK; 6 Respiratory & Immunology BioPharmaceuticals Medical, AstraZeneca, Cambridge, UK

**Keywords:** COPD, MACE, Cardiovascular-kidney-metabolic syndrome, Cardiometabolic, ICS

## Abstract

**Background:**

Cardiovascular-kidney-metabolic (CKM) disease and chronic obstructive pulmonary disease (COPD) are associated with major adverse cardiovascular events (MACE). Whether COPD further increases MACE risk within CKM populations, and whether this potential risk is modifiable through inhaled corticosteroids (ICS), is unknown. Within CKM populations, we investigated the relationship between (1) COPD and subsequent MACE, and (2) amongst concurrent CKM-COPD populations, we investigated the relationship between ICS and subsequent MACE.

**Methods:**

We used Clinical Practice Research Datalink (CPRD) Aurum, Hospital Episode Statistics and Office of National Statistics data, between January 1st, 2010, and March 29th, 2021. We created five discrete cohorts: chronic kidney disease (CKD), type-II diabetes mellitus (T2DM), obesity, MACE history, and older adults (aged ≥ 65 years old [“Age65 + ”]). CKD, T2DM, obesity, and Age65 + cohorts were MACE-naïve at the time of inclusion. Aim (1) exposures were (a) COPD, (b) incident COPD, and (c) being at risk of COPD without diagnosis (defined as age ≥ 40 years old, smoking history, no evidence of asthma, and frequent respiratory infections requiring antibiotics). Aim (2) exposure was ICS prescription (control group: long-acting bronchodilators). The outcome was MACE (acute coronary syndrome, arrhythmia, heart failure, ischaemic stroke, or cardiovascular-specific mortality). We implemented Cox proportional hazards models.

**Results:**

COPD was associated with MACE amongst all cohorts, but was comparatively weak in the MACE history cohort (cohort total; adjusted hazard ratio [95% confidence interval]): CKD (*N* = 573,626; 1.29 [1.26, 1.32]), T2DM (*N* = 649,506; 1.30 [1.26, 1.35], obesity (*N* = 225,273; 1.41 [1.34, 1.48]), MACE history (*N* = 507,889; 1.04 [1.02, 1.06]), and Age65 + (*N* = 592,123, 1.59 [1.52, 1.66]). Incident COPD was associated with subsequent MACE in CKD only (1.28 [1.13, 1.45]). Being at risk of COPD was associated with subsequent MACE in CKD (1.18 [1.07, 1.30]), MACE history (1.16 [1.08, 1.25]), and Age65 + (1.28 [1.13, 1.46]). ICS prescription was not associated with subsequent MACE in any concurrent CKM-COPD cohort.

**Conclusions:**

COPD was an independent risk factor for MACE in CKM populations. ICS did not attenuate MACE amongst CKM-COPD groups. Incident COPD was associated with MACE in CKD, and being at risk of COPD was associated with MACE in CKD, MACE history, and Age65 + cohorts.

**Supplementary Information:**

The online version contains supplementary material available at 10.1186/s12916-026-04754-7.

## Background

Cardiovascular-kidney-metabolic (CKM) disease populations are at elevated risk of adverse disease-related outcomes and mortality due to multidirectional interactions between cardiovascular, metabolic, and renal diseases [1]. Major adverse cardiovascular events (MACE) frequently occur within people with CKM conditions, such as type-II diabetes mellitus (T2DM) [2, 3], chronic kidney disease (CKD) [4], and obesity [5]. Chronic obstructive pulmonary disease (COPD) also frequently exists in combination with other chronic age-related diseases, although often under-recognised and under-treated. However, COPD and exacerbations thereof are associated with increased risk of cardiovascular disease (CVD) of up to almost three times that of the general population, and CVD is often responsible for more deaths in COPD patients than COPD itself [6, 7]. Moreover, less well known is that COPD prevalence is also higher in CKM populations than the general population, including CKD [8] and T2DM [9, 10].

Despite recognition of elevated MACE risk in COPD patients, it is unknown whether the presence of COPD independently increases cardiovascular risk in populations already at elevated MACE risk (i.e. CKM populations), or whether the elevated MACE risk is due to other cardiovascular risk factors that tend to occur in people with COPD (e.g. hypertension or systemic inflammation).

COPD treatment has also classically been considered in isolation from management of other comorbidities, cardiovascular, or otherwise. However, evidence for the downstream effect of COPD treatment (specifically, inhaled corticosteroids [ICS]) on cardiovascular outcomes is mixed. Some post hoc trial data [11], observational research [12, 13], and a meta-analysis [14] suggest a cardioprotective effect, while other meta-analyses [15, 16] and observational research on a representative COPD population [17] suggest not. It is possible that the discrepancy in ICS-cardiovascular findings is related to population heterogeneity; ICS may be cardioprotective for COPD sub-populations, including, potentially, CKM populations with COPD.

What remains unknown is whether COPD is an independent cardiovascular risk factor in populations already at risk of MACE, and how the treatment of COPD may protect against MACE in these populations. By comparing MACE rates in people with an already elevated risk of MACE, we may be able to disentangle the role of COPD itself in cardiovascular burden in multimorbid patients.

### Overarching aim

We aimed to describe the relationship between COPD and subsequent MACE amongst CKM populations, and to understand the role of ICS therein.

### Specific objectives

1. Across study populations (CKD, T2DM, obesity, MACE history, and ≥ 65 years old), separately, we aimed to determine whether there was an association between three COPD-specific exposures ((a) pre-existing COPD, (b) incident COPD diagnosis, and (c) being at risk of COPD, but without diagnosis) and subsequent MACE.
2. Amongst people within study populations who had a COPD diagnosis (concurrent CKM-COPD), we aimed to determine whether there was an association between ICS prescriptions and subsequent MACE

## Methods

### Data source

We used routinely collected, de-identified, primary care records from the Clinical Practice Research Datalink (CPRD) Aurum database (December 2023 build [18]) linked with secondary care (Hospital Episode Statistics [HES] [19]), socioeconomic deprivation (Index of Multiple Deprivation [IMD] [20]), and mortality (Office of National Statistics [ONS] [21]) data. CPRD Aurum data are age-, sex-, and region-representative and cover approximately 20% of the UK population (majority England) [22].

### Cohorts

We created five discrete cohorts characterised by elevated MACE risk, namely (A) CKD, (B) T2DM, (C) obesity, (D) MACE history, and (E) people ≥ 65 years old (“Age65 + ”) (definitions in Additional file 1: Tab.E1). CKD, T2DM, obesity, and MACE history cohorts were to understand the role of COPD in MACE outcomes amongst CKM populations. Age (a known shared COPD-CVD independent risk factor) contextualised CKM population findings.

**Fig. 1:**
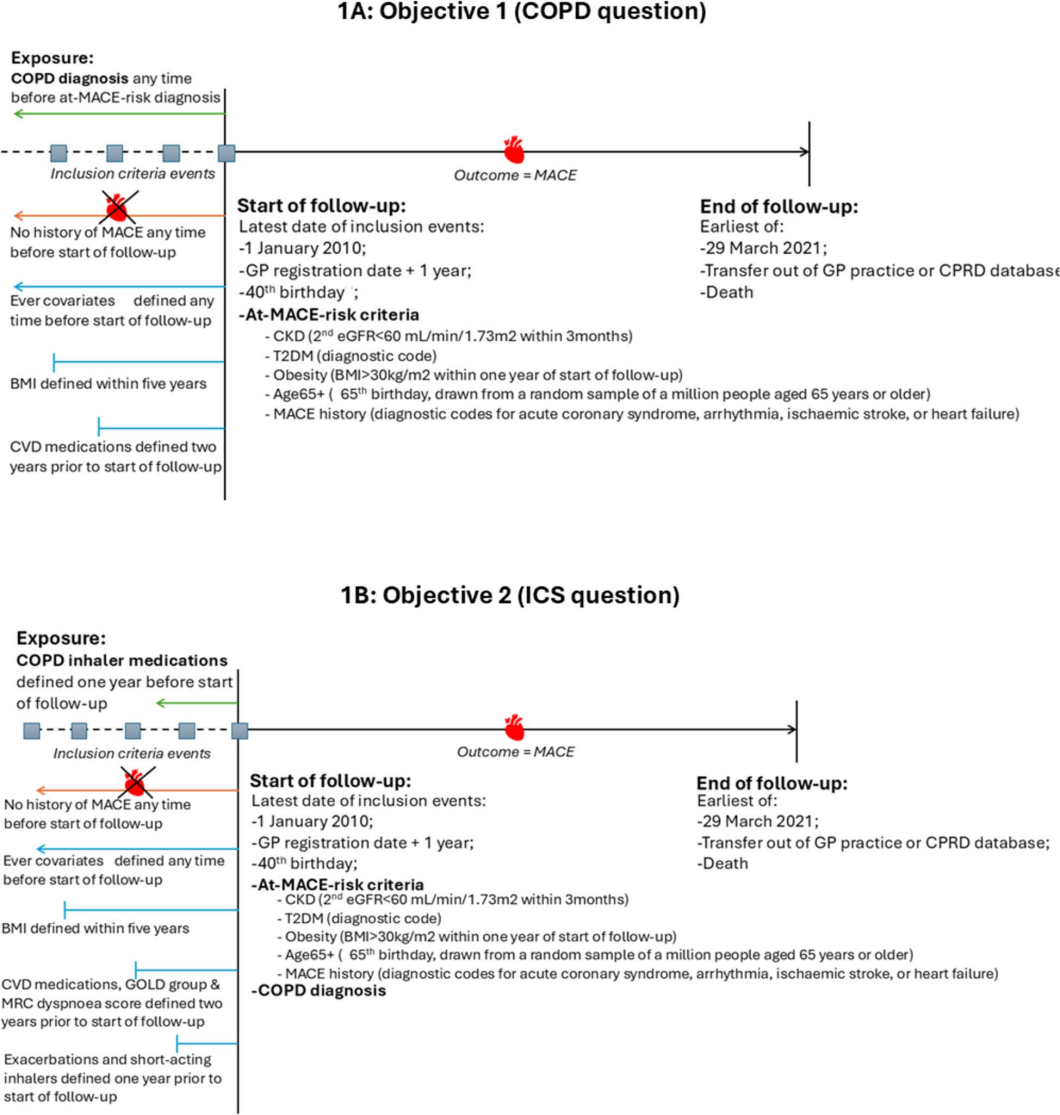
Study diagrams for **A** objective 1 (COPD question) and **B** objective 2 (ICS question). Demographic covariates derived from CPRD and linked data: age, sex, socioeconomic deprivation score (Index of Multiple Deprivation). Ever covariates: smoking status, hypertension, GORD, asthma, depression, anxiety, T2DM. No history of MACE any time before start of follow-up applies to CKD, T2DM, obesity, and Age65 + cohorts, but not to MACE history cohort. COPD inhaler medications include inhaled corticosteroids and long-acting bronchodilators (including long-acting beta agonists and long-acting muscarinic antagonists). Abbreviations: COPD, chronic obstructive pulmonary disease; MACE, major adverse cardiovascular event; BMI, body mass index; CVD, cardiovascular disease; GP, general practitioner (primary care); CKD, chronic kidney disease; T2DM, type-II diabetes mellitus; CPRD, Clinical Practice Research Datalink

People were included if they (1) were ≥ 40 years old (≥ 65 years old for Age65 + cohort); (2) had data recorded in CPRD Aurum between 1 st January 2010 and 29th March 2021; (3) registered at their GP practice for > 1 year before start of follow-up; (4) were acceptable for research (CPRD-determined); (5) met criteria for the characteristic that elevated MACE risk; and (6) were cardiovascular event-naïve at inclusion (except MACE history cohort) (Fig. 1A). For the second objective (ICS-specific question), people required a COPD diagnosis prior to the cohort-specific characteristic that elevated MACE risk, and ≥ one prescription of either ICS or a long-acting bronchodilator in the year preceding follow-up (Fig. 1B).

### Study design, exposures, and outcomes

#### Study design

We conducted five discrete retrospective cohort studies. Start of follow-up per cohort was the latest date of inclusion criteria (1)–(5) above. Follow-up ended at the earliest of the outcome, 29th March 2021, death, or GP out-transfer (Fig. 1) (individual diagrams in Additional file 1: Fig.E1–Fig.E5).

#### Exposure

For the COPD objective, there were three exposures, namely (a) COPD diagnosis, (b) incident COPD diagnosis, and (c) being at risk of COPD. COPD diagnosis was a primary care diagnostic code for COPD before first diagnosis of the cohort-specific condition (e.g. in the T2DM cohort, exposure constituted a COPD diagnosis before first T2DM diagnosis). COPD was defined from validated methodology [23] (codelist: https://github.com/NHLI-Respiratory-Epi/COPD-and-MACE-in-CRM-populations_codelists). Incident and at risk of COPD definitions are exploratory in nature, where incident COPD was a first COPD diagnosis within 6 weeks in either direction of the cohort-specific condition (CKD, T2DM, and MACE history cohorts only). The comparator group for COPD and incident COPD was people without COPD. Being at risk of COPD was defined as ≥ 40 years old, smoking history, and absence of asthma; as a sensitivity analysis, we used the aforementioned definition plus ≥ two respiratory infections requiring antibiotics within the preceding 2 years. The comparator group for being at risk of COPD was people without COPD and not at risk of COPD.

For the ICS objective, exposure was ≥ one prescription of ICS (monotherapy or combination therapy) in the year preceding follow-up. The comparator group was ≥ one prescription of a long-acting bronchodilator (monotherapy or combination therapy) without ICS.

#### Outcome

The outcome was MACE (acute coronary syndrome [ACS], arrhythmia, ischaemic stroke [stroke], heart failure [HF], or cardiovascular-specific death [CV-death]), defined in secondary care (codelists: https://github.com/NHLI-Respiratory-Epi/COPD-and-MACE-in-CRM-populations_codelists). MACE was incident for CKD, T2DM, obesity, and Age65 + cohorts, but was first MACE after inclusion for the MACE history cohort. As a secondary outcome, we analysed MACE-subtypes individually (e.g. ACS versus no ACS).

### Statistical analysis

#### Baseline characteristics and covariates

Baseline characteristics were taken as close as possible to start of follow-up (definitions in Additional file 1: Tab.E1; codelists: https://github.com/NHLI-Respiratory-Epi/COPD-and-MACE-in-CRM-populations_codelists). Characteristics were described as mean (standard deviation) for continuous data and as counts (percentage) for categorical data. Characteristics included age, sex, smoking status(never, current or ex), socioeconomic status (IMD quintile deprivation, IMD1 = least deprived to IMD5 = most deprived), hypertension, gastro-oesophageal reflux disease (GORD), asthma, depression, anxiety, CKD (except CKD cohort), cardiovascular medications, BMI category (except obesity cohort), and T2DM (except T2DM cohort). For secondary outcomes of individual MACE-subtypes, we described cardiovascular history (e.g. for ACS versus no ACS, we described history of stroke, arrhythmia, and HF).

For the ICS question, we also described acute exacerbations of COPD, MRC dyspnoea scale, Global Initiative for Chronic Obstructive Lung Disease (GOLD) airflow obstruction group, and short-acting bronchodilators.

#### Main analysis

Across cohorts, for both COPD and ICS questions, we calculated absolute events (numbers and percentages) for MACE and MACE-subtypes, and implemented covariate-adjusted Cox proportional hazard regressions to investigate associations with subsequent MACE.

#### Sensitivity analyses

We sequentially removed covariates with large amounts of missing-not-at-random data from models (COPD models: BMI and CKD; ICS models: CKD, BMI, GOLD group, and MRC dyspnoea group). We conducted sensitivity doubly robust propensity score-adjusted Cox proportional hazard regressions for ICS models to address confounding by indication [17].

As an experimental model that simulated coexistence of several comorbidities at once, we investigated the association between COPD and subsequent MACE amongst those at highest MACE risk by collapsing CKD, T2DM, obesity, and Age65 + cohorts into one cohort with a QRISK score > 10% by applying the QRISK algorithm to electronic healthcare records (peer-reviewed methodology [24], using publicly available algorithms [25]) (Additional file 1: Fig.E6).

Amongst models violating the proportional hazards assumption, we repeated adjusted models with time interaction, showing annual hazard ratios. We implemented Bonferroni correction (*P* < 0.0083′ threshold).

## Results

### Descriptive baseline characteristics

We built five discrete cohorts of people at elevated MACE risk: CKD (*N* = 573,626), T2DM (*N* = 649,506), obesity (*N* = 225,273), MACE history (507,889), and Age65 + (*N* = 592,123) (Table 1; inclusion flow diagrams in Additional file 1: Fig.E7–Fig.E11; baseline characteristics in Additional file 1: Tab.E2–Tab.E11).

**Table 1:** Numbers and percentages of people in exposed and control groups across cohorts

**Cohort**	**Whole cohort** *(N)*	**COPD** N (%)*	**No COPD** N (%)*	**Whole cohort eligible for ICS question**	**ICS** N (%)*	**LABA/LAMA** N (%)*
	*(Follow-up time)* *M (IQR) in years*	*(MACE outcome) N (%)*^*†*^	*(MACE outcome) N (%)*^*†*^	*(Follow-up time)* *M (IQR) in years*	*(MACE outcome) N (%)*^*†*^	*(MACE outcome) N (%)*^*†*^
**CKD**	573,626	41,628 (7.3)	531,998 (92.7)	24,168	18,360 (76.0)	5808 (24.0)
	(4.5 (2.0, 8.4))	(8390 (20.2))	(102,588 (19.3))	(3.1 (1.3, 5.7))	(3,918 (21.3))	(998 (17.2))
**T2DM**	649,506	29,358 (4.5)	620,148 (95.5)	17,595	13,448 (76.4)	4147 (23.6)
	(5.7 (2.5, 9.9))	(4497 (15.3))	(76,142 (12.3))	(3.6 (1.6, 6.4))	(2165 (16.1))	(519 (12.5))
**Obesity**	225,273	23,361 (10.4)	201,912 (89.6)	10,982	8345 (76.0)	2637 (24.0)
	(4.7 (2.2, 8.4))	(2566 (11.0))	(12,199 (6.0))	(3.4 (1.8, 5.6))	(936 (11.2))	(253 (9.6))
**MACE history**	507,889	54,562 (10.7)	453,327 (89.3)	34,114	25,661 (75.2)	8453 (24.8)
	(1.9 (0.5, 4.4))	(15,112 (27.7))	(126,419 (27.9))	(1.5 (0.4, 3.4))	(7054 (27.5))	(2166 (25.6))
**Age65 + **	592,123	22,818 (3.9)	569,305 (96.2)	13,066	10,173 (77.9)	2,893 (22.1)
	(5.9 (2.7, 9.9))	(2866 (12.7))	(71,569 (12.6))	(4.3 (1.7, 7.7))	(1431 (14.1))	(311 (10.8))
**QRISK > 10%**	1,267,660	72,682 (5.7)	1,194,978 (94.3)	NA	NA	NA
	(5.5 (2.4, 9.7))	(11,950 (16.4))	(184,898 (15.5))			

*Abbreviations*: *CKD *chronic kidney disease, *T2DM *type-II diabetes mellitus, *MACE *major adverse cardiovascular event, *ICS *inhaled corticosteroids, *LABA/LAMA *long-acting beta agonists or long-acting muscarinic antagonists

^*^Percentage of the whole cohort

^†^Percentage of the study group (i.e. within COPD or no COPD; or within inhaled corticosteroids or long-acting bronchodilators)

Baseline characteristics trends were similar across cohorts. Compared with people without pre-existing COPD, people with COPD had higher prevalence of current smokers and lower prevalence of ex-smokers, were slightly socioeconomically deprived, had higher prevalence of GORD and asthma, and slightly higher prevalence of anxiety, depression, and cardiovascular medications. Amongst obesity and MACE history cohorts, people with COPD had slightly higher prevalence of hypertension. Amongst the Age65 + cohort, people with COPD had a slightly higher prevalence of obesity, and a slightly lower prevalence of CKD (although CKD and BMI missingness were higher in people without COPD).

For the ICS question, compared with people on long-acting bronchodilators only, people on ICS had a higher prevalence of asthma, lower prevalence of short-acting bronchodilator prescriptions, slightly more COPD exacerbations, and were distributed slightly more heavily amongst more advanced disease severity groups.

### COPD and MACE

**Fig. 2:**
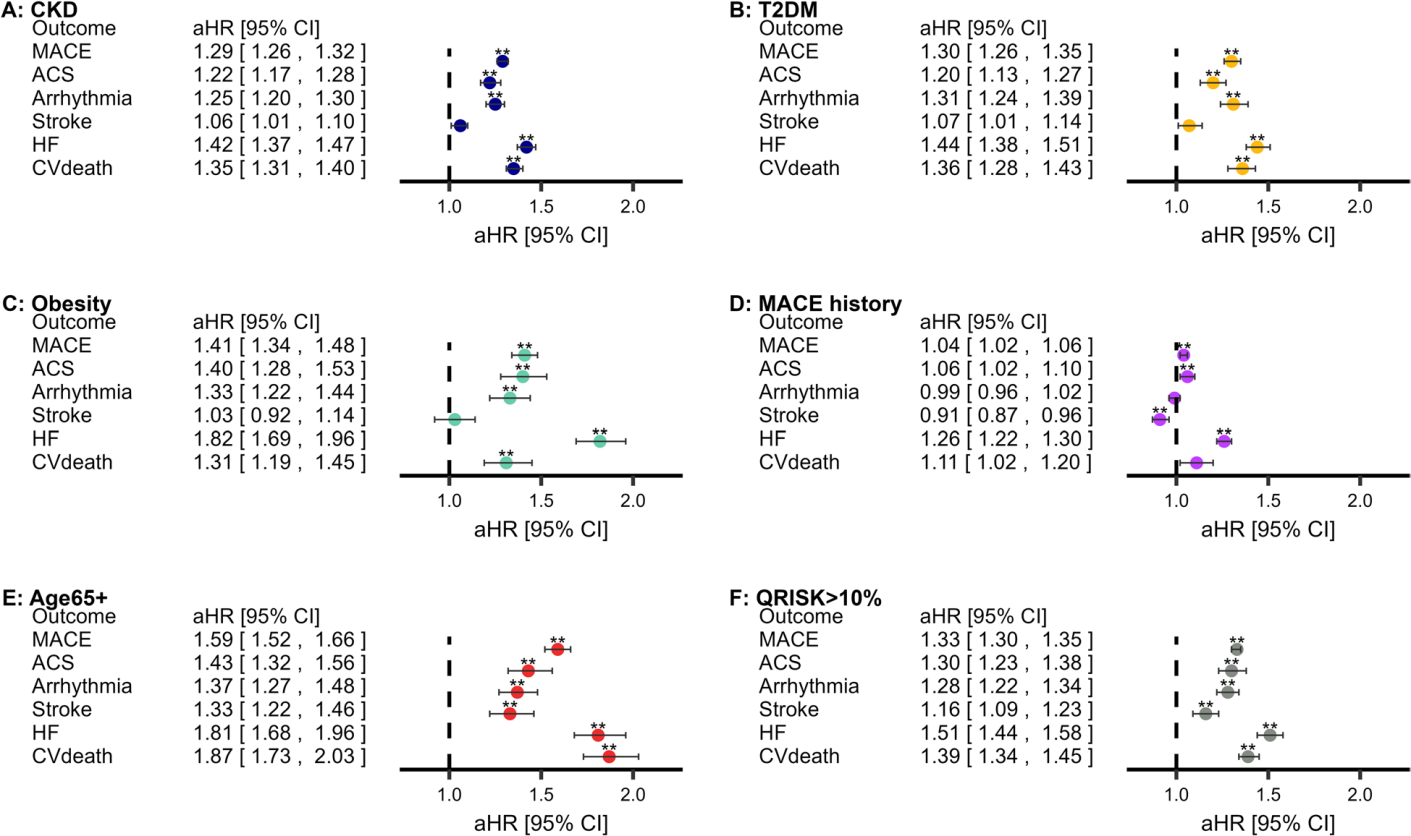
Association between pre-existing COPD and subsequent MACE amongst populations at elevated risk of MACE. **Statistically significant (*P* < 0.0083′ threshold). Adjusted for age, sex, socioeconomic deprivation, smoking status, hypertension, asthma, GORD, depression, anxiety, CVD medications, T2DM (except T2DM cohort), BMI (except obesity cohort), CKD (except CKD cohort), and cardiovascular history (MACE-subtypes only). Abbreviations: COPD, chronic obstructive pulmonary disease; MACE, major adverse cardiovascular event; BMI, body mass index; CKD, chronic kidney disease; T2DM, type-II diabetes mellitus; CVD, cardiovascular disease. Descriptive statistics, hazard ratios (95% confidence intervals), and sensitivity results are available in Additional file 1: Tab.E12

We assessed COPD and MACE in CKD, T2DM, obesity, MACE history, and Age65 + cohorts. COPD was associated with subsequent MACE across all cohorts (although weak in the MACE history cohort) (Fig. 2; Kaplan–Meier curves in Additional file 1: Fig.E12–Fig.E16; sensitivity analyses in Additional file 1: Tab.E12). Regarding MACE subtypes, COPD was associated with ACS, arrhythmia, HF, and CV-death within CKD, T2DM, obesity, and Age65 +. COPD was not associated with stroke amongst the aforementioned cohorts, except for Age65 +. MACE-subtype associations were mixed amongst the MACE history cohort: COPD was associated positively with ACS (weakly) and HF, negatively associated with ischaemic stroke, but not associated with CV-death (Fig. 2).

Amongst people with QRISK > 10% (descriptive characteristics in Additional file 1: Tab.E13), people with COPD had significantly higher MACE and MACE-subtype rates (Fig. 2F).

### Incident COPD and MACE

**Fig. 3:**
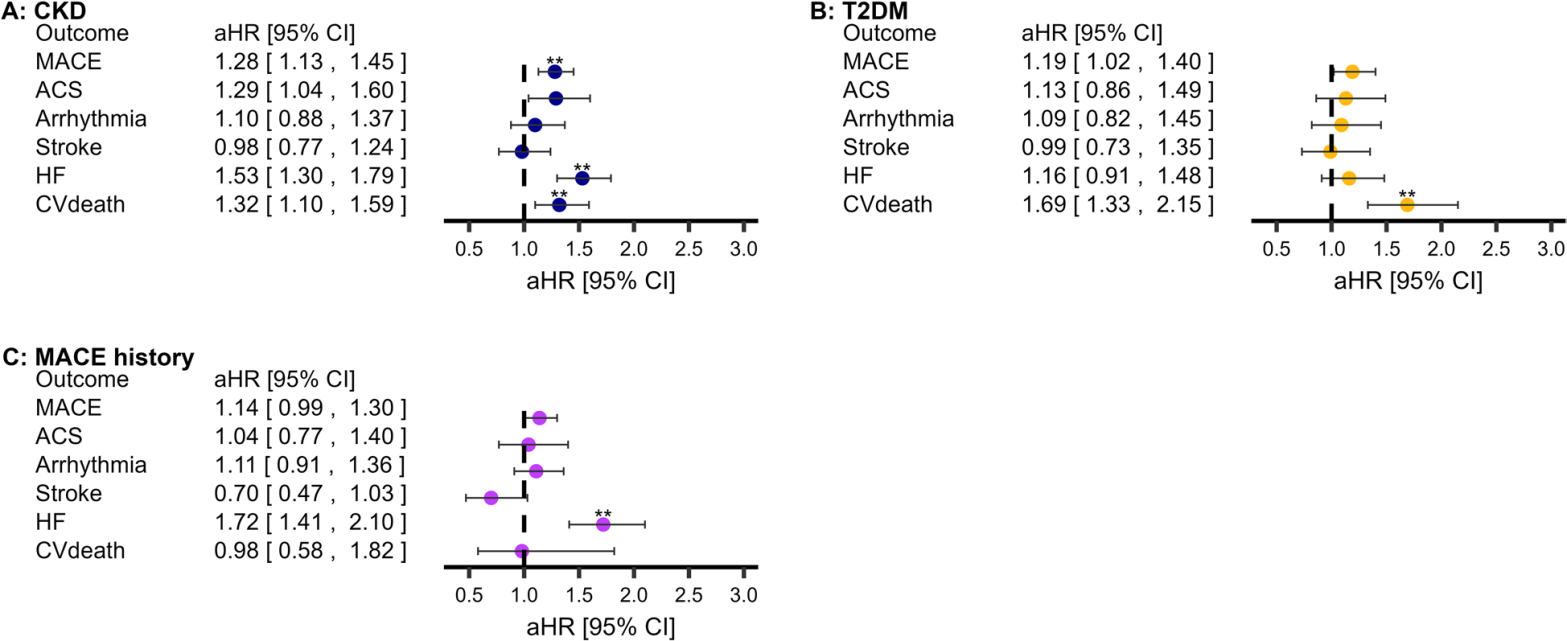
Association between incident COPD and subsequent MACE amongst populations at elevated risk of MACE. **Statistically significant (P < 0.0083′ threshold). Adjusted for age, sex, socioeconomic deprivation, smoking status, hypertension, asthma, GORD, depression, anxiety, CVD medications, T2DM (except T2DM cohort), BMI, CKD (except CKD cohort), and cardiovascular history (MACE-subtypes only). Abbreviations: COPD, chronic obstructive pulmonary disease; MACE, major adverse cardiovascular event; BMI, body mass index; CKD, chronic kidney disease; T2DM, type-II diabetes mellitus; CVD, cardiovascular disease. Descriptive statistics, hazard ratios (95% confidence intervals), and sensitivity results are available in Additional file 1: Tab.E14

We assessed incident COPD and MACE in T2DM, CKD, and MACE history cohorts. Incident COPD was associated with subsequent MACE in CKD, but not T2DM or MACE history cohorts (Fig. 3; sensitivity analyses in Additional file 1: Tab.E14). Regarding MACE-subtypes, incident COPD was associated with subsequent HF and CV-death in CKD, CV-death in T2DM, and HF in MACE history (Fig. 3; Additional file 1: Tab.E14).

### Elevated COPD risk and MACE

#### Elevated COPD risk without antibiotic-treated infections

**Fig. 4:**
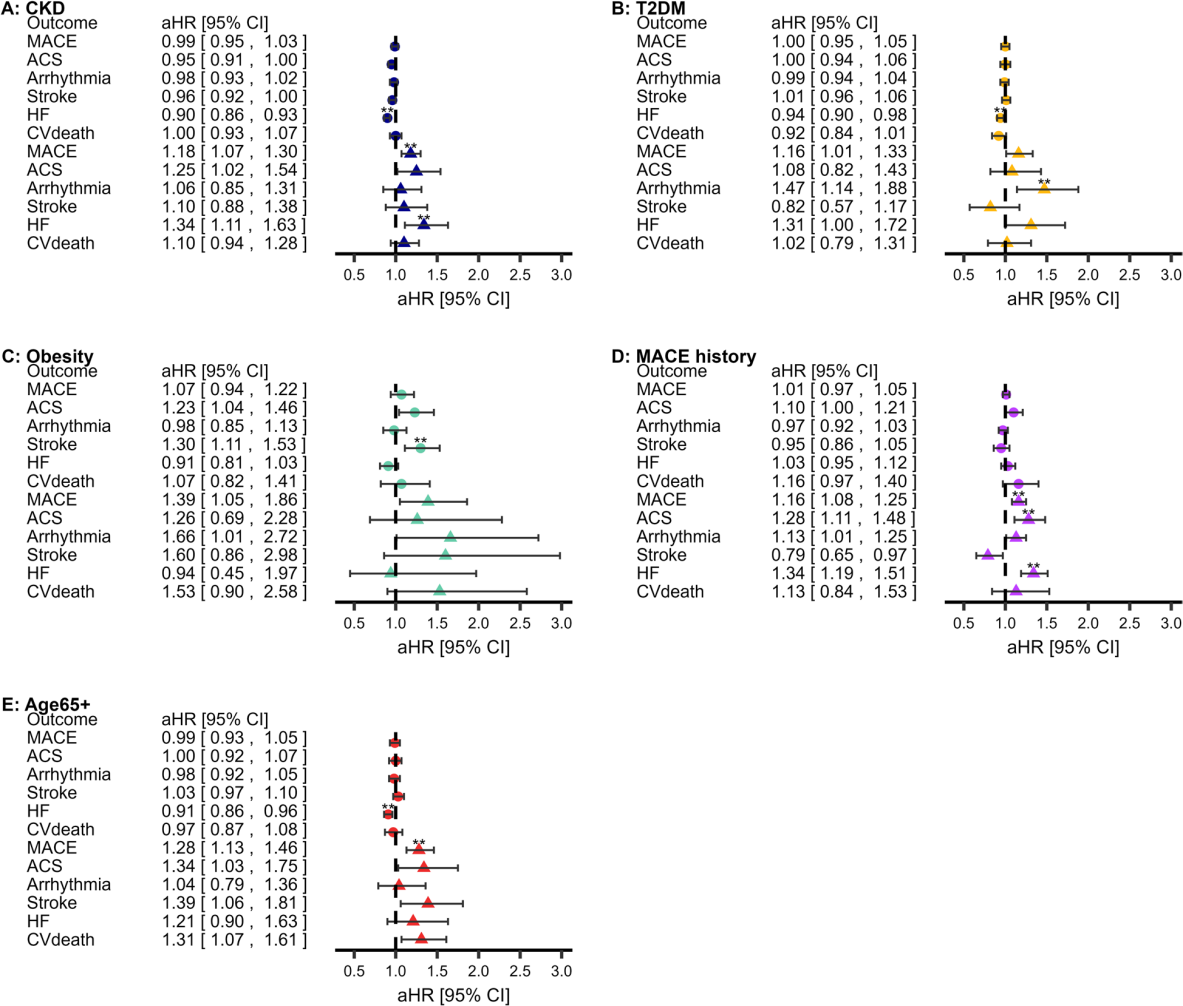
Association between being at risk of COPD and subsequent MACE amongst populations at elevated risk of MACE. O = without two-year frequent antibiotic-treated infection | Δ = with two-year frequent antibiotic-treated infection. **Statistically significant (*P* < 0.0083′ threshold). Adjusted for age, sex, socioeconomic deprivation, smoking status, hypertension, asthma, GORD, depression, anxiety, CVD medications, T2DM (except T2DM cohort), BMI, CKD (except CKD cohort), and cardiovascular history (MACE-subtypes only). Abbreviations: COPD, chronic obstructive pulmonary disease; MACE, major adverse cardiovascular event; BMI, body mass index; CKD, chronic kidney disease; T2DM, type-II diabetes mellitus; CVD, cardiovascular disease. Descriptive statistics, hazard ratios (95% confidence intervals), and sensitivity results are available in Additional file 1: Tab.E15 and Tab.E16

We assessed being at risk of COPD and subsequent MACE in CKD, T2DM, obesity, MACE history, and Age65 + cohorts. There was no association between being at risk of COPD without antibiotic-treated respiratory infections and subsequent MACE (Fig. 4; sensitivity analyses in Additional file 1: Tab.E15). Risk of COPD associations with MACE-subtypes were minimal, with only a positive association with subsequent stroke in people with obesity, and a negative association with subsequent HF in people with CKD, T2DM, and Age65 + (Fig. 4; sensitivity analyses in Additional file 1: Tab.E15).

#### Elevated COPD risk with antibiotic-treated infections

We assessed being at risk of COPD and subsequent MACE in CKD, T2DM, obesity, MACE history, and Age65 + cohorts. Being at risk of COPD with antibiotic-treated respiratory infections was associated with subsequent MACE in CKD, MACE history, and Age65 +, but not in T2DM or obesity cohorts (Fig. 4; sensitivity analyses in Additional file 1: Tab.E16). Amongst MACE-subtypes, COPD risk was associated with HF within CKD and MACE history, with arrhythmia within T2DM, and with ACS within MACE history (Fig. 4; sensitivity analyses in Additional file 1: Tab.E16).

### ICS and MACE

**Fig. 5:**
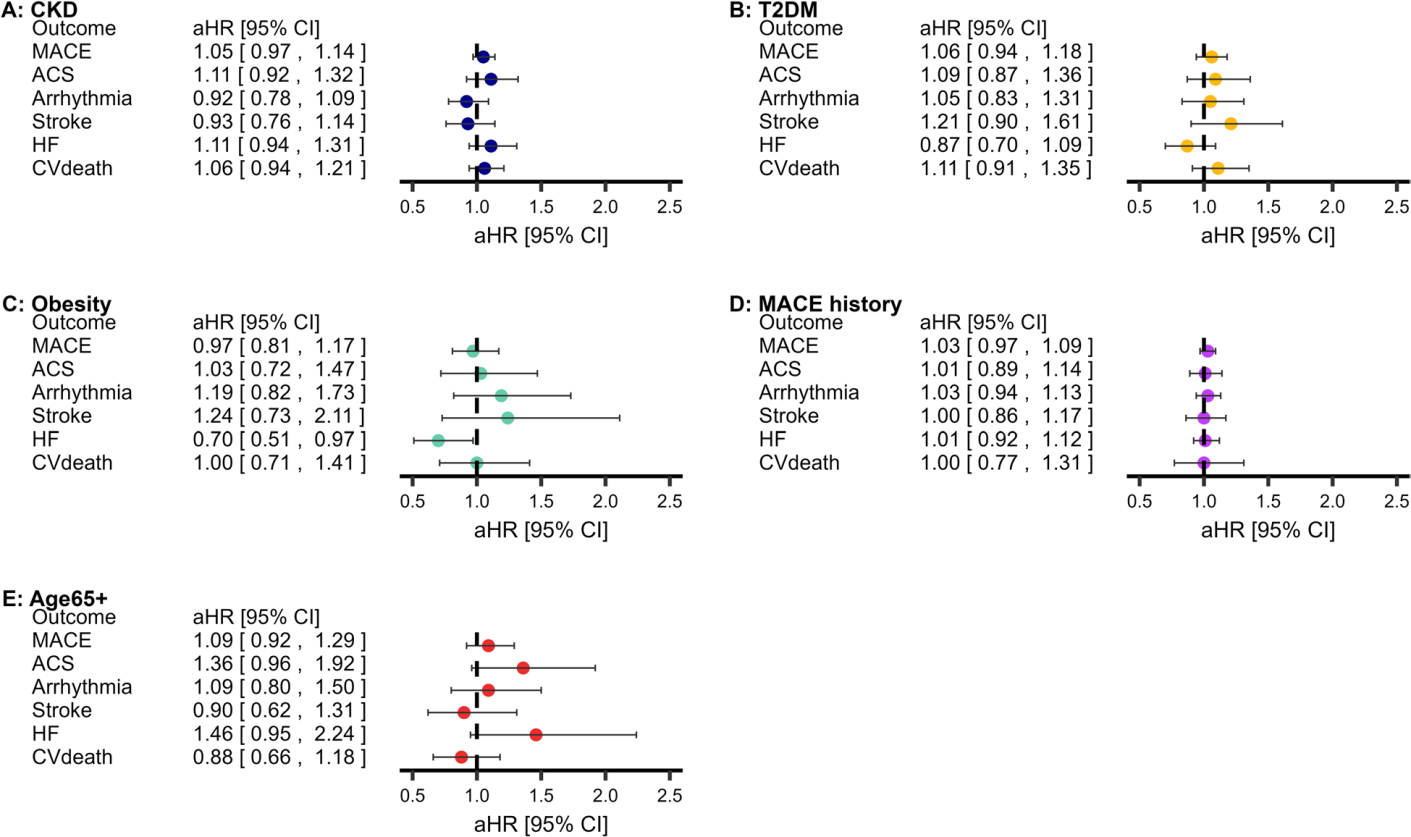
Relationship between ICS and subsequent MACE amongst populations at elevated risk of MACE. **Statistically significant (*P* < 0.0083′ threshold). Adjusted for age, sex, socioeconomic deprivation, smoking status, hypertension, asthma, GORD, depression, anxiety, CVD medications, T2DM, BMI, CKD, cardiovascular history (MACE-subtypes only), COPD exacerbations, MRC dyspnoea group, GOLD group, short-acting bronchodilators. Abbreviations: COPD, chronic obstructive pulmonary disease; MACE, major adverse cardiovascular event; BMI, body mass index; CKD, chronic kidney disease; T2DM, type-II diabetes mellitus; CVD, cardiovascular disease. Descriptive statistics, hazard ratios (95% confidence intervals), and sensitivity results are available in Additional file 1: Tab.E17

There was no association between ICS prescriptions and subsequent MACE amongst people with COPD in any cohort, compared with long-acting bronchodilators (Fig. 5; Kaplan–Meier curves in Additional file 1: Fig.E17–Fig.E21; sensitivity analyses in Additional file 1: Tab.E17).

### Sensitivity analysis and time interaction

Models that sequentially excluded covariates with missing data echoed results from main analyses. Time-interaction model annual hazard ratios (Additional file 1: Tab.E18–Tab.E27) did not change conclusions of any outcome.

## Discussion

We demonstrated, in CKM cohorts at elevated MACE risk, that pre-existing COPD was significantly associated with increased subsequent MACE, compared with people without COPD. COPD resulted in an increased MACE rate of between 29 and 41% amongst CKD, T2DM, obesity, and QRISK > 10% populations, while the increase was more modest in the MACE history population (4%). Amongst the Age65 + cohort (representing the known shared risk factor between COPD and CVD), there was a 59% increase in MACE rate compared with non-COPD controls. COPD was also associated with most MACE-subtypes across all cohorts, except for ischaemic stroke, where findings were mixed.

Findings were mixed for exploratory exposures of incident and risk of COPD. Incident COPD was associated with subsequent MACE only amongst people with CKD. Being at risk of COPD without antibiotic-treated respiratory infections was not associated with subsequent MACE, while being at risk of COPD with a history of antibiotic-treated respiratory infections was associated with subsequent MACE in CKD, Age65 +, and MACE history cohorts, but not in T2DM or obesity cohorts.

ICS prescriptions were not associated with subsequent MACE or MACE-subtypes, compared with long-acting bronchodilators without ICS.

### COPD and MACE amongst cohorts at elevated MACE risk: what this study adds

The association between COPD with subsequent MACE is well established when comparing COPD patients to the general population [26–28]. Previously unknown, however, was whether the inflated cardiovascular risk was attributable to COPD itself or to cardiovascular risk factors that typically occur in people with COPD (e.g. hypertension or systemic inflammation) that otherwise healthy individuals are less likely to have. By comparing people by COPD status amongst populations who are more balanced in cardiovascular risk factors due to having a condition that elevates their risk of a MACE (i.e. CKD, T2DM, obesity, MACE history, and older age), we determined that COPD is an independent risk factor for MACE. We highlighted that, in addition to the role of the cohort-specific conditions to cardiovascular risk, there is additional cardiovascular risk amongst patients with comorbid COPD. Current knowledge on COPD in relation to CKM populations relates largely to the association between the CKM condition and adverse outcomes related to COPD or the comorbidity in question, and not necessarily the cardiovascular element. In each case, we showed the additional layer of cardiovascular risk, even within cardiovascular event-naïve populations.

### Clinical implications

COPD is frequently under-recognised and under-treated, resulting in fragmented care resultant of single disease guidelines supported by trial populations [29]. However, we demonstrated, amongst UK-representative populations at elevated MACE risk, that pre-existing COPD elevates cardiovascular risk. That COPD is a risk factor for subsequent MACE in CKM populations demonstrates the need for earlier and more aggressive intervention for the COPD itself. The window for intervention appears to be more limited once the patient has had their first MACE, where the strength of the association between COPD and MACE is dramatically reduced.

### Contextualisation with literature

There is a known association between COPD and CKD [30], as well as between COPD and mortality (including respiratory-specific mortality) amongst people with CKD [31, 32]. There is also evidence to suggest a potential causal relationship between COPD and CKD [33]. However, to date, little is known about the cardiovascular consequences of COPD amongst people with CKD, even though factors associated with having COPD amongst people with CKD include cardiovascular risk factors (namely, T2DM, coronary artery disease, hypertension, and smoking) [32]. We demonstrated that COPD was associated with subsequent MACE in a CKD population. Moreover, we also showed that incident COPD (although we acknowledge that COPD’s insidious onset makes it difficult to ascertain whether the COPD is truly incident), as well as being at risk of COPD with antibiotic-treated respiratory infections, was associated with subsequent MACE. Furthermore, while the infection- and incident COPD-related findings are preliminary and hypothesis-generating, these findings do indicate that the role of COPD in CKD populations may be more important than previously recognised, particularly as COPD is not typically a first-line consideration in a CKD patient.

COPD rates are 35% higher amongst people with T2DM versus people without T2DM [34]. Much like CKD, risk factors for poorer lung function in people with T2DM (including metabolic syndrome, systemic inflammation, and insulin resistance) [35] are also important in the context of CVD. Yet, to our knowledge, ours is the first to investigate the role of COPD in the cardiovascular outcomes of T2DM patients.

The relationship between COPD and obesity is somewhat complex, with several studies suggesting that higher BMI may be protective against adverse COPD-related outcomes and mortality [36]. Equally, other work has shown an increased comorbidity burden, COPD exacerbation severity, and dyspnoea scores in people with obesity and COPD, compared with people who are of healthy weight or are overweight with COPD, despite marginally better lung function values [37]. Nevertheless, our work demonstrated that (despite a potential COPD-specific protective effect of obesity) cardiovascular risk amongst obesity patients is increased in the presence of COPD.

The association between COPD and MACE was weakest within the MACE history cohort. There is a well-established association between COPD and incident MACE [6]. While COPD is associated with a first MACE, we suspect that the association is somewhat attenuated after first MACE due to the “big event” having already occurred. Previous work using electronic healthcare records also demonstrated an association between COPD and MACE in a CVD population [38], although the magnitude of the association was more aligned with our at-risk but MACE-naïve cohorts. Although the overall message remains the same, the difference in magnitude may indicate that some CVD patient phenotypes are more at risk of subsequent MACE if they have COPD compared with other CVD patient phenotypes, thus, warranting further investigation. Hence, earlier and more aggressive screening for COPD and the shared risk factors between COPD and CVD in MACE-vulnerable populations is important to prevent the first MACE.

The Age65 + cohort contextualised our findings amongst CKM cohorts. The MACE rate difference between those with and without COPD was steepest in this cohort (59%). Characteristics of people with COPD are more like people without COPD 15 to 20 years their senior, and lung function decline accelerates with age [39]. Compared with age- and sex-matched controls, people with COPD had earlier and more frequent comorbidities as well as earlier mortality [39]. It is possible that the balance of unmeasured risk factors between people with and without COPD may be steeper in this cohort as they were not defined by a particular disease, but rather a random sample. Nevertheless, we have shown that older people are a patient group for whom effective management of COPD could offer large benefit to MACE outcomes. Secondly, as additional context, we addressed COPD amongst a collective cohort of people with a QRISK > 10% (i.e. highest MACE risk), findings of which aligned in strength and direction with CKM populations, which was unsurprising as CKM conditions are an important part of the QRISK algorithm, and the QRISK > 10% study cohort was an amalgamation of cardiovascular-naïve cohorts. COPD was recently added to the latest version of the QRISK algorithm, corroborating our results [40].

We investigated being at risk of COPD and subsequent MACE, with and without frequent antibiotic-treated infections. Being at risk of COPD without infection did not confer cardiovascular risk, while being at risk of COPD with antibiotic-treated respiratory infections history was associated with subsequent MACE amongst people with CKD, MACE history, and Age65 +. The role of respiratory infections is extremely important in COPD due to being the cause of COPD exacerbations. Likewise, there is a strong association (between three- to fivefold increase) between recent respiratory infections and subsequent MACE in the general population [41, 42]. While our numbers were small for this analysis, and while we acknowledge that these findings are exploratory, we speculate that infections may pose a downstream cardiovascular risk, but whether the effect is related to the consequential COPD exacerbations or the infections themselves is an unknown and important area of future research.

Amongst MACE-subtypes, COPD was associated with ACS, arrhythmia, heart failure, and CV-death, but not stroke, within cardiovascular-naïve CKM cohorts. There is mixed evidence regarding whether there is an association between COPD and stroke [43], including potential sex differences as a moderator of the association [44]. The absence of association between COPD events and subsequent stroke has been seen in other research using COPD patients from CPRD [27], and authors suggested the possibility that risk factors for stroke (such as arrhythmia) could have been picked up earlier and treated. Amongst the exploratory COPD exposures (incident and being at risk of COPD with infection), the MACE-subtype that emerged frequently as being associated with COPD was HF. However, some HF findings violated the proportional hazards assumption, and, furthermore, within people with COPD, it is difficult to disentangle whether these were true HF events, or misclassified COPD exacerbations, particularly in the context of infections. Nonetheless, we mitigated against misclassification as far as possible through validated methodology and codelists developed with both respiratory and cardiovascular clinicians. Moreover, COPD and HF are strongly associated [45], and, hence, management of COPD could mitigate the burden of HF.

### ICS in concurrent COPD and elevated MACE risk

Previous research suggested that there may be a cardioprotective role of ICS amongst people with COPD due to a potential reduction in systemic inflammation [14], while other research has said there is no direct cardiovascular benefit amongst COPD patients [17]. One suggestion for the discrepancy is population heterogeneity. Our work examined the role of ICS in COPD populations at elevated MACE risk, and we found no direct association in any CKM population, nor within the Age65 + contextualisation population. Although our findings on a general ICS versus long-acting bronchodilator comparison did not demonstrate a relationship between ICS and MACE, research on the indirect role of COPD management (including inhaler treatments, smoking cessation and pulmonary rehabilitation) on cardiovascular outcomes requires further investigation [46].

### Limitations and strengths

Our study had limitations. People without COPD prior to their cohort-specific characteristic may have been subsequently diagnosed with COPD. Although covariates were largely chronic and unlikely to change, we acknowledge that some may (e.g. BMI). Deciding which elements of MACE to include within the outcome was challenging due to heterogeneity in the literature relating to study-specific questions. We acknowledge a competing risk of all-cause mortality, but we selected cardiovascular-specific mortality to avoid non-CVD-related noise into findings. We also acknowledge potential misclassification of disease, particularly where symptoms overlap. Regarding the ICS question, although we implemented an intention-to-treat study design, some individuals may have changed medication type during follow-up.

Our work had several strengths. We investigated several conditions that increase MACE risk across different diseases, and our findings were consistent. Cohorts were from a database that is representative of the UK population, with a large degree of completeness of diagnostic coding. Methodologies used to identify cohorts, design codelists, and generate algorithms for covariates and exposures have been validated in previous studies [23, 27, 47–49]. We conducted several sensitivity analyses to confirm findings, and, for the ICS question, we conducted a sensitivity analysis adjusting models for propensity scores to address confounding by indication.

## Conclusions

Pre-existing COPD was independently associated with subsequent MACE amongst populations at elevated MACE risk. Preliminary evidence suggests that being at risk of COPD without a history of infections is not associated with MACE, while being at risk of COPD with infections requiring antibiotics is associated with MACE amongst some populations at elevated MACE risk. Amongst people with COPD and an elevated MACE risk, prior ICS prescriptions were not associated with decreased MACE risk.

## Supplementary Information

Additional file 1: supplementary information about data quality and CPRD processes, as well as cohort study design schematic diagrams, variable definitions and algorithms, cohort number flow diagrams, individual descriptive statistics per cohort, Kaplan–Meier curves, and sensitivity analysis results

## Data Availability

Datasets generated and/or analysed in this study are not publicly available, however, data are available on request from the CPRD. Their provision requires the purchase of a license, and this license does not permit the authors to make them publicly available to all. This work used data from the version collected in December 2023 and has clearly specified the data selected in the Methods section. To allow identical data to be obtained by others, via the purchase of a license, the codelists will be provided upon request. Licenses are available from the CPRD (http://www.cprd.com): The Clinical Practice Research Datalink Group, The Medicines and Healthcare products Regulatory Agency, 10 South Colonnade, Canary Wharf, London E14 4PU.

## Abbreviations

ACS: Acute coronary syndrome
BMI: Body mass index
CKD: Chronic kidney disease
COPD: Chronic obstructive pulmonary disease
CPRD: Clinical Practice Research Datalink
CKM: Cardiovascular-kidney-metabolic
CVD: Cardiovascular disease
CV-death: Cardiovascular-specific death
GOLD: Global Initiative for Chronic Obstructive Lung Disease (disease severity group)
GORD: Gastro-oesophageal reflux disease
HES: Hospital Episode Statistics
HF: Heart failure
ICS: Inhaled corticosteroids
IMD: Index of Multiple Deprivation
MACE: Major adverse cardiovascular event
MRC [dyspnoea score]: Medical Research Council [dyspnoea score]
ONS: Office of National Statistics
QRISK: No specific acronym; algorithm to estimate individual’s 10-year risk of myocardial infarction or ischaemic stroke
Stroke: Ischaemic stroke only
T2DM: Type-II diabetes mellitus
UK: United Kingdom
