# Supplementary material for: The role of COPD and inhaled corticosteroids in major adverse cardiovascular events in cardiovascular-kidney-metabolic populations
Source: BMC Med. 2026 Mar 4;24:221. doi: 10.1186/s12916-026-04754-7 (PMC13067444; doi:10.1186/s12916-026-04754-7)
Supplement: Supplementary file 1 — Additional file 1: supplementary information about data quality and CPRD processes, as well as cohort study design schematic diagrams, variable definitions and algorithms, cohort number flow diagrams, individual descriptive statistics per cohort, Kaplan–Meier curves, and sensitivity analysis results [file 12916_2026_4754_MOESM1_ESM.pdf]

COPD, INHALED CORTICOSTEROIDS AND MAJOR CARDIOVASCULAR EVENTS IN CARDIOVASCULAR-KIDNEY-METABOLIC COHORTS

Anne E. Ioannides PhD[1]*, Emily L. Graul MSc[1,2], Constantinos Kallis PhD[1], Upasana Tayal FRCP[3,4,5], Hana Müllerová PhD[6] and Jennifer K. Quint FRCP[1]

[1] School of Public Health, Imperial College London, London, UK

[2] Medical Scientist Training Program, Emory University School of Medicine, Atlanta, Georgia, USA

[3] National Heart Lung Institute, Imperial College London, UK

[4] Royal Brompton & Harefield Hospitals, Guy's and St Thomas' NHS Foundation Trust, London, UK

[5] The George Institute for Global Health, UK

[6] Respiratory & Immunology BioPharmaceuticals Medical, AstraZeneca, Cambridge, UK

***Correspondence to A.E. Ioannides:** a.ioannides@imperial.ac.uk; School of Public Health (SPH), Imperial College London – White City Campus, 86 Wood Lane, London, W12 0BZ, United Kingdom

ADDITIONAL FILE 1

Supplementary information about data quality and CPRD processes, as well as cohort study design schematic diagrams, variable definitions and algorithms, cohort number flow diagrams, individual descriptive statistics per cohort, Kaplan-Meier curves, and sensitivity analysis results.

Index

ITEM	PAGE NUMBER
**CPRD: additional linkage, data availing, consent for publication and ethics statements, and research data quality information**	**3**
Ethics approval and consent to participate	3
Consent for publication, and availability of data and materials	3
Additional linkage information	3
Data research quality information	3
**Study diagrams**	**4-9**
**Figure E1:** Study diagrams for the CKD cohort for (e1.1) COPD-specific, and (e1.2) ICS-specific questions	4
**Figure E2:** Study diagrams for the T2DM cohort for (e2.1) COPD-specific, and (e2.2) ICS-specific questions	5
**Figure E3:** Study diagrams for the Obesity cohort for (e3.1) COPD-specific, and (e3.2) ICS-specific questions	6
**Figure E4:** Study diagrams for the MACE history cohort for (e4.1) COPD-specific, and (e4.2) ICS-specific questions	7
**Figure E5:** Study diagrams for the Age65+ cohort for (e5.1) COPD-specific, and (e5.2) ICS-specific questions	8
**Figure E6** Study diagram for QRISK>10% cohort for COPD-specific study questions	9
**Variables definitions list**	10-13
**Table E1:** Definitions and dataset locations for cohorts, exposures, outcomes, and covariates	10-13
**Study flow chart and baseline characteristics per cohort**	**14-38**
**Figure E7:** Inclusion and exclusion flow diagram for the CKD cohort	14
**Table E2**: COPD-specific baseline characteristics for chronic kidney disease (CKD) population	15-16
**Table E3:** ICS-specific baseline characteristics for CKD population with COPD	17-18
**Figure E8:** Inclusion and exclusion flow diagram for the T2DM cohort	19
**Table E4:** COPD-specific baseline characteristics for T2DM population	20-21
**Table E5:** ICS-specific baseline characteristics for T2DM population with COPD	22-23
**Figure E9** Inclusion and exclusion flow diagram for the Obesity cohort	24
**Table E6:** COPD-specific baseline characteristics for Obesity (BMI>30kg/m^2) population	25-26
**Table E7:** ICS-specific baseline characteristics for Obesity (BMI>30kg/m^2) population with COPD	27-28
**Figure E10** Inclusion and exclusion flow diagram for the MACE history cohort	29
**Table E8:** COPD-specific baseline characteristics for MACE history population	30-31
**Table E9:** ICS-specific baseline characteristics for MACE history population with COPD	32-33
**Figure E11:** Inclusion and exclusion flow diagram for the Age65+ cohort	34
**Table E10:** COPD-specific baseline characteristics for Age65+ population	35-36
**Table E11:** ICS-specific baseline characteristics for Age65+ population with COPD	37-38
**Kaplan Meier (KM) Plots: COPD exposure**	**39-41**
**Figure E12:** KM plot for pre-existing COPD versus no COPD analysis for CKD cohort	39
**Figure E13:** KM plot for pre-existing COPD versus no COPD analysis for T2DM cohort	39
**Figure E14:** KM plot for pre-existing COPD versus no COPD analysis for Obesity cohort	40
**Figure E15:** KM plot for pre-existing COPD versus no COPD analysis for MACE history cohort	40
**Figure E16:** KM plot for pre-existing COPD versus no COPD analysis for Age65+ cohort	41
**Sensitivity analysis results: COPD exposures**	**42-49**
**Table E12**: Relationship between pre-existing COPD and subsequent MACE amongst six populations at elevated risk of MACE	42-43
**Table E13:** COPD-specific baseline characteristics for QRISK>10% population	44

**Table E14**: Relationship between incident COPD and subsequent MACE amongst five populations at elevated risk of MACE	45
**Table E15**: Relationship between being at risk of COPD in the absence of history of frequent antibiotic-treated lower respiratory tract infections and subsequent MACE amongst five populations at elevated risk of MACE	46-47
**Table E16**: Relationship between being at risk of in the presence of history of frequent antibiotic-treated lower respiratory tract infections and subsequent MACE amongst five populations at elevated risk of MACE	48-49
**Kaplan Meier (KM) Plots: ICS exposure**	**50-52**
**Figure E17:** KM plot for pre-existing ICS versus no ICS analysis for CKD cohort	50
**Figure E18:** KM plot for pre-existing ICS versus no ICS analysis for T2DM cohort	50
**Figure E19:** KM plot for pre-existing ICS versus no ICS analysis for Obesity cohort	51
**Figure E20:** KM plot for pre-existing ICS versus no ICS analysis for MACE history cohort	51
**Figure E21:** KM plot for pre-existing ICS versus no ICS analysis for Age65+ cohort	52
**Sensitivity analysis results: ICS exposure**	**53-55**
**Table E17**: Relationship between ICS and subsequent MACE amongst five populations at elevated risk of MACE	53-55
**Time interaction models (annual hazard ratios)**	**56-59**
**Table E18:** Yearly rate of HF among people with CKD during follow-up, amongst people who are at risk of COPD without infections compared with people with no COPD and without being at risk of COPD, where the Proportional Hazards Assumption was violated	56
**Table E19:** Yearly rate of HF among people with CKD during follow-up, amongst people who are at risk of COPD with infections compared with people with no COPD and without being at risk of COPD, where the Proportional Hazards Assumption was violated	56
**Table E20:** Yearly rate of MACE among people with T2DM during follow-up, amongst people with COPD pre-diagnosis compared with no COPD, where the Proportional Hazards Assumption was violated	57
**Table E21:** Yearly rate of heart failure among people with T2DM during follow-up, amongst people with COPD pre-diagnosis compared with no COPD, where the Proportional Hazards Assumption was violated	57
**Table E22:** Yearly rate of MACE among people with MACE history during follow-up, amongst people with COPD pre-diagnosis compared with no COPD, where the Proportional Hazards Assumption was violated	58
**Table E23:** Yearly rate of ACS among people with MACE history during follow-up, amongst people with COPD pre-diagnosis compared with no COPD, where the Proportional Hazards Assumption was violated	58
**Table E24:** Yearly rate of HF among people with MACE history during follow-up, amongst people with COPD pre-diagnosis compared with no COPD, where the Proportional Hazards Assumption was violated	58
**Table E25:** Yearly rate of arrhythmias among people Age65+ during follow-up, amongst people with COPD pre-diagnosis compared with no COPD, where the Proportional Hazards Assumption was violated	59
**Table E26:** Yearly rate of MACE among people with QRISK>10% during follow-up, amongst people with COPD pre-diagnosis compared with no COPD, where the Proportional Hazards Assumption was violated	59
**Table E27:** Yearly rate of MACE among people with HF history during follow-up, amongst people with COPD pre-diagnosis compared with no COPD, where the Proportional Hazards Assumption was violated	59

CPRD: additional linkage, data availing, consent for publication and ethics statements, and research data quality information

Ethics approval and consent to participate

CPRD has NHS Health Research Authority (HRA) Research Ethics Committee (REC) approval to allow the collection and release of anonymised primary care data for observational research [NHS HRA REC reference number: 05/MRE04/87]. Each year CPRD obtains Section 251 regulatory support through the HRA Confidentiality Advisory Group (CAG), to enable patient identifiers, without accompanying clinical data, to flow from CPRD contributing GP practices in England to NHS Digital, for the purposes of data linkage [CAG reference number: 21/CAG/0008]. The protocol for this research was approved by CPRD's Research Data Governance (RDG) Process (protocol number: 22_002514) and the approved protocol is available upon request. Linked pseudonymised data was provided for this study by CPRD. Data is linked by NHS Digital, the statutory trusted third party for linking data, using identifiable data held only by NHS Digital. Select general practices consent to this process at a practice level with individual patients having the right to opt-out.

Consent for publication, and availability of data and materials

Datasets generated and/or analysed in this study are not publicly available, however, data are available on request from the CPRD. Their provision requires the purchase of a license, and this license does not permit the authors to make them publicly available to all. This work used data from the version collected in December 2023 and has clearly specified the data selected in the Methods section. To allow identical data to be obtained by others, via the purchase of a license, the code lists will be provided upon request. Licenses are available from the CPRD (*http://www.cprd.com*): The Clinical Practice Research Datalink Group, The Medicines and Healthcare products Regulatory Agency, 10 South Colonnade, Canary Wharf, London E14 4PU.

Additional linkage information

Linked pseudonymised socioeconomic data from the Index of Multiple Deprivation (IMD), and secondary care data from Hospital Episode Statistics (HES) were provided for this study by CPRD for patients in England. Data is linked by NHS Digital, the statutory trusted third party for linking data, using identifiable data held only by NHS Digital. Select general practices consent to this process at a practice level, with individual patients having the right to opt-out. Use of HES and ONS data is Copyright © (2023), re-used with the permission of The Health & Social Care Information Centre

Data research quality information

CPRD checks several elements of data quality prior to making these data available for research (such as the volume of the data, data elements, and checks at different levels). Details on the data quality and the strategies undertaken by CPRD to ensure data quality are available at *https://www.cprd.com/data-quality*. Moreover, Wolf et. al. (2019, doi: *https://doi.org/10.1093/ije/dyz034*) offers a data profile on CPRD data, including demographic information and population representation, linkage to other datasets, and data strengths and weaknesses.

STUDY DIAGRAMS

Chronic Kidney Disease (CKD)

Figure E1: Study diagrams for the CKD cohort for (S1.1) COPD-specific, and (S1.2) ICS-specific questions

e1.1 COPD-specific study questions

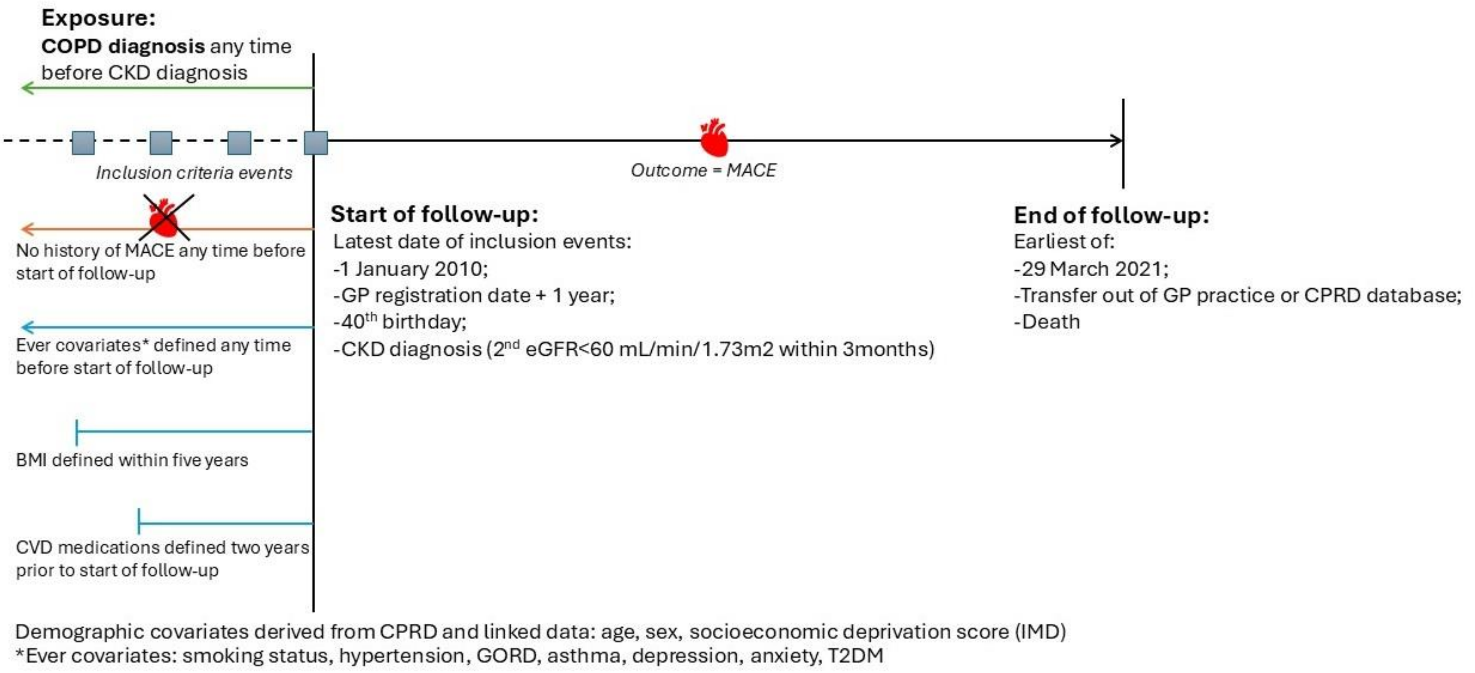

e1.2 ICS-specific study question

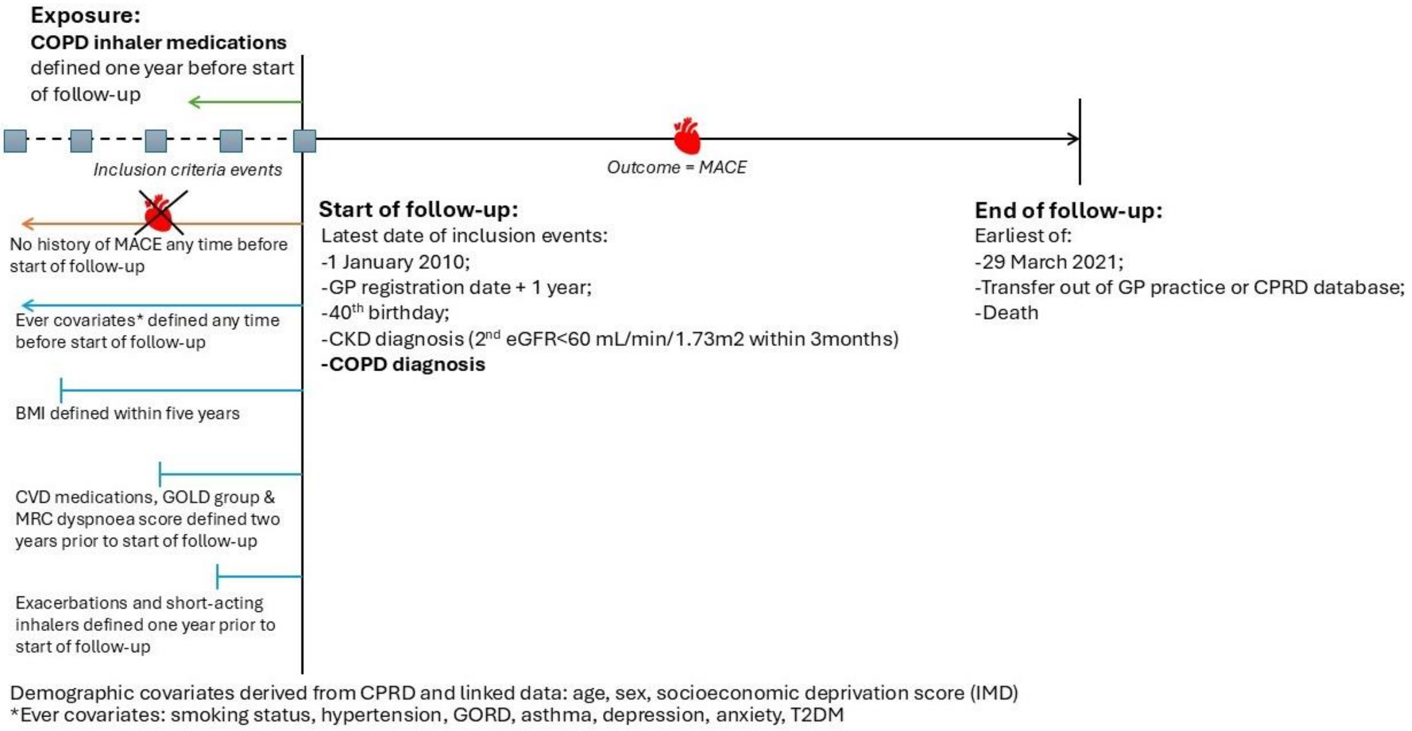

Type-II Diabetes Mellitus (T2DM)

Figure E2: Study diagrams for the T2DM cohort for (S2.1) COPD-specific, and (S2.2) ICS-specific questions

e2.1 COPD-specific study questions

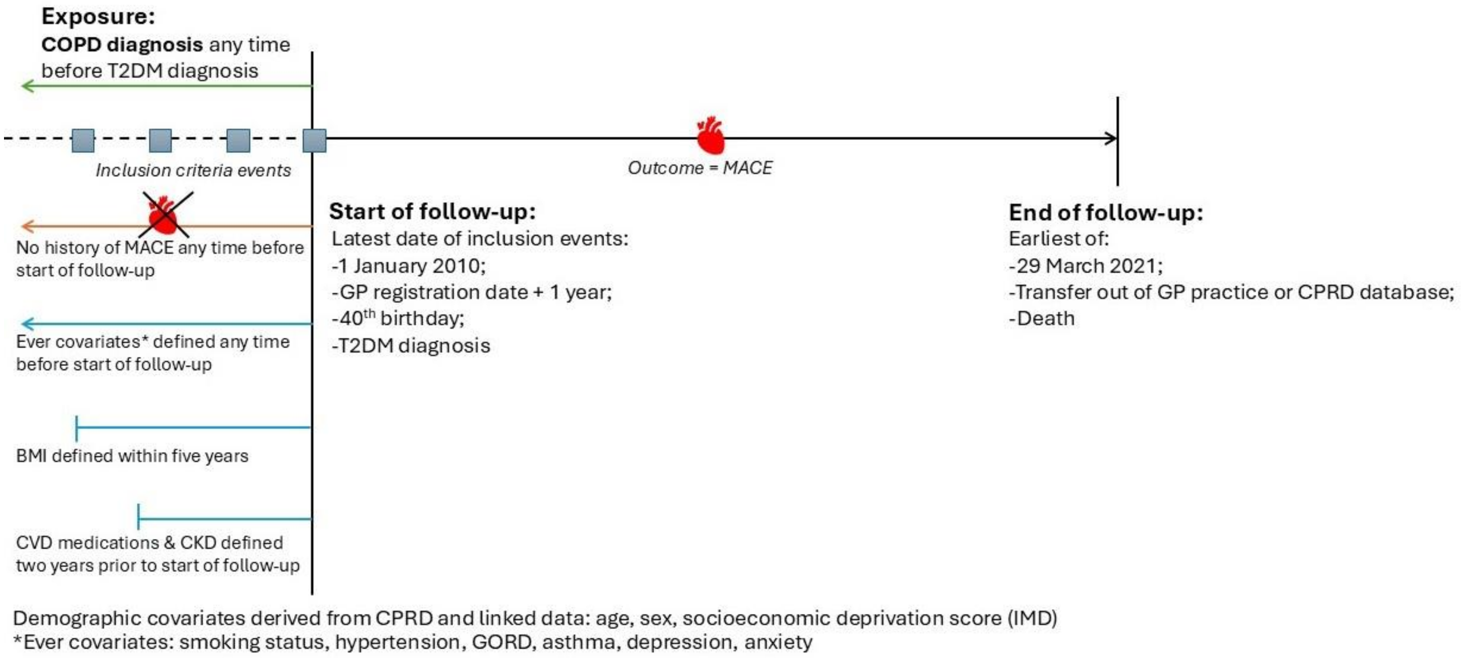

e2.2 ICS-specific study question

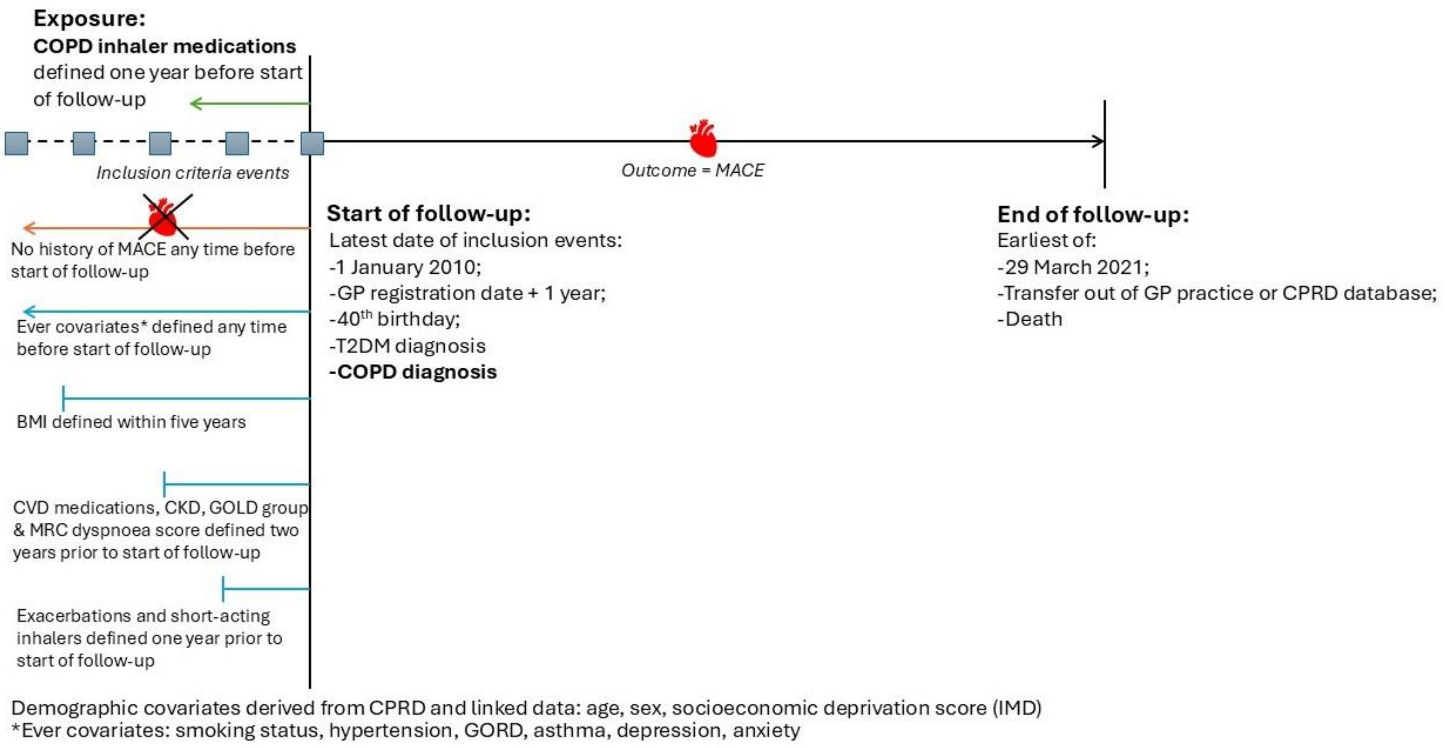

Obesity

Figure E3: Study diagrams for the Obesity cohort for (S3.1) COPD-specific, and (S3.2) ICS-specific questions

e3.1 COPD-specific study questions

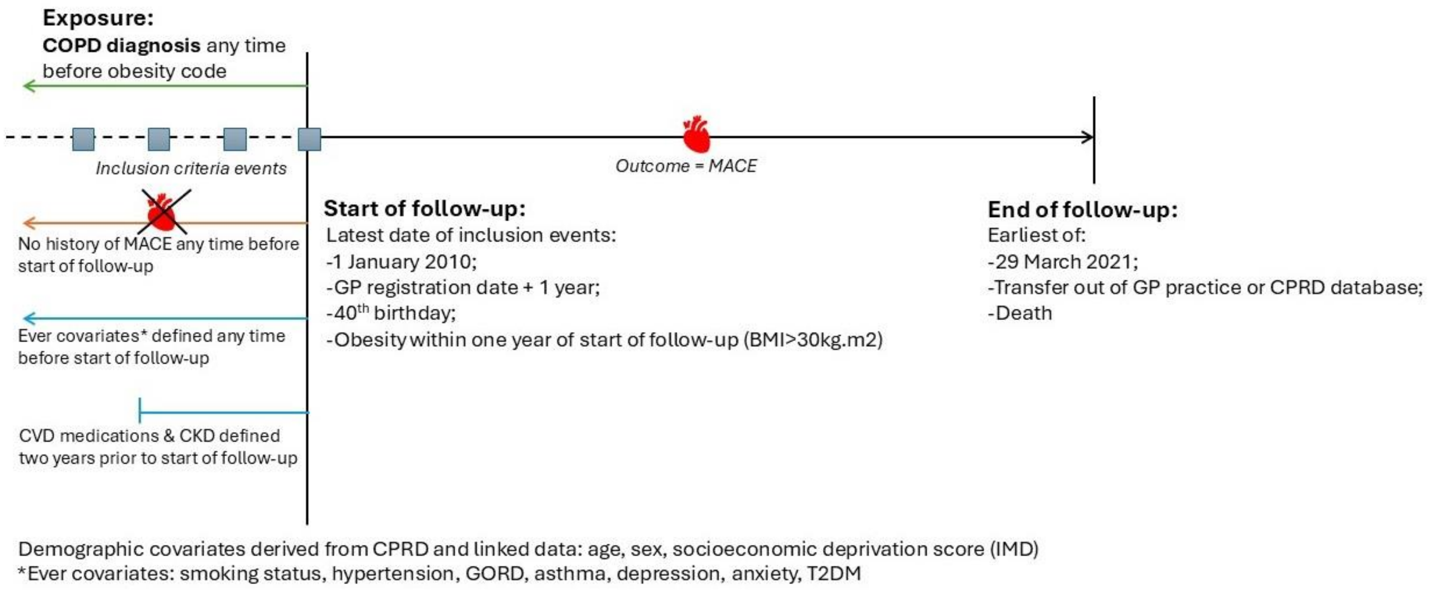

e3.2 ICS-specific study question

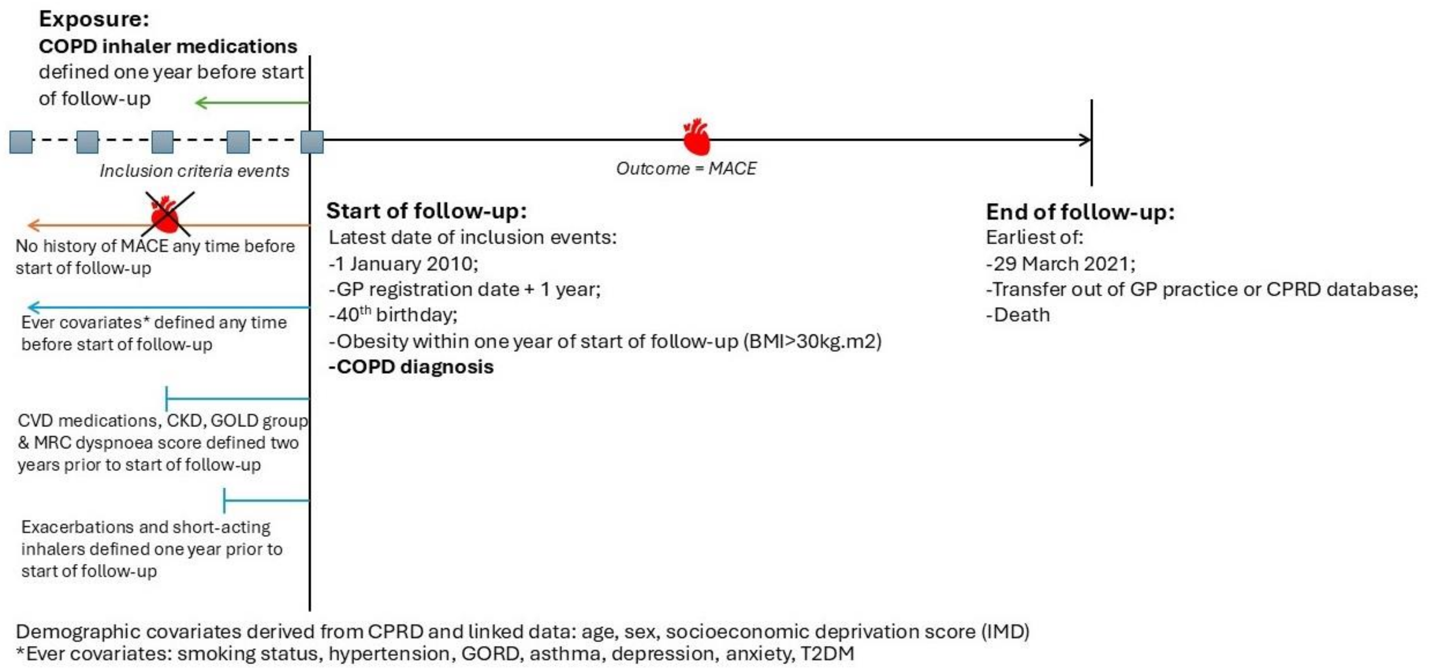

MACE history

Figure E4: Study diagrams for the MACE history cohort for (S4.1) COPD-specific, and (S4.2) ICS-specific questions

e4.1 COPD-specific study questions

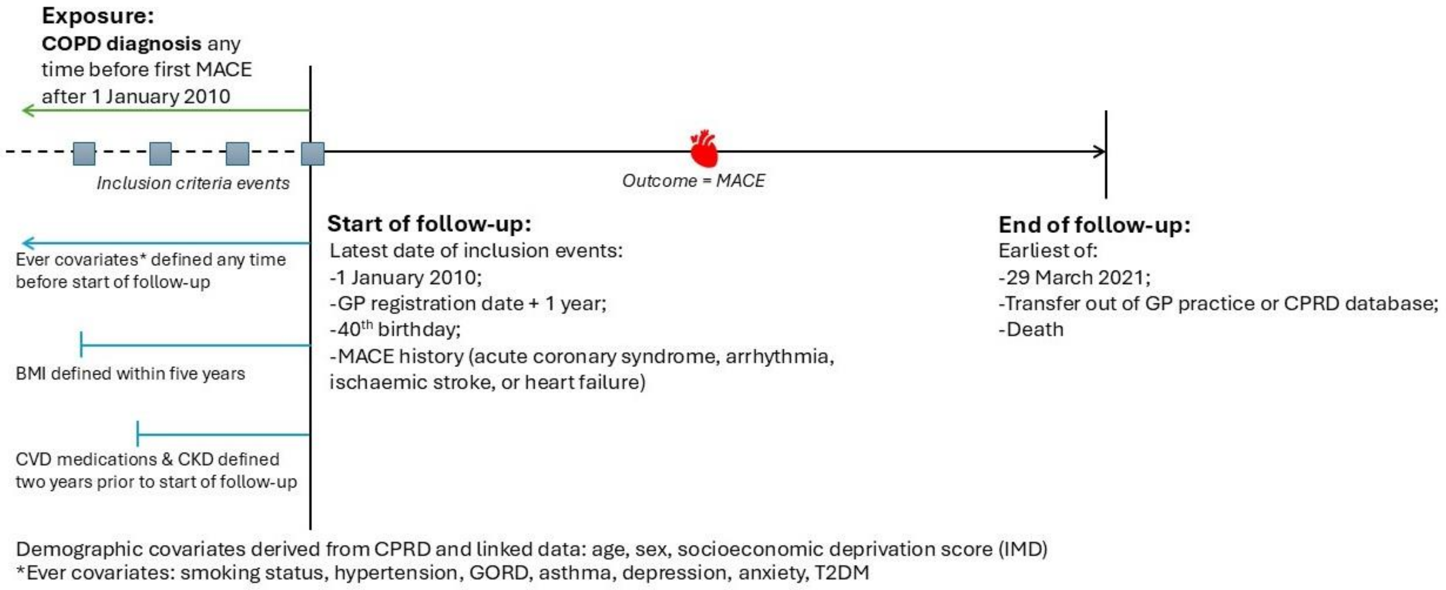

e4.2 ICS-specific study question

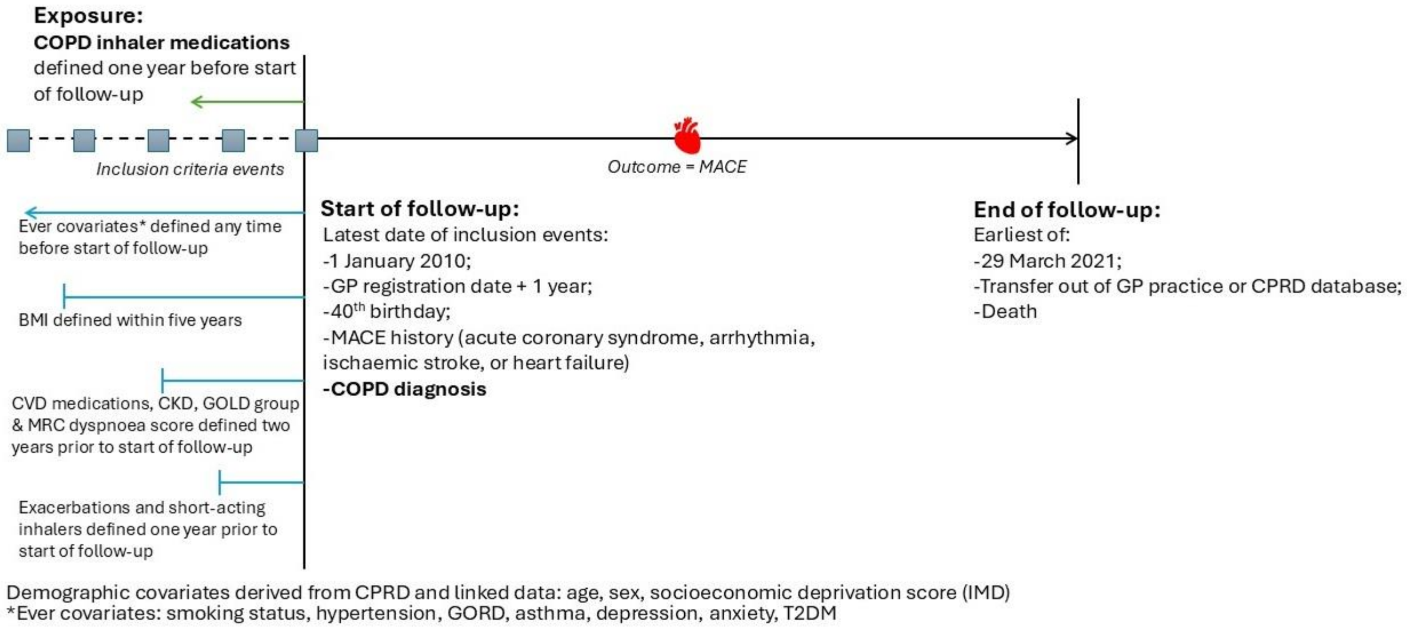

Age65+

Figure E5: Study diagrams for the Age65+ cohort for (S5.1) COPD-specific, and (S5.2) ICS-specific questions

e5.1 COPD-specific study questions

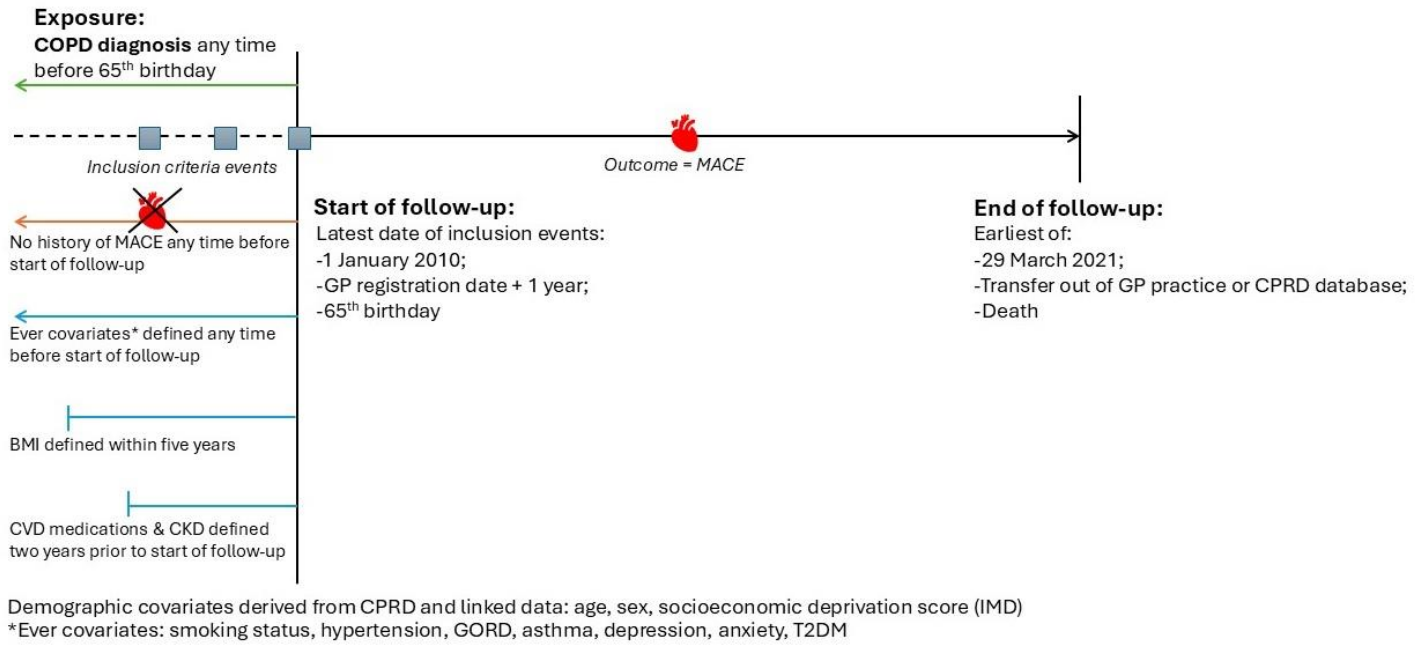

e5.2 ICS-specific study question

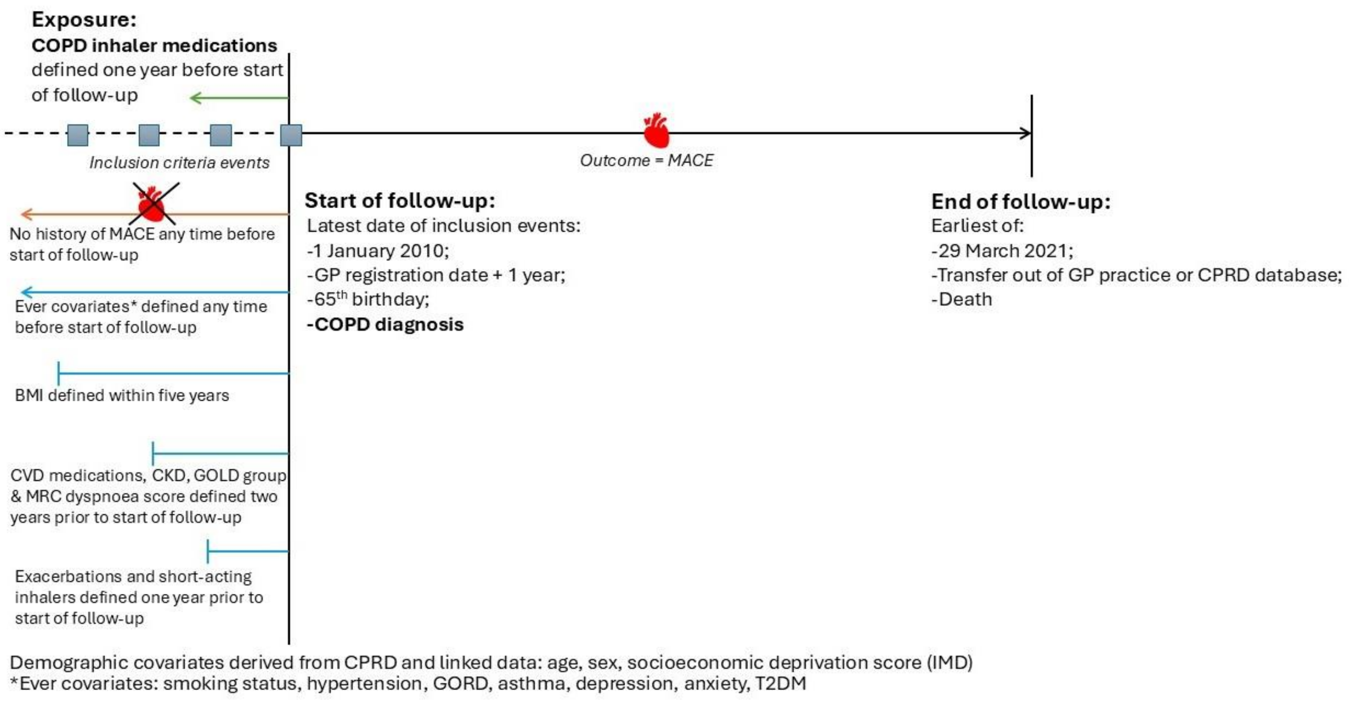

QRISK>10%

Figure E6 Study diagram for QRISK>10% cohort for COPD-specific study questions

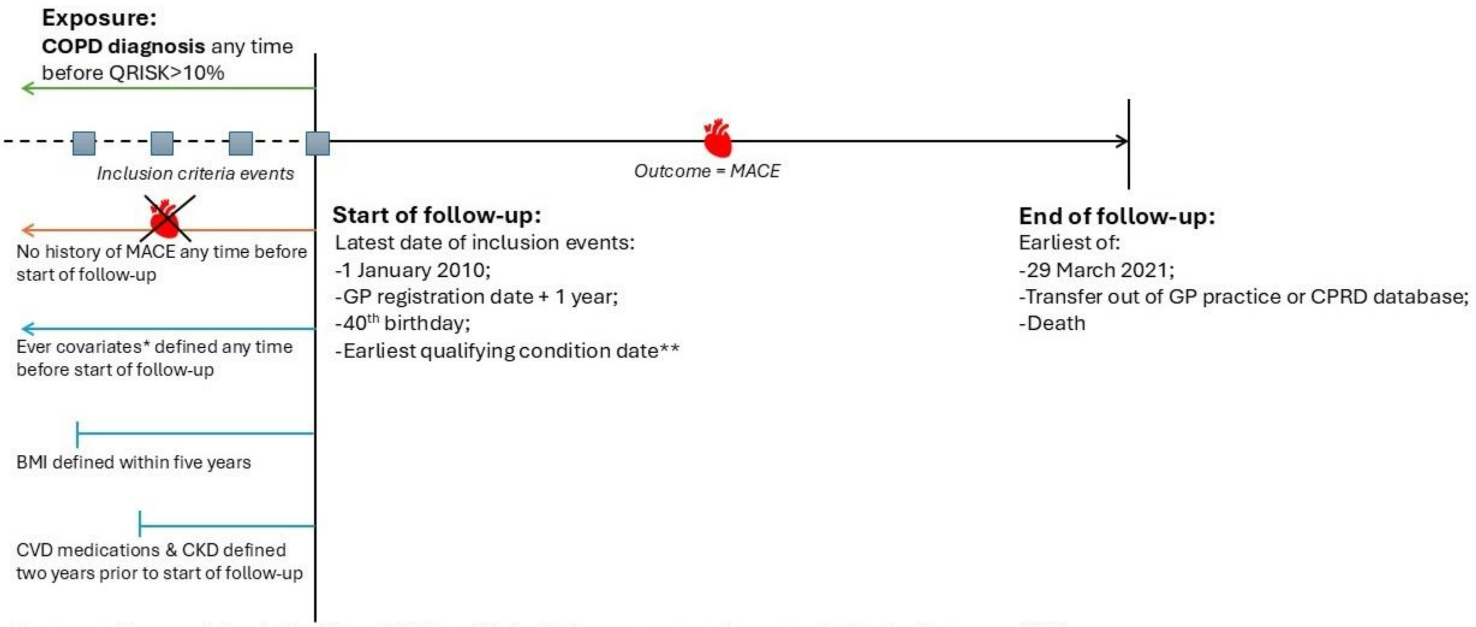

Demographic covariates derived from CPRD and linked data: age, sex, socioeconomic deprivation score (IMD)
*Ever covariates: smoking status, hypertension, GORD, asthma, depression, anxiety, T2DM
** Earliest qualifying condition date: Met eligibility for CKD, T2DM, Obesity, or Age65+ cohorts AND had a QRISK score of >10% on the date they became eligible for that cohort

VARIABLES DEFINITIONS LIST

Table E1: Definitions and dataset locations for cohorts, exposures, outcomes, and covariates

Variable	Dataset location	Definition
**COHORTS**		
**Chronic kidney disease**	CPRD Aurum	Two consecutive records of CKD based on serum creatinine (estimated glomerular filtration rate [eGFR])<60 mL/min/1.73m2 at least three months apart, based on the Chronic Kidney Disease Epidemiology Collaboration equation, and<br>- Eligible for linkage with secondary care data<br>- Deemed acceptable for research purposes by CPRD<br>- Age ≥40-years-old<br>- Data recorded in CPRD between 1st January 2010 and 29th March 2021<br>- Registered at their GP practice at least one year prior to start of follow-up<br>- No record of MACE (ACS, arrhythmia, stroke, or HF)
**Type II diabetes mellitus**	CPRD Aurum	Any record of T2DM, and<br>- Eligible for linkage with secondary care data<br>- Deemed acceptable for research purposes by CPRD<br>- Age ≥40-years-old<br>- Data recorded in CPRD between 1st January 2010 and 29th March 2021<br>- Registered at their GP practice at least one year prior to start of follow-up<br>- No record of MACE (ACS, arrhythmia, stroke, or HF)
**Obesity**	CPRD Aurum	A record of a BMI >30kg/m^2 within one year of meeting all other criteria (i.e., within one year of start of follow-up), and<br>- Eligible for linkage with secondary care data<br>- Deemed acceptable for research purposes by CPRD<br>- Age ≥40-years-old<br>- Data recorded in CPRD between 1st January 2010 and 29th March 2021<br>- Registered at their GP practice at least one year prior to start of follow-up<br>- No record of MACE (ACS, arrhythmia, stroke, or HF)
**Age65+**	CPRD Aurum	Age ≥65-years-old drawn from a random sample of a million people aged 65 years or above, and<br>- Eligible for linkage with secondary care data<br>- Deemed acceptable for research purposes by CPRD<br>- Data recorded in CPRD between 1st January 2010 and 29th March 2021<br>- Registered at their GP practice at least one year prior to start of follow-up<br>- No record of MACE (ACS, arrhythmia, stroke, or HF)
**MACE history**	CPRD Aurum	A record of either ACS, arrhythmias, stroke, or HF after 1st January 2010, and<br>- Eligible for linkage with secondary care data<br>- Deemed acceptable for research purposes by CPRD<br>- Data recorded in CPRD between 1st January 2010 and 29th March 2021<br>- Registered at their GP practice at least one year prior to start of follow-up
**QRISK>10%**	CPRD Aurum	Following meeting criteria for at least one of the cohorts above (CKD, T2DM, Obesity, Age65+, or MACE history), participants were screened using the peer-reviewed and publicly available QRISK

		algorithm (GitHub: https://github.com/emilyherrett/qrisk_cprd_aurum). Those who had a QRISK score of 10% or above were included.
**EXPOSURE: COPD**		
**Pre-existing**	CPRD Aurum	Main exposure<br>COPD diagnosis occurring any time prior to the condition that qualified the person to be within one of the cohorts<br>(i.e., CKD, T2DM, Obesity, Age65+, MACE history, and QRISK>10%)
**Incident**	CPRD Aurum	COPD diagnosis occurring within six weeks in either direction of the condition that qualified the person to be within one of the cohorts<br>(CKD, T2DM, and MACE history only)
**At risk: without infection**	CPRD Aurum	Meeting the following criteria:<br>- Absence of COPD diagnosis<br>- Smoking history<br>- Age>40-years-old<br>- Absence of asthma<br>(CKD, T2DM, Obesity, Age65+, MACE history)
**At risk: with infection**	CPRD Aurum	Meeting the following criteria:<br>- Absence of COPD diagnosis<br>- Smoking history<br>- Age>40-years-old<br>- Absence of asthma,<br>- >2 records of lower respiratory tract infections requiring antibiotics within two years before index date.<br>(CKD, T2DM, Obesity, Age65+, MACE history)
**EXPOSURE: ICSs**		
**ICSs**	CPRD Aurum	Any record of inhaled corticosteroids (ICS) in the year prior to index date:<br>- ICS monotherapy<br>- ICS-LABA combination<br>- ICS-LAMA combination<br>- ICS-LABA-LAMA (Triple therapy) combination<br>Combination of therapies are fixed or open
**Long-acting bronchodilators (control)**	CPRD Aurum	Any record of long-acting inhalers in the year prior to index date:<br>- LABA monotherapy<br>- LAMA monotherapy<br>- LABA-LAMA combination<br>Combination therapies are fixed and/or open
**OUTCOME: MACE**		Defined collectively as a secondary care record of either acute coronary syndrome, arrhythmias, heart failure, or ischaemic stroke.
**Acute coronary syndrome**	HES APC (first position ICD10 codes only)	Unstable angina<br>ST elevated myocardial infarction (STEMI)<br>Non-ST elevated myocardial infarction (non-STEMI)

**Arrhythmia**	HES APC (first position ICD10 codes only)	Atrial fibrillation / flutter<br>Cardiac arrest<br>Other arrhythmias
**Heart failure**	HES APC (first position ICD10 codes only)	HFpEF<br>HFrEF<br>Decompensated HF<br>Acute pulmonary oedema<br>Dilated cardiomyopathy<br>Takotsubo cardiomyopathy
**Ischaemic stroke**	HES APC (first position ICD10 codes only)	Ischaemic stroke
**Cardiovascular-specific death**	ONS (first position ICD10 codes only)	Any J.-code (Cardiovascular chapter); binary
**COVARIATES**		
**Age**	CPRD Aurum<br>Measured as the difference between index date and year of birth	Continuous variable
**Sex**	CPRD Aurum	Male or female
**Socioeconomic status**	Linked data – Index of Multiple Deprivation (IMD)	Index of Multiple Deprivation:<br>Quintiled measurement of deprivation, ranging from 1 (most deprived) to 5 (least deprived)
**Smoking status**	CPRD Aurum	Never-, current-, or ex-smoker (most recent patient record)
**Body Mass Index**	CPRD Aurum	BMI = weight (kg) / height^2 (m), most recent within the five years before start of follow-up<br>- Healthy weight (18.5 to 24.9 kg.m^{-2})<br>- Underweight (<18.5 kg.m^{-2})<br>- Overweight (25.0 to 29.9 kg.m^{-2})<br>- Obese (>30.0 kg.m^{-2})
**Depression**	CPRD Aurum	Binary; Any record prior to index date
**Anxiety**	CPRD Aurum	Binary; Any record prior to index date
**Gastro-oesophageal reflux disease**	CPRD Aurum	Binary; Any record prior to index date
**Hypertension**	CPRD Aurum	Binary; Any record prior to index date
**Type II diabetes mellitus**	CPRD Aurum	Binary; Any record prior to index date
**Chronic kidney disease**		Binary (with missing data recorded as such) defined in the two years prior to index date. A diagnosis was regarded as:<br>- Estimated glomerular filtration rate (eGFR) < 60mL/min/1.73m^2 (i.e., <60% kidney function)<br>Or<br>- A urine albumin:creatinine ratio (uACR) >3mg/mmol (i.e., proteinuria)
**Asthma**	CPRD Aurum	Binary; Any record prior to index date
**COPD exacerbation group**	CPRD Aurum<br>HES APC	Defined as one of the following categories based on the year preceding index date:<br>- None<br>- 1 moderate, 0 severe

		- 2 moderate, 0 severe<br>- 3+ moderate, 0 severe<br>- Any moderate, 1 severe<br>- Any moderate, 2+ severe<br>Moderate exacerbation is a record in primary care only; severe exacerbation required secondary care.<br>Methodology has been previously validated*
**MRC dyspnoea scale**	CPRD Aurum	Categorical variable based on the two years prior to index date or three months after index date:<br>- Grade 1: no dyspnoea except on strenuous exercise<br>- Grade 2: dyspnoea when walking up a short hill<br>- Grade 3: dyspnoea limits walking pace and participant stops to catch breath<br>- Grade 4: stops to catch breath after walking 100m on level ground<br>- Grade 5: dyspnoea limits leaving home and performing daily activities
**GOLD disease severity**	CPRD Aurum	Categorical variable on lung function (degree of airflow limitation) based on the two years prior to index date or three months after index date:<br>- GOLD1: FEV_1 predicted ≥80%<br>- GOLD2: FEV_1 predicted 50% to 80%<br>- GOLD3: FEV_1 predicted 30 to 50%<br>- GOLD4: FEV_1 predicted <30%
**Medications**		
**Cardiovascular medications**	CPRD Aurum	Binary variable defined as any record of the following medications from the British National Formulary Chapter 2, within the two years prior to index date:<br>- Diuretics<br>- Beta blockers<br>- Hypertension and heart failure drugs<br>- Anti-anginal medications, nitrates, and calcium channel blockers<br>- Anti-platelets<br>- Statins
**Short-acting bronchodilators**	CPRD Aurum	Binary variable defined as any record of short-acting inhalers in the year prior to index date:<br>- Short-acting beta agonists (SABA)<br>- Short-acting muscarinic antagonists (SAMA)<br>- Combination SABA-SAMA<br>Combination therapies are fixed and/or open

Codelists available at: https://github.com/NHLI-Respiratory-Epi/COPD-and-MACE-in-CRM-populations_codelists

* Rothnie KJ, Müllerová H, Hurst JR, Smeeth L, Davis K, Thomas SL, et. al. Validation of the recording of acute exacerbations of COPD in UK primary care electronic healthcare records. PLoS One. PLoS One. 2016;11:e0151357. doi: https://doi.org/10.1371/journal.pone.0151357

Abbreviations: HES: Hospital Episode Statistics | APC – Admitted Patient Care | CPRD – Clinical Practice Research Datalink | ONS – Office of National Statistics | MACE – major adverse cardiovascular events | BMI – body mass index | CKD – Chronic Kidney Disease | T2DM – type-II-diabetes | ACS – Acute Coronary Syndrome | stroke – Ischaemic Stroke | HF – heart failure | IMD – Index of Multiple Deprivation | HFpEF – heart failure with preserved ejection fraction | HFrEF – heart failure with reduced ejection fraction | COPD – chronic obstructive pulmonary disease | MRC – Medical Research Council | GOLD – Global Initiative for Obstructive Lung Disease

STUDY FLOW CHART AND BASELINE CHARACTERISTICS PER COHORT

Chronic Kidney Disease (CKD)

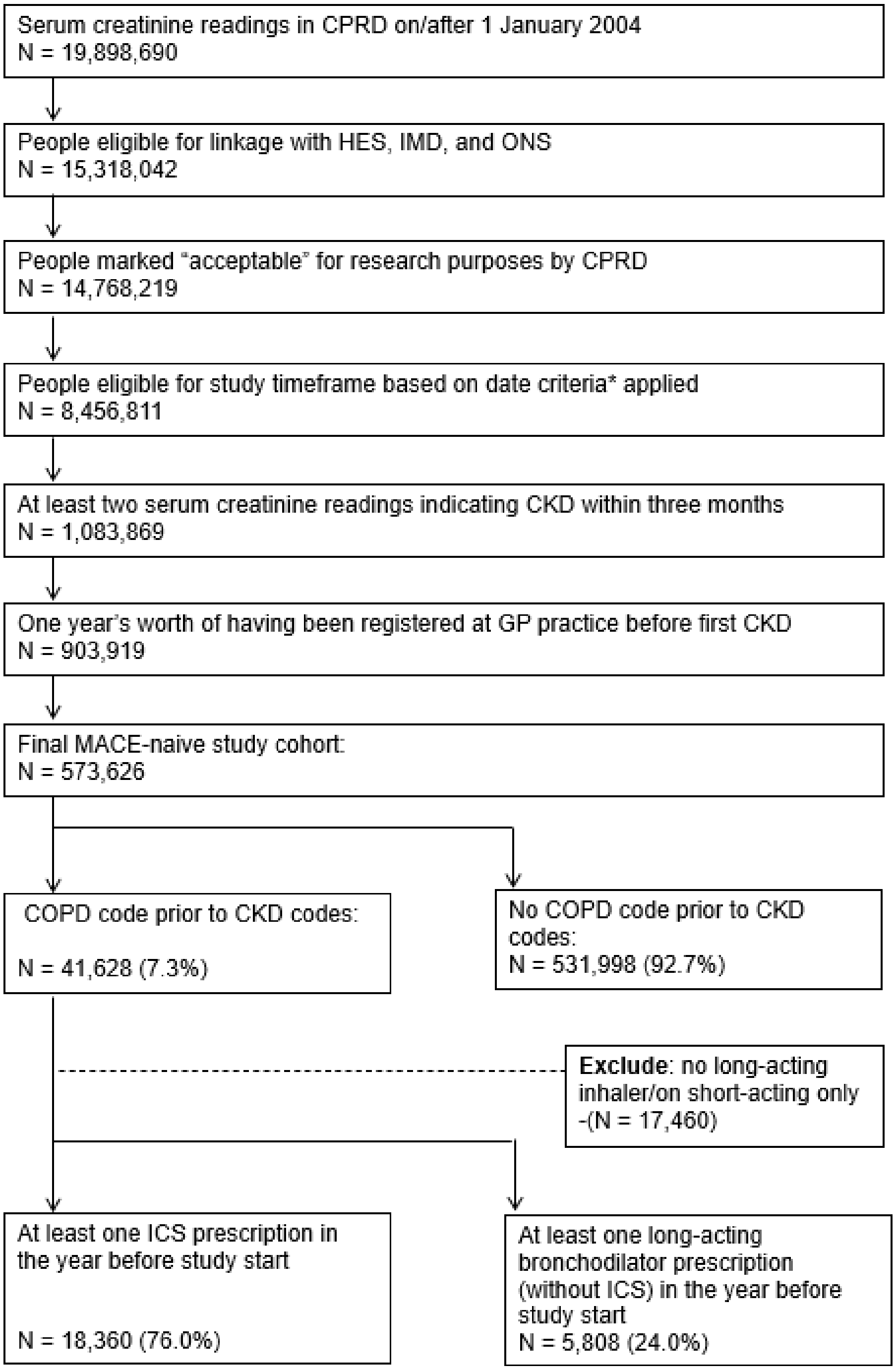

Figure E7: Inclusion and exclusion flow diagram for the CKD cohort

***Date criteria applied:** study start = latest of 1Jan2010, GP registration date + 1year, 40th birthday, and CKD diagnosis; study end = earliest of 29Mar2021, out-transfer (GP or CPRD), death date)

Abbreviations: CKD (chronic kidney disease), CPRD (Clinical Practice Research Datalink), HES (Hospital Episode Statistics), IMD (Index of Multiple Deprivation), ONS (Office for National Statistics), GP (general practitioner), MACE (major adverse cardiovascular event = acute coronary syndrome, arrhythmias, heart failure, or ischemic stroke), COPD (chronic obstructive pulmonary disease), ICS (inhaled corticosteroids)

Table E2: COPD-specific baseline characteristics for chronic kidney disease (CKD) population

Covariate relative to index (N (%) unless specified)	Total	COPD diagnosis	No COPD history	Incident COPD diagnosis	No COPD history vs incident	At risk of COPD (absence of infection)	No COPD history, nor at risk (vs at risk without infection)	At risk of COPD (presence of infection)	No COPD history, nor at risk (vs presence of infection)
	(N=573,626)	(N=41,628, 7.3%)	(N=531,998, 92.7%)	(N = 1,265, 0.2%)	(N = 531,210, 99.8%)	(N = 314,001, 10.3%)	(N = 217,997, 41.0%)	(N = 2,336, 0.4%)	(N = 529,662, 99.6%)
Study follow-up in years (median, IQR) All range (1 day to 12 years)	4.5 [2.0, 8.4]	3.2 [1.4, 6.1]	4.6 [2.0, 8.6]	3.9 [1.7, 7.0]	4.6 [2.0, 8.6]	4.7 [2.1, 8.6]	4.6 [2.0, 8.7]	3.4 [1.4, 6.7]	4.7 [2.0, 8.6]
**Age at entry (mean, SD)**	76.2 (10.5)	77.3 (8.5)	76.1 (10.6)	76.2 (8.6)	76.1 (10.6)	76.5 (10.3)	75.7 (11.2)	77.8 (10.5)	76.1 (10.6)
**Sex**									
Male	211,297 (36.8)	18,173 (43.7)	193,124 (36.3)	563 (44.5)	192,764 (36.3)	126,541 (40.3)	66,583 (30.5)	885 (37.9)	192,239 (36.3)
Female	362,329 (63.2)	23,455 (56.3)	338,874 (63.7)	702 (55.5)	338,446 (63.7)	187,460 (59.7)	151,414 (69.5)	1,451 (62.1)	337,423 (63.7)
**Smoking status**									
Never-smoker	111,878 (19.5)	1,790 (4.30)	110,088 (20.7)	73 (5.8)	110,046 (20.7)	--	110,088 (50.5)	--	110,088 (20.8)
Ex-smoker	371,490 (64.8)	28,484 (68.4)	343,006 (64.5)	762 (60.2)	342,536 (64.5)	255,998 (81.5)	87,008 (39.8)	1,796 (76.9)	341,210 (64.4)
Current smoker	90,258 (15.7)	11,354 (27.3)	78,904 (14.8)	430 (34.0)	78,628 (14.8)	58,003 (18.5)	20,901 (9.6)	540 (23.1)	78,364 (14.8)
**Index of Multiple Deprivation quintile**									
1 Least Deprived	120,091 (20.9)	6,299 (15.1)	113,792 (21.4)	212 (16.8)	113,676 (21.4)	67,152 (21.4)	46,640 (21.4)	426 (18.2)	113,366 (21.4)
2	123,842 (21.6)	7,747 (18.6)	116,095 (21.8)	249 (19.7)	115,935 (21.8)	69,392 (22.1)	46,703 (21.4)	481 (20.6)	115,614 (21.8)
3	114,717 (20.0)	8,005 (19.2)	106,712 (20.1)	238 (18.8)	106,562 (20.1)	63,370 (20.2)	43,342 (19.9)	448 (19.2)	106,264 (20.1)
4	111,920 (19.5)	8,873 (21.3)	103,047 (19.4)	272 (21.5)	102,870 (19.4)	60,033 (19.1)	43,014 (19.7)	491 (21.0)	102,556 (19.4)
5 Most Deprived	102,546 (17.9)	10,680 (25.7)	91,866 (17.3)	293 (23.2)	91,681 (17.3)	53,775 (17.1)	38,091 (17.5)	489 (20.9)	91,377 (17.3)
Missing/Unknown	510 (0.09)	24 (0.06)	486 (0.09)	###	486 (0.1)	279 (0.1)	207 (0.1)	###	485 (0.1)
**Comorbidities***									
BMI (kg/m^2)									
Normal (18.5 to <25)	132,538 (23.1)	11,448 (27.5)	121,090 (22.8)	358 (28.3)	120,864 (22.8)	71,977 (22.9)	49,113 (22.5)	473 (20.3)	120,617 (22.8)
Underweight (<18.5)	9,908 (1.73)	1,376 (3.31)	8,532 (1.60)	40 (3.2)	8,508 (1.6)	5,183 (1.7)	3,349 (1.5)	46 (2.0)	8,486 (1.6)
Overweight (25 to <30)	183,233 (31.9)	13,674 (32.9)	169,559 (31.9)	394 (31.2)	169,317 (31.9)	100,711 (32.1)	68,848 (31.6)	674 (28.9)	168,885 (31.9)
Obese (30+)	157,606 (27.5)	12,943 (31.1)	144,663 (27.2)	358 (28.3)	144,440 (27.2)	82,557 (26.3)	62,106 (28.5)	729 (31.2)	143,934 (27.2)
Missing/Unknown	90,341 (15.8)	2,187 (5.25)	88,154 (16.6)	115 (9.1)	88,081 (16.6)	53,573 (17.1)	34,581 (15.9)	414 (17.7)	87,740 (16.6)
Type II Diabetes	123,716 (21.6)	8,793 (21.1)	114,923 (21.6)	215 (17.0)	114,788 (21.6)	71,278 (22.7)	43,645 (20.0)	71,278 (22.7)	43,645 (20.0)
Hypertension	411,590 (71.8)	28,093 (67.5)	383,497 (72.1)	884 (66.7)	382,990 (72.1)	229,015 (72.9)	154,482 (70.9)	1,575 (67.4)	381,922 (72.1)
GORD	98,169 (17.1)	9,298 (22.3)	88,871 (16.7)	205 (16.2)	88,750 (16.7)	51,720 (16.5)	37,151 (17.0)	540 (23.1)	88,331 (16.7)
Asthma	153,808 (26.8)	24,475 (58.8)	129,333 (24.3)	553 (43.7)	128,985 (24.3)	--	129,333 (59.3)	--	129,333 (24.4)
Depression	86,793 (15.1)	8,448 (20.3)	78,345 (14.7)	228 (18.0)	78,209 (14.7)	45,932 (14.6)	32,413 (14.9)	469 (20.1)	77,876 (14.7)

Covariate relative to index (N (%) unless specified)	Total	COPD diagnosis	No COPD history	Incident COPD diagnosis	No COPD history vs incident	At risk of COPD (absence of infection)	No COPD history, nor at risk (vs at risk without infection)	At risk of COPD (presence of infection)	No COPD history, nor at risk (vs presence of infection)
Anxiety	73,536 (12.8)	6,750 (16.2)	66,786 (12.6)	193 (15.3)	66,675 (12.6)	39,215 (12.5)	27,571 (12.7)	416 (17.8)	66,370 (12.5)
**Cardiovascular-related prescriptions****									
Any	495,444 (86.4)	36,412 (87.5)	459,032 (86.3)	1,087 (85.9)	458,363 (86.3)	273,522 (87.1)	185,510 (85.1)	2,038 (87.2)	456,994 (86.3)
Diuretics	265,234 (46.2)	21,490 (51.6)	243,744 (45.8)	653 (51.6)	243,339 (45.8)	144,169 (45.9)	99,575 (45.7)	1,158 (49.6)	242,586 (45.8)
Beta blockers	140,572 (24.5)	5,393 (13.0)	135,179 (25.4)	232 (18.3)	135,033 (25.4)	86,865 (27.7)	48,314 (22.2)	594 (25.4)	134,585 (25.4)
Hypertension, HF drugs	343,476 (59.9)	24,276 (58.3)	319,200 (60.0)	774 (58.8)	318,741 (60.0)	191,283 (60.9)	127,917 (58.7)	1,357 (58.1)	317,843 (60.0)
Nitrates, CCBs,antianginals	246,960 (43.1)	18,251 (43.8)	228,709 (43.0)	555 (43.9)	228,366 (43.0)	137,361 (43.8)	91,348 (41.9)	947 (40.5)	227,762 (43.0)
Antiplatelets	195,952 (34.2)	15,118 (36.3)	180,834 (34.0)	458 (36.2)	180,529 (34.0)	112,388 (35.8)	68,446 (31.4)	898 (38.4)	179,936 (34.0)
Statins	284,575 (49.6)	21,602 (51.9)	262,973 (49.4)	654 (51.7)	262,559 (49.4)	159,816 (50.9)	103,157 (47.3)	1,164 (49.8)	261,809 (49.4)

Comorbidities defined as 'ever' in history, except for BMI (defined as nearest to study start within past 5 years), cardiovascular medications (defined within the 2 years before study start)

** n(%) described for cardiovascular prescription categories may not be mutually exclusive, as they can be taken in combination elsewhere in the table (e.g., patient prescribed a diuretic and statins)
Too few to report (as per CPRD patient confidentiality policy)

Abbreviations: MACE = major adverse cardiovascular event (acute coronary syndrome; arrhythmias; heart failure; ischaemic stroke); COPD = chronic obstructive pulmonary disease; BMI = body mass index; GORD = gastro-oesophageal reflux disease; HF = heart failure; CCB = calcium channel blocker

Table E3: ICS-specific baseline characteristics for CKD population with COPD

Covariate relative to index (N (%) unless specified)	Total (N=24,168)	ICS (N=18,360, 76.0%)	Non-ICS (N=5,808, 24.0%)
Study follow-up in years (median, IQR)	3.1 [1.3, 5.7]	3.2 [1.4, 5.9]	2.7 [1.2, 5.1]
All range (1 day to 12 years)			
**Age at entry (mean, SD)**	77.2 (8.2)	77.3 (8.2)	76.9 (8.2)
**Sex**			
Male	10,662 (44.1)	7,962 (43.4)	2,700 (46.5)
Female	13,506 (55.9)	10,398 (56.6)	3,108 (53.5)
**Smoking status**			
Never-smoker	804 (3.3)	657 (3.6)	147 (2.5)
Ex-smoker	16,833 (69.7)	13,013 (70.9)	3,820 (65.8)
Current smoker	6,531 (27.0)	4,690 (25.5)	1,841 (31.7)
**Index of Multiple Deprivation quintile**			
1 Least Deprived	3,650 (15.1)	2,754 (15.0)	896 (15.4)
2	4,456 (18.4)	3,345 (18.2)	1,111 (19.1)
3	4,626 (19.1)	3,500 (19.1)	1,126 (19.4)
4	5,121 (21.2)	3,896 (21.2)	1,225 (21.1)
5 Most Deprived	6,301 (26.1)	4,855 (26.4)	1,446 (24.9)
Missing/Unknown	14 (0.1)	10 (0.1)	###
**Comorbidities**			
BMI (kg/m^2)			
Normal (18.5 to <25)	6,682 (27.7)	5,089 (27.7)	1,593 (27.4)
Underweight (<18.5)	784 (3.2)	593 (3.2)	191 (3.3)
Overweight (25 to <30)	7,920 (32.8)	5,965 (32.5)	1,955 (33.7)
Obese (30+)	7,754 (32.1)	5,923 (32.3)	1,831 (31.5)
Missing/Unknown	1,028 (4.3)	790 (4.3)	238 (4.1)
Type II Diabetes	5,034 (20.8)	3,755 (20.5)	1,279 (22.0)
Hypertension	16,190 (67.0)	12,330 (67.2)	3,860 (66.5)
GORD	5,467 (22.6)	4,157 (22.6)	1,310 (22.6)
Asthma	14,896 (61.6)	12,468 (67.9)	2,428 (41.8)
Depression	4,950 (20.5)	3,726 (20.3)	1,224 (21.1)
Anxiety	3,875 (16.0)	2,948 (16.1)	927 (16.0)
**Cardiovascular-related prescriptions****			
Any	21,271 (88.0)	16,249 (88.5)	5,022 (86.5)
Diuretics	12,971 (53.7)	10,287 (56.0)	2,684 (46.2)
Beta blockers	2,864 (11.9)	1,879 (10.2)	985 (17.0)
Hypertension and heart failure drugs	14,129 (58.5)	10,872 (59.2)	3,257 (56.1)
Nitrates, CCBs, other antianginals	10,535 (43.6)	8,029 (43.7)	2,506 (43.2)
Antiplatelets	8,713 (36.1)	6,614 (36.0)	2,099 (36.1)
Statins	12,674 (52.4)	9,468 (51.6)	3,206 (55.2)

Covariate relative to index (N (%) unless specified)	Total	ICS	Non-ICS
**COPD characteristics**			
COPD exacerbations			
None	15,926 (65.9)	11,608 (63.2)	4,318 (74.4)
1 moderate, 0 severe	3,644 (15.1)	2,888 (15.7)	756 (13.0)
2 moderate, 0 severe	1,142 (4.7)	949 (5.2)	193 (3.3)
3+ moderate, 0 severe	748 (3.1)	649 (3.5)	99 (1.7)
Any moderate, 1 severe	2,084 (8.6)	1,725 (9.4)	359 (6.2)
Any moderate, 2+ severe	624 (2.6)	541 (3.0)	83 (1.4)
MRC dyspnoea group			
1	2,027 (8.4)	1,426 (7.8)	601 (10.4)
2	7,121 (29.5)	4,917 (26.8)	2,204 (38.0)
3	6,827 (28.3)	5,160 (28.1)	1,667 (28.7)
4	4,307 (17.8)	3,625 (19.7)	682 (11.7)
5	1,038 (4.3)	888 (4.8)	150 (2.6)
Missing/Unknown	2,848 (11.8)	2,344 (12.8)	504 (8.7)
GOLD group			
1 (Mild)	4,318 (17.9)	2,970 (16.2)	1,348 (23.2)
2 (Moderate)	9,702 (40.1)	7,069 (38.5)	2,633 (45.3)
3 (Severe)	4,642 (19.2)	3,890 (21.2)	752 (13.0)
4 (Very severe)	797 (3.3)	686 (3.7)	111 (1.9)
Missing/Unknown	4,709 (19.5)	3,745 (20.4)	964 (16.6)
Short-acting medications			
None	2,970 (12.3)	1,734 (9.4)	1,236 (21.3)
SABA	19,318 (79.9)	15,104 (82.3)	4,214 (72.6)
SAMA	237 (1.0)	178 (1.0)	59 (1.0)
SABA-SAMA	1,643 (6.8)	1,344 (7.3)	299 (5.2)

ICS = ICS monotherapy, ICS-LABA, ICS-LAMA, or ICS-LABA-LAMA (triple therapy)
Non-ICS = any long-acting bronchodilator, i.e., LABA, LAMA, or LABA-LAMA

Comorbidities defined as 'ever' in history, except for BMI (defined as nearest to study start within past 5 years), cardiovascular medications (defined within the 2 years before study start), MRC dyspnoea group (defined within two years before and three months after study start), COPD exacerbations (defined in the year before study start), and short-acting inhalers (defined in the year before study start)

** n(%) described for cardiovascular prescription categories may not be mutually exclusive, as they can be taken in combination elsewhere in the table (e.g., patient prescribed a diuretic and statins)
Too few to report (as per CPRD patient confidentiality policy)

Abbreviations: ICS = inhaled corticosteroids; LABA = long-acting beta agonist; LAMA = long-acting muscarinic antagonist; MACE = major adverse cardiovascular event (acute coronary syndrome; arrhythmias; heart failure; ischaemic stroke); COPD = chronic obstructive pulmonary disease; BMI = body mass index; GORD = gastro-oesophageal reflux disease; CCB = calcium channel blocker; MRC = Medical Research Council; GOLD = Global Initiative for Chronic Obstructive Lung Disease; SABA = short-acting beta agonist; SAMA = short-acting muscarinic antagonist

Type-II Diabetes Mellitus (T2DM)

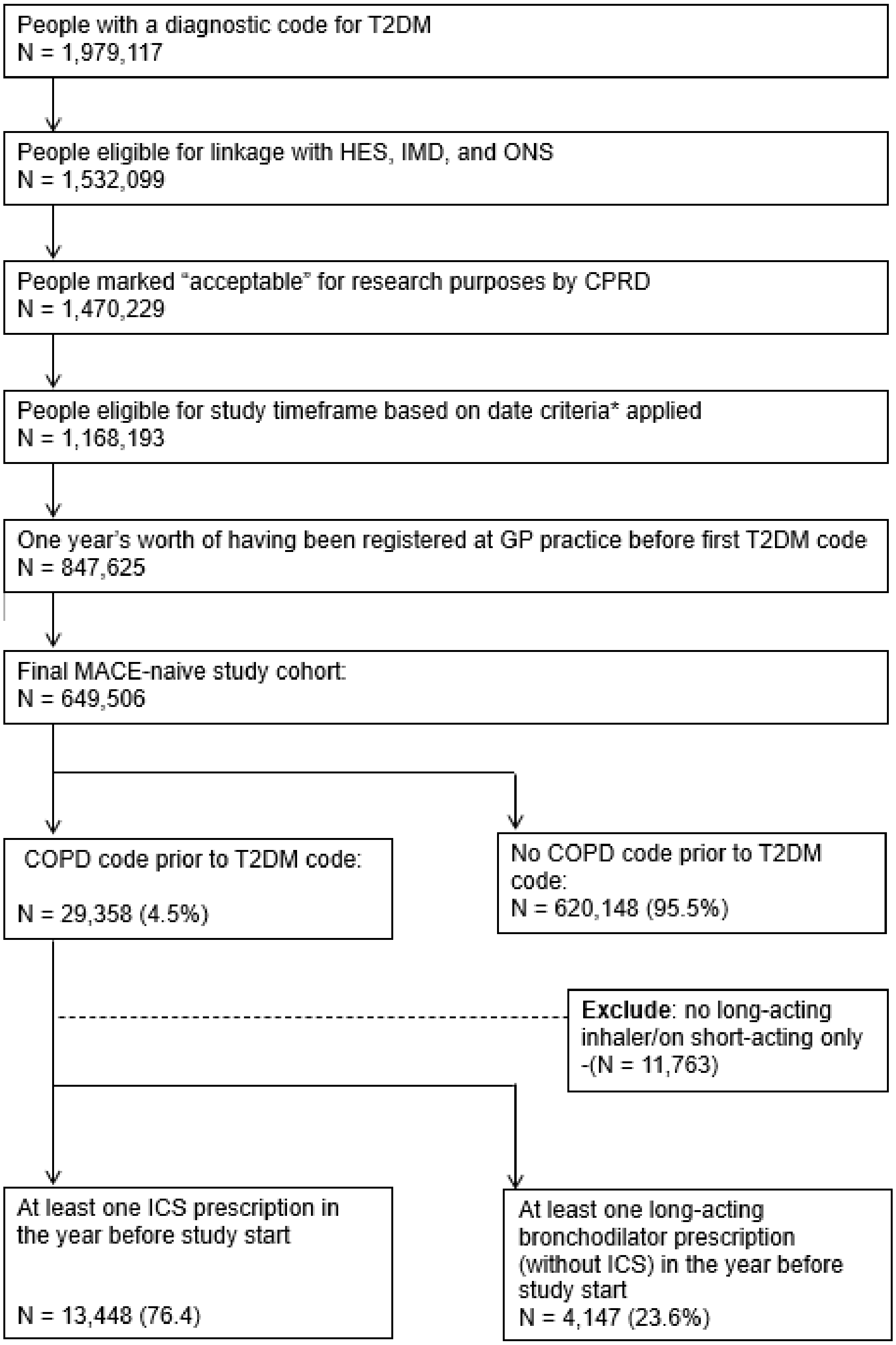

Figure E8: Inclusion and exclusion flow diagram for the T2DM cohort

***Date criteria applied:** study start = latest of 1Jan2010, GP registration date + 1year, 40th birthday, and T2DM diagnosis; study end = earliest of 29Mar2021, out-transfer (GP or CPRD), death date)

Abbreviations: T2DM (Type-II diabetes mellitus), CPRD (Clinical Practice Research Datalink), HES (Hospital Episode Statistics), IMD (Index of Multiple Deprivation), ONS (Office for National Statistics), GP (general practitioner), MACE (major adverse cardiovascular event = acute coronary syndrome, arrhythmias, heart failure, or ischemic stroke), COPD (chronic obstructive pulmonary disease), ICS (inhaled corticosteroids)

Table E4: COPD-specific baseline characteristics for T2DM population

Covariate relative to index (N (%) unless specified)	Total (N=649,506)	COPD diagnosis (N=29,358, 4.5%)	No COPD history (N=620,148, 95.5%)	Incident COPD diagnosis (N = 1,222, 0.2%)	No COPD history vs incident (N=619,585, 99.8%)	At risk of COPD (absence of infection) (N=351,015, 56.6%)	No COPD history, nor at risk (vs at risk without infection) (N=269,133, 43.4%)	At risk of COPD (presence of infection) (N=1,716, 0.3%)	No COPD history, nor at risk (vs presence of infection) (N=618,432, 99.7%)
Study follow-up in years (median, IQR) All range (1 day to 12 years)	5.7 [2.5, 9.9]	3.8 [1.7, 6.9]	5.7 [2.6, 10.1]	4.4 [2.0, 7.8]	5.7 [2.6, 10.1]	5.9 [2.7, 10.3]	5.5 [2.5, 9.8]	4.2 [2.1, 7.5]	5.7 [2.6, 10.1]
**Age at entry (mean, SD)**	62.5 (12.8)	68.5 (10.3)	62.2 (12.8)	65.4 (10.6)	62.2 (12.8)	63.4 (12.6)	60.6 (12.9)	65.1 (12.8)	62.2 (12.8)
**Sex**									
Male	349,830 (53.9)	15,726 (53.6)	344,104 (53.9)	697 (57.0)	333,768 (53.9)	204,587 (58.3)	129,517 (48.1)	812 (47.3)	333,292 (53.9)
Female	299,676 (46.1)	13,632 (46.4)	286,044 (46.1)	525 (43.0)	285,817 (46.1)	146,428 (41.7)	139,616 (51.9)	904 (52.7)	285,140 (46.1)
**Smoking status**									
Never-smoker	115,639 (17.8)	832 (2.83)	114,807 (18.5)	45 (3.7)	114,787 (18.5)	--	114,807 (42.7)	--	114,807 (18.6)
Ex-smoker	385,301 (59.3)	17,563 (59.8)	367,738 (59.3)	627 (51.3)	367,457 (59.3)	256,319 (73.0)	111,419 (41.4)	1,167 (68.0)	366,571 (59.3)
Current smoker	148,566 (22.8)	10,963 (37.3)	137,603 (22.2)	550 (45.0)	137,341 (22.2)	94,696 (27.0)	42,907 (15.9)	549 (32.0)	137,054 (22.2)
**Index of Multiple Deprivation quintile**									
1 Least Deprived	109,689 (16.9)	3,461 (11.8)	106,228 (17.1)	148 (12.1)	106,151 (17.1)	62,197 (17.7)	44,031 (16.4)	255 (14.9)	105,973 (17.1)
2	121,007 (18.6)	4,713 (16.1)	116,294 (18.8)	205 (16.8)	116,211 (18.8)	68,444 (19.5)	47,850 (17.8)	310 (18.1)	115,984 (18.8)
3	125,621 (19.3)	5,338 (18.2)	120,283 (19.4)	224 (18.3)	120,162 (19.4)	68,921 (19.6)	51,362 (19.1)	339 (19.8)	119,944 (19.4)
4	142,756 (22.0)	6,827 (23.3)	135,929 (21.9)	256 (21.0)	135,832 (21.9)	74,495 (21.2)	61,434 (22.8)	355 (20.7)	135,574 (21.9)
5 Most Deprived	149,852 (23.1)	8,990 (30.6)	140,862 (22.7)	389 (31.8)	140,667 (22.7)	76,652 (21.8)	64,210 (23.9)	455 (26.5)	140,407 (22.7)
Missing/Unknown	581 (0.1)	29 (0.1)	552 (0.1)	###	552 (0.1)	306 (0.1)	246 (0.1)	###	550 (0.1)
**Comorbidities**									
BMI (kg/m^2)									
Normal (18.5 to <25)	73,332 (11.3)	3,692 (12.6)	69,640 (11.2)	132 (10.8)	69,583 (11.2)	40,591 (11.6)	29,049 (10.8)	129 (7.5)	69,511 (11.2)
Underweight (<18.5)	3,348 (0.5)	400 (1.4)	2,948 (0.5)	###	2,944 (0.5)	1,860 (0.5)	1,088 (0.4)	12 (0.7)	2,936 (0.5)
Overweight (25 to <30)	187,086 (28.8)	8,086 (27.5)	179,000 (28.9)	337 (27.6)	178,845 (28.9)	103,627 (29.5)	75,373 (28.0)	413 (24.1)	178,587 (28.9)
Obese (30+)	332,327 (51.2)	16,367 (55.8)	315,960 (51.0)	663 (54.3)	315,666 (51.0)	173,238 (49.4)	142,722 (53.0)	980 (57.1)	314,980 (50.9)
Missing/Unknown	53,413 (8.2)	813 (2.8)	52,600 (8.5)	84 (6.9)	52,547 (8.5)	31,699 (9.0)	20,901 (7.8)	182 (10.6)	52,418 (8.5)
Chronic Kidney Disease									
Yes	87,653 (13.5)	4,730 (16.1)	82,923 (13.4)	156 (12.8)	82,854 (13.4)	50,744 (14.5)	32,179 (12.0)	257 (15.0)	82,666 (13.4)
No	514,757 (79.3)	22,909 (78.0)	491,848 (79.3)	982 (80.4)	491,399 (79.3)	274,808 (78.3)	217,040 (80.6)	1,375 (80.1)	490,473 (79.3)
Missing	47,096 (7.3)	1,719 (5.9)	45,377 (7.3)	84 (6.9)	45,332 (7.3)	25,463 (7.3)	19,914 (7.4)	84 (4.9)	45,293 (7.3)
Hypertension	370,490 (57.0)	17,284 (58.9)	353,206 (57.0)	663 (54.3)	352,911 (57.0)	205,689 (58.6)	147,517 (54.8)	975 (56.8)	352,231 (57.0)
GORD	100,378 (15.5)	6,855 (23.4)	93,523 (15.1)	228 (18.7)	93,412 (15.1)	51,206 (14.6)	42,317 (15.7)	457 (26.6)	93,066 (15.1)
Asthma	201,828 (31.1)	18,388 (62.6)	183,440 (29.6)	605 (49.5)	183,180 (29.6)	--	183,440 (68.2)	--	183,440 (29.7)

Covariate relative to index (N (%) unless specified)	Total	COPD diagnosis	No COPD history	Incident COPD diagnosis	No COPD history vs incident	At risk of COPD (absence of infection)	No COPD history, nor at risk (vs at risk without infection)	At risk of COPD (presence of infection)	No COPD history, nor at risk (vs presence of infection)
Depression	117,336 (18.1)	7,730 (26.3)	109,606 (17.7)	295 (24.1)	109,474 (17.7)	60,484 (17.2)	49,122 (18.3)	445 (25.9)	109,161 (17.7)
Anxiety	82,944 (12.8)	5,587 (19.0)	77,357 (12.5)	214 (17.5)	77,261 (12.5)	42,739 (12.2)	34,618 (12.9)	343 (20.0)	77,014 (12.5)
**Cardiovascular-related prescriptions****									
Any	502,889 (77.4)	24,462 (83.3)	478,427 (77.2)	952 (77.9)	477,994 (77.2)	278,077 (79.2)	200,350 (74.4)	1,374 (80.1)	477,053 (77.1)
Diuretics	184,953 (28.5)	11,656 (39.7)	173,297 (27.9)	430 (35.2)	173,096 (27.9)	100,923 (28.8)	72,374 (26.9)	570 (33.2)	172,727 (27.9)
Beta blockers	111,397 (17.2)	3,258 (11.1)	108,139 (17.4)	162 (13.3)	108,056 (17.4)	70,156 (20.0)	38,983 (14.1)	373 (21.7)	107,766 (17.4)
Hypertension, HF drugs	337,200 (51.9)	14,503 (49.4)	322,697 (52.0)	602 (49.3)	322,430 (52.0)	190,107 (54.2)	132,590 (49.3)	826 (48.1)	321,871 (52.1)
Nitrates, CCBs,antianginals	218,374 (33.6)	11,674 (39.8)	206,700 (33.3)	440 (36.0)	206,494 (33.3)	120,675 (34.4)	86,025 (32.0)	603 (35.1)	206,097 (33.3)
Antiplatelets	198,843 (30.6)	9,393 (32.0)	189,450 (30.6)	358 (29.3)	189,282 (30.6)	116,743 (33.3)	72,707 (27.0)	450 (26.2)	189,000 (30.6)
Statins	366,569 (56.4)	17,271 (58.8)	349,298 (56.3)	671 (54.9)	348,995 (56.3)	206,907 (59.0)	142,391 (52.9)	903 (52.6)	348,395 (56.3)

Comorbidities defined as 'ever' in history except for BMI (defined as nearest to study start within past 5 years), cardiovascular medications (defined in the last two years), and CKD (nearest within past 2 years, as eGFR mL/min as a measure of kidney function, e.g., 40 mL/min eGFR = 40% kidney function)

** n(%) described for cardiovascular prescription categories may not be mutually exclusive, as they can be taken in combination elsewhere in the table (e.g., patient prescribed a diuretic and statins)

Abbreviations: MACE = major adverse cardiovascular event (acute coronary syndrome; arrhythmias; heart failure; ischaemic stroke); COPD = chronic obstructive pulmonary disease; BMI = body mass index; CKD = chronic kidney disease; eGFR = estimated glomerular filtration rate; GORD = gastro-oesophageal reflux disease; HF = heart failure; CCB = calcium channel blocker

Table E5: ICS-specific baseline characteristics for T2DM population with COPD

Covariate relative to index (N (%) unless specified)	Total (N=17,595)	ICS (N=13,448, 76.4%)	Non-ICS (N=4,147, 23.6%)
Study follow-up in years (median, IQR) All range (1 day to 12 years)	3.6 [1.6, 6.4]	3.7 [1.6, 6.6]	3.2 [1.5, 5.7]
**Age at entry (mean, SD)**	68.8 (10.0)	68.8 (10.0)	68.5 (9.8)
**Sex**			
Male	9,343 (53.1)	7,013 (52.2)	2,330 (56.2)
Female	8,252 (46.9)	6,435 (47.9)	1,817 (43.8)
**Smoking status**			
Never-smoker	341 (1.9)	283 (2.1)	58 (1.4)
Ex-smoker	10,678 (60.7)	8,307 (61.8)	2,371 (57.2)
Current smoker	6,576 (37.4)	4,858 (36.1)	1,718 (41.4)
**Index of Multiple Deprivation quintile**			
1 Least Deprived	2,007 (11.4)	1,536 (11.4)	471 (11.4)
2	2,780 (15.8)	2,124 (15.8)	656 (15.8)
3	3,128 (17.8)	2,389 (17.8)	739 (17.8)
4	4,086 (23.2)	3,116 (23.2)	970 (23.4)
5 Most Deprived	5,577 (31.7)	4,268 (31.7)	1,309 (31.6)
Missing/Unknown	17 (0.1)	15 (0.1)	###
**Comorbidities**			
BMI (kg/m^2)			
Normal (18.5 to <25)	2,242 (12.7)	1,765 (13.1)	477 (11.5)
Underweight (<18.5)	264 (1.5)	216 (1.6)	48 (1.2)
Overweight (25 to <30)	4,730 (26.9)	3,626 (27.0)	1,104 (26.6)
Obese (30+)	9,996 (56.8)	7,559 (56.2)	2,437 (58.8)
Missing/Unknown	363 (2.1)	282 (2.1)	81 (2.0)
Chronic Kidney Disease			
Yes	2,791 (15.9)	2,159 (16.1)	632 (15.2)
No	13,802 (78.4)	10,499 (78.1)	3,303 (79.7)
Missing	1,002 (5.7)	790 (5.9)	212 (5.1)
Hypertension	10,268 (58.4)	7,842 (58.3)	2,426 (58.5)
GORD	4,210 (23.9)	3,249 (24.2)	961 (23.2)
Asthma	11,510 (65.4)	9,634 (71.6)	1,876 (45.2)
Depression	4,838 (27.5)	3,741 (27.8)	1,097 (26.5)
Anxiety	3,440 (19.6)	2,637 (19.6)	803 (19.4)
**Cardiovascular-related prescriptions**			
Any	14,766 (83.9)	11,338 (84.3)	3,428 (82.6)
Diuretics	7,344 (41.7)	5,874 (43.7)	1,470 (35.5)
Beta blockers	1,793 (10.2)	1,181 (8.8)	612 (14.8)
Hypertension and heart failure drugs	8,610 (48.9)	6,654 (49.5)	1,956 (47.2)
Nitrates, CCBs, other antianginals	7,061 (40.1)	5,381 (40.0)	1,680 (40.5)
Antiplatelets	5,597 (31.8)	4,340 (32.3)	1,257 (30.3)
Statins	10,370 (58.9)	7,922 (58.9)	2,448 (59.0)

Covariate relative to index (N (%) unless specified)	Total	ICS	Non-ICS
**COPD characteristics**			
COPD exacerbations			
None	11,271 (64.1)	8,200 (61.0)	3,071 (74.1)
1 moderate, 0 severe	2,774 (15.8)	2,196 (16.3)	578 (13.9)
2 moderate, 0 severe	877 (5.0)	754 (5.6)	123 (3.0)
3+ moderate, 0 severe	641 (3.6)	553 (4.1)	88 (2.1)
Any moderate, 1 severe	1,484 (8.4)	1,244 (9.3)	240 (5.8)
Any moderate, 2+ severe	548 (3.11)	501 (3.7)	47 (1.1)
MRC dyspnoea group			
1	1,559 (8.9)	1,063 (7.9)	496 (12.0)
2	5,208 (29.6)	3,599 (26.8)	1,609 (38.8)
3	5,050 (28.7)	3,892 (28.9)	1,158 (27.9)
4	3,116 (17.7)	2,614 (19.4)	502 (12.1)
5	792 (4.5)	704 (5.2)	88 (2.1)
Missing	1,870 (10.6)	1,576 (11.7)	294 (7.1)
GOLD group			
1 (Mild)	2,676 (15.2)	1,907 (14.2)	769 (18.5)
2 (Moderate)	7,614 (43.3)	5,454 (40.6)	2,160 (52.1)
3 (Severe)	3,519 (20.0)	2,912 (21.7)	607 (14.6)
4 (Very severe)	844 (4.8)	753 (5.6)	91 (2.2)
Missing	2,942 (16.7)	2,422 (18.0)	520 (12.5)
Short-acting medications			
None	1,643 (9.3)	937 (7.0)	706 (17.0)
SABA	14,843 (84.4)	11,628 (86.5)	3,215 (77.5)
SAMA	113 (0.6)	75 (0.6)	38 (0.9)
SABA-SAMA	996 (5.7)	808 (6.0)	188 (4.5)

ICS = ICS monotherapy, ICS-LABA, ICS-LAMA, or ICS-LABA-LAMA (triple therapy)
Non-ICS = any long-acting bronchodilator, i.e., LABA, LAMA, or LABA-LAMA

Comorbidities defined as 'ever' in history, except for BMI (defined as nearest to study start within past 5 years), cardiovascular medications (defined within the 2 years before study start), CKD (defined as nearest to study start within 2 years, as a measure of kidney function, e.g., 40 mL/min eGFR = 40% kidney function), MRC dyspnoea group (defined within two years before and three months after study start), COPD exacerbations (defined in the year before study start), and short-acting inhalers (defined in the year before study start)

** n(%) described for cardiovascular prescription categories may not be mutually exclusive, as they can be taken in combination elsewhere in the table (e.g., patient prescribed a diuretic and statins)
Too few to report (as per CPRD patient confidentiality policy)

Abbreviations: ICS = inhaled corticosteroids; LABA = long-acting beta agonist; LAMA = long-acting muscarinic antagonist; MACE = major adverse cardiovascular event (acute coronary syndrome; arrhythmias; heart failure; ischaemic stroke); COPD = chronic obstructive pulmonary disease; BMI = body mass index; CKD = chronic kidney disease; GORD = gastro-oesophageal reflux disease; CCB = calcium channel blocker; MRC = Medical Research Council; GOLD = Global Initiative for Chronic Obstructive Lung Disease; SABA = short-acting beta agonist; SAMA = short-acting muscarinic antagonist

Obesity

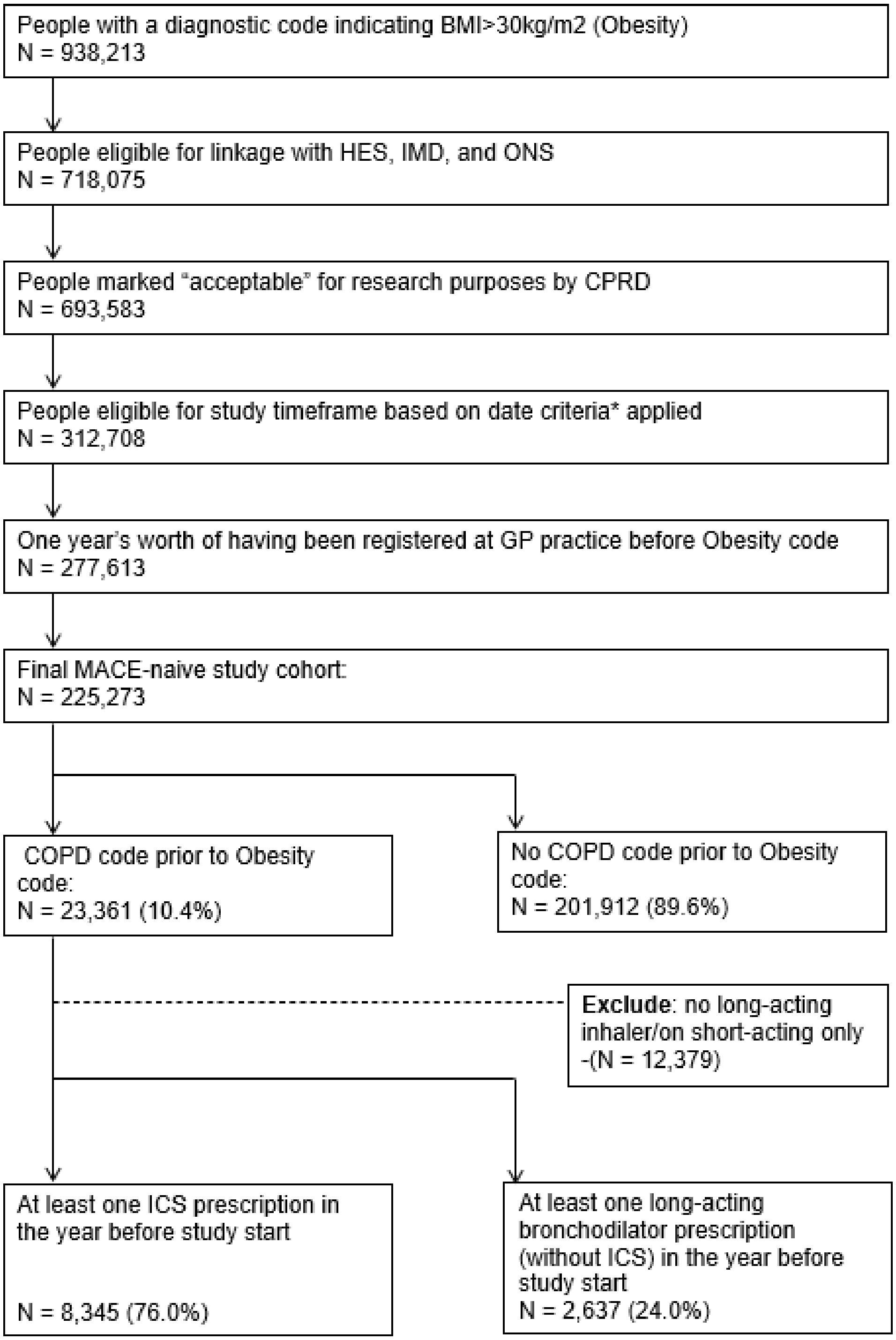

Figure E9 Inclusion and exclusion flow diagram for the Obesity cohort
***Date criteria applied:** study start = latest of 1Jan2010, GP registration date + 1year, 40th birthday, and Obesity code date; study end = earliest of 29Mar2021, out-transfer (GP or CPRD), death date)
Abbreviations: BMI (body mass index), CPRD (Clinical Practice Research Datalink), HES (Hospital Episode Statistics), IMD (Index of Multiple Deprivation), ONS (Office for National Statistics), GP (general practitioner), MACE (major adverse cardiovascular event = acute coronary syndrome, arrhythmias, heart failure, or ischemic stroke), COPD (chronic obstructive pulmonary disease), ICS (inhaled corticosteroids)

Table E6: COPD-specific baseline characteristics for Obesity (BMI>30kg/m^2) population

Covariate relative to index (N (%) unless specified)	Total (N=225,273)	COPD diagnosis (N=23,361, 10.4%)	No COPD history (N=201,912, 89.6%)	At risk of COPD (absence of infection) (N=108,524, 53.8%)	No COPD history, nor at risk (vs at risk without infection) (N=93,388, 46.3%)	At risk of COPD (presence of infection) (N=625, 0.3%)	No COPD history, nor at risk (vs presence of infection) (N=201,287, 99.7%)
Study follow-up in years (median, IQR) All range (1 day to 12 years)	4.7 [2.2, 8.4]	4.0 [2.1, 6.9]	4.8 [2.2, 8.5]	5.2 [2.3, 9.0]	4.4 [2.1, 7.8]	3.4 [1.8, 5.8]	4.8 [2.2, 8.5]
**Age at entry (mean, SD)**	57.9 (11.7)	64.3 (10.7)	57.2 (11.6)	58.3 (11.8)	55.8 (11.3)	60.6 (12.2)	57.2 (11.6)
**Sex**							
Male	94,730 (42.1)	10,960 (46.9)	83,770 (41.5)	49,711 (45.8)	34,059 (36.5)	229 (36.6)	83,541 (41.5)
Female	130,543 (58.0)	12,401 (53.1)	118,142 (58.5)	58,813 (54.2)	59,329 (63.5)	396 (63.4)	117,746 (58.5)
**Smoking status**							
Never-smoker	38,770 (17.2)	789 (3.4)	37,981 (18.8)	--	37,981 (40.7)	--	37,981 (18.9)
Ex-smoker	134,920 (59.9)	13,427 (57.5)	121,493 (60.2)	79,854 (73.6)	41,639 (44.6)	450 (72.0)	121,043 (60.1)
Current smoker	51,583 (22.9)	9,145 (39.2)	42,438 (21.0)	28,670 (26.4)	13,768 (14.7)	175 (28.0)	42,263 (21.0)
**Index of Multiple Deprivation quintile**							
1 Least Deprived	35,229 (15.6)	2,463 (10.5)	32,766 (16.2)	17,024 (15.7)	15,742 (16.9)	88 (14.1)	32,678 (16.2)
2	42,196 (18.7)	3,716 (15.9)	38,480 (19.1)	20,821 (19.2)	17,659 (18.9)	106 (17.0)	38,374 (19.1)
3	42,474 (18.9)	4,144 (17.7)	38,330 (19.0)	21,033 (19.4)	17,297 (18.5)	119 (19.0)	38,211 (19.0)
4	53,481 (23.7)	5,718 (24.5)	47,763 (23.7)	26,359 (24.3)	21,404 (22.9)	136 (21.8)	47,627 (23.7)
5 Most Deprived	51,746 (23.0)	7,299 (31.2)	44,447 (22.0)	23,215 (21.4)	21,232 (22.7)	176 (28.2)	44,271 (22.0)
Missing/Unknown	147 (0.1)	21 (0.1)	126 (0.1)	72 (0.1)	54 (0.1)	###	126 (0.1)
**Comorbidities***							
Chronic Kidney Disease							
Yes	15,469 (6.9)	2,505 (10.7)	12,964 (6.4)	7,758 (7.2)	5,206 (5.6)	67 (10.7)	12,897 (6.4)
No	148,503 (65.9)	16,410 (70.3)	132,093 (65.4)	69,369 (63.9)	62,724 (67.2)	494 (79.0)	131,599 (65.4)
Missing	61,301 (27.2)	4,446 (19.0)	56,855 (28.2)	31,397 (28.9)	25,458 (27.3)	64 (10.2)	56,791 (28.2)
Type II Diabetes	50,910 (22.6)	5,863 (25.1)	45,047 (22.3)	25,774 (23.8)	19,273 (20.6)	208 (33.3)	44,839 (22.3)
Hypertension	101,296 (45.0)	12,686 (54.3)	88,610 (43.9)	50,069 (46.1)	38,541 (41.3)	326 (52.2)	88,284 (43.9)
GORD	37,724 (16.8)	5,251 (22.5)	32,473 (16.1)	16,405 (15.1)	16,068 (17.2)	165 (26.4)	32,308 (16.1)
Asthma	79,959 (35.5)	13,102 (56.1)	66,857 (33.1)	--	66,857 (71.6)	--	66,857 (33.2)

Covariate relative to index (N (%) unless specified)	Total	COPD diagnosis	No COPD history	At risk of COPD (absence of infection)	No COPD history, nor at risk (vs at risk without infection)	At risk of COPD (presence of infection)	No COPD history, nor at risk (vs presence of infection)
Depression	51,006 (22.6)	6,787 (29.1)	44,219 (21.9)	22,437 (20.7)	21,782 (23.3)	187 (29.9)	44,032 (21.9)
Anxiety	33,417 (14.8)	4,459 (19.1)	28,958 (14.3)	14,460 (13.3)	14,498 (15.5)	138 (22.1)	28,820 (14.3)
**Cardiovascular-related prescriptions****							
Any	130,094 (57.8)	16,970 (72.6)	113,124 (56.0)	63,838 (58.8)	49,286 (52.8)	442 (70.7)	112,682 (56.0)
Diuretics	48,227 (21.4)	7,432 (31.8)	40,795 (20.2)	23,001 (21.2)	17,794 (19.1)	178 (28.5)	40,617 (20.2)
Beta blockers	28,793 (12.8)	2,876 (12.3)	25,917 (12.8)	16,517 (15.2)	9,400 (10.1)	111 (17.8)	25,806 (12.8)
Hypertension, HF drugs	83,617 (37.1)	10,726 (45.9)	72,891 (36.1)	41,501 (38.2)	31,390 (33.6)	288 (46.1)	72,603 (36.1)
Nitrates,CCBs,Antianginals	56,462 (25.1)	7,819 (33.5)	48,643 (24.1)	27,461 (25.3)	21,182 (22.7)	186 (29.8)	48,547 (24.1)
Antiplatelets	32,300 (14.3)	5,281 (22.6)	27,019 (13.4)	16,787 (15.5)	10,232 (11.0)	116 (18.6)	26,903 (13.4)
Statins	77,196 (34.3)	11,332 (48.5)	65,864 (32.6)	38,382 (35.4)	27,482 (29.4)	290 (46.4)	65,574 (32.6)

Comorbidities defined as ‘ever’ in history except for cardiovascular medications (defined in the last two years), and CKD (nearest within past 2 years, as eGFR mL/min as a measure of kidney function, e.g., 40 mL/min eGFR = 40% kidney function)

** n(%) described for cardiovascular prescription categories may not be mutually exclusive, as they can be taken in combination elsewhere in the table (e.g., patient prescribed a diuretic and statins)

Abbreviations: BMI = body mass index, MACE = major adverse cardiovascular event (acute coronary syndrome; arrhythmias; heart failure; ischaemic stroke); COPD = chronic obstructive pulmonary disease; CKD = chronic kidney disease; eGFR = estimated glomerular filtration rate; GORD = gastro-oesophageal reflux disease; CCB = calcium channel blocker

Table E7: ICS-specific baseline characteristics for Obesity (BMI>30kg/m^2) population with COPD

**Covariate relative to index (N (%) unless specified)**	**Total (N=10,982)**	**ICS (N=8,345, 76.0%)**	**Non-ICS (N=2,637, 24.0%)**
Study follow-up in years (median, IQR) All range (1 day to 12 years)	3.4 [1.8, 5.6]	3.6 [1.9, 6.0]	2.7 [1.6, 4.5]
**Age at entry (mean, SD)**	65.7 (10.3)	65.1 (10.5)	67.4 (9.6)
**Sex**			
Male	5,015 (45.7)	3,638 (43.6)	1,377 (52.2)
Female	5,967 (54.3)	4,707 (56.4)	1,260 (47.8)
**Smoking status**			
Never-smoker	231 (2.1)	194 (2.3)	37 (1.4)
Ex-smoker	6,579 (59.9)	5,032 (60.3)	1,547 (58.7)
Current smoker	4,172 (38.0)	3,119 (37.4)	1,053 (39.9)
**Index of Multiple Deprivation quintile**			
1 Least Deprived	1,108 (10.1)	806 (9.7)	302 (11.5)
2	1,717 (15.6)	1,246 (14.9)	471 (17.9)
3	1,898 (17.3)	1,444 (17.3)	454 (17.2)
4	2,651 (24.1)	2,042 (24.5)	609 (23.1)
5 Most Deprived	3,597 (32.8)	2,801 (33.6)	796 (30.2)
Missing/Unknown	11 (0.1)	###	###
**Comorbidities***			
Chronic Kidney Disease			
Yes	1,292 (11.8)	927 (11.1)	365 (13.8)
No	7,858 (71.6)	5,973 (71.6)	1,885 (71.5)
Missing	1,832 (16.7)	1,445 (17.3)	387 (14.7)
Type II Diabetes	2,824 (25.7)	2,098 (25.1)	726 (27.5)
Hypertension	6,194 (56.4)	4,663 (55.9)	1,531 (58.1)
GORD	2,701 (24.6)	2,087 (25.0)	614 (23.3)
Asthma	7,370 (67.1)	6,226 (74.6)	1,144 (43.4)
Depression	3,413 (31.1)	2,648 (31.7)	765 (29.0)
Anxiety	2,255 (20.5)	1,721 (20.6)	534 (20.3)
**Cardiovascular-related prescriptions****			
Any	8,413 (76.6)	6,330 (75.9)	2,083 (79.0)
Diuretics	3,911 (35.6)	3,065 (36.7)	846 (32.1)
Beta blockers	1,146 (10.4)	757 (9.1)	389 (14.8)
Hypertension and HF drugs	5,236 (47.7)	3,938 (47.2)	1,298 (49.2)
Nitrates, CCBs, other antianginals	3,921 (35.7)	2,947 (35.3)	974 (36.9)
Antiplatelets	2,598 (23.7)	1,967 (23.6)	631 (23.9)
Statins	5,588 (50.9)	4,119 (49.4)	1,469 (55.7)

Covariate relative to index (N (%) unless specified)	Total	ICS	Non-ICS
**COPD characteristics**			
COPD exacerbations			
None	7,643 (69.6)	5,657 (67.8)	1,986 (75.3)
1 moderate, 0 severe	1,683 (15.3)	1,308 (15.7)	375 (14.2)
2 moderate, 0 severe	556 (5.1)	452 (5.4)	104 (3.9)
3+ moderate, 0 severe	321 (2.9)	280 (3.4)	41 (1.6)
Any moderate, 1 severe	635 (5.8)	515 (6.2)	120 (4.6)
Any moderate, 2+ severe	144 (1.3)	133 (1.6)	11 (0.4)
MRC dyspnoea group			
1	968 (8.8)	642 (7.7)	326 (12.4)
2	3,257 (29.7)	2,188 (26.2)	1,069 (40.5)
3	2,987 (29.7)	2,241 (26.9)	746 (28.3)
4	1,612 (14.7)	1,330 (15.9)	282 (10.7)
5	327 (3.0)	285 (3.4)	42 (1.6)
Missing	1,831 (16.7)	1,659 (19.9)	172 (6.5)
GOLD group			
1 (Mild)	2,226 (20.3)	1,562 (18.7)	664 (25.2)
2 (Moderate)	4,754 (43.3)	3,416 (40.9)	1,338 (50.7)
3 (Severe)	1,600 (14.6)	1,301 (15.6)	299 (11.3)
4 (Very severe)	241 (2.2)	209 (2.5)	32 (1.2)
Missing	2,161 (19.7)	1,857 (22.3)	304 (11.5)
Short-acting medications			
None	1,114 (10.1)	649 (7.8)	465 (17.6)
SABA	9,476 (86.3)	7,392 (88.6)	2,084 (79.0)
SAMA	36 (0.3)	19 (0.2)	17 (0.6)
SABA-SAMA	356 (3.2)	285 (3.4)	71 (2.7)

ICS = ICS monotherapy, ICS-LABA, ICS-LAMA, or ICS-LABA-LAMA (triple therapy)
Non-ICS = any long-acting bronchodilator, i.e., LABA, LAMA, or LABA-LAMA

Comorbidities defined as 'ever' in history, cardiovascular medications (defined within the 2 years before study start), CKD (defined as nearest to study start within 2 years, as a measure of kidney function, e.g., 40 mL/min eGFR = 40% kidney function), MRC dyspnoea group (defined within two years before and three months after study start), COPD exacerbations (defined in the year before study start), and short-acting inhalers (defined in the year before study start)

** n(%) described for cardiovascular prescription categories may not be mutually exclusive, as they can be taken in combination elsewhere in the table (e.g., patient prescribed a diuretic and statins)
Too few to report (as per CPRD patient confidentiality policy)

Abbreviations: BMI = body mass index; ICS = inhaled corticosteroids; LABA = long-acting beta agonist; LAMA = long-acting muscarinic antagonist; MACE = major adverse cardiovascular event (acute coronary syndrome; arrhythmias; heart failure; ischaemic stroke); COPD = chronic obstructive pulmonary disease; HF = heart failure; CKD = chronic kidney disease; GORD = gastro-oesophageal reflux disease; CCB = calcium channel blocker; MRC = Medical Research Council; GOLD = Global Initiative for Chronic Obstructive Lung Disease; SABA = short-acting beta agonist; SAMA = short-acting muscarinic antagonist

MACE history

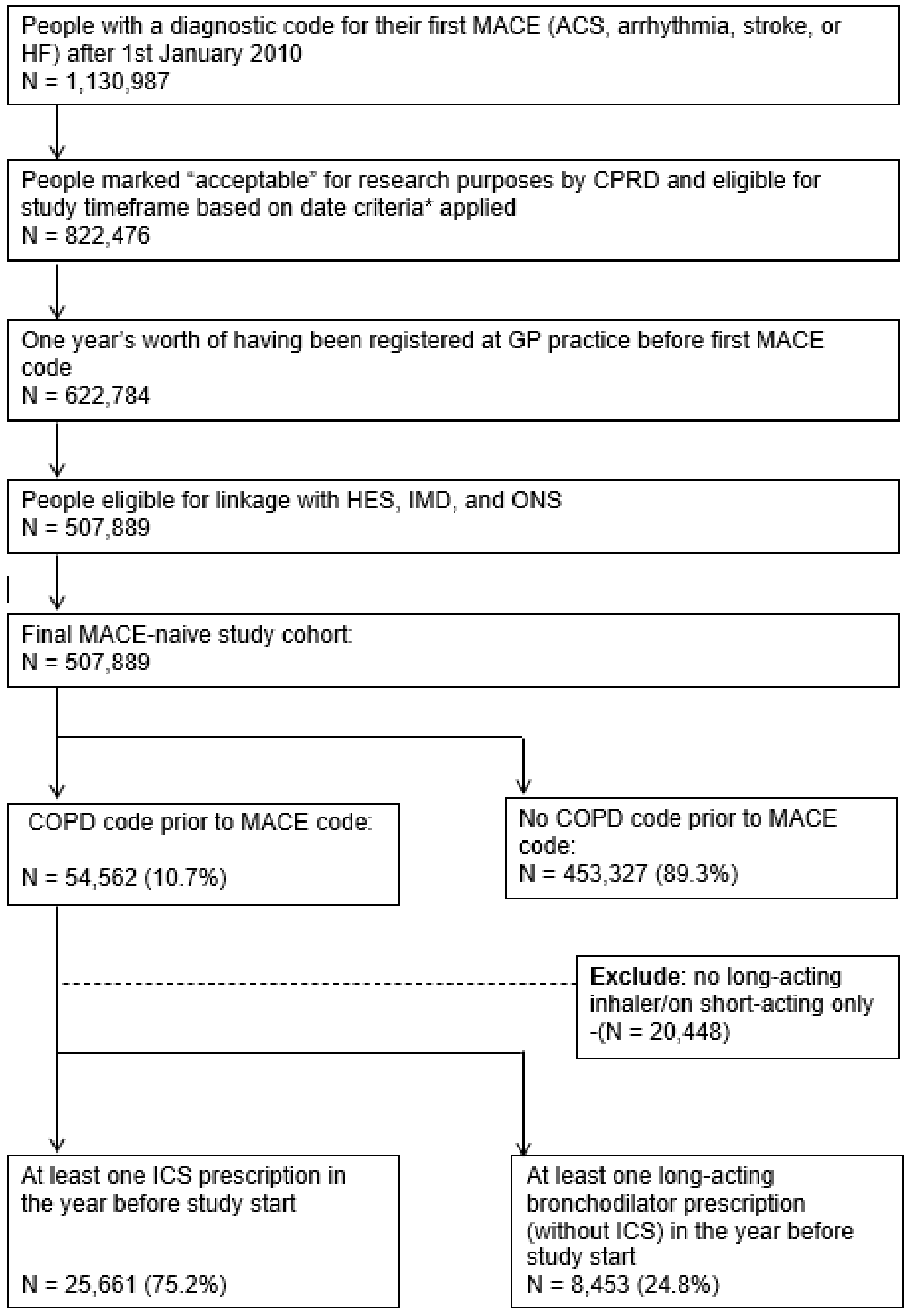

Figure E10 Inclusion and exclusion flow diagram for the MACE cohort
***Date criteria applied:** study start = latest of 1Jan2010, GP registration date + 1year, first MACE; study end = earliest of 29Mar2021, out-transfer (GP or CPRD), death date)
Abbreviations: CPRD (Clinical Practice Research Datalink), HES (Hospital Episode Statistics), IMD (Index of Multiple Deprivation), ONS (Office for National Statistics), GP (general practitioner), MACE (major adverse cardiovascular event = acute coronary syndrome, arrhythmias, heart failure, or ischemic stroke), COPD (chronic obstructive pulmonary disease), ICS (inhaled corticosteroids)

Table E8: COPD-specific baseline characteristics for MACE history population

Covariate relative to index (N (%) unless specified)	Total (N=507,889)	COPD diagnosis (N=54,562, 10.7%)	No COPD history (N=453,327, 89.3%)	Incident COPD diagnosis (N=904, 0.2%)	No COPD history vs incident (N=452,423, 99.8%)	At risk of COPD (absence of infection) (N=269,844, 59.5%)	No COPD history, nor at risk (vs at risk without infection) (N=183,483, 40.5%)	At risk of COPD (presence of infection) (N=3,142, 0.7%)	No COPD history, nor at risk (vs presence of infection) (N=450,185, 99.3%)
Study follow-up in years (median, IQR)	1.9 [0.5, 4.4]	1.5 [0.4, 3.6]	2.0 [0.5, 4.6]	1.7 [0.5, 4.0]	2.0 [0.5, 4.6]	2.0 [0.5, 4.6]	2.0 [0.5, 4.5]	1.5 [0.4, 3.5]	2.0 [0.5, 4.6]
All range (1 day to 12 years)									
**Age at entry (mean, SD)**	71.8 (13.1)	74.8 (10.0)	71.4 (13.4)	73.0 (10.4)	71.4 (13.4)	71.9 (13.1)	70.8 (13.7)	75.6 (12.5)	71.4 (13.4)
**Sex**									
Male	279,464(55.0)	30,748 (56.4)	248,716(54.9)	531 (58.7)	248,185(54.9)	158,424(58.7)	90,292 (49.2)	1,592 (50.7)	247,124(54.9)
Female	228,425(45.0)	23,814 (43.7)	204,611(45.1)	373 (41.3)	204,238(45.1)	111,420(41.3)	93,191 (50.8)	1,550 (49.3)	203,061(45.1)
**Smoking status**									
Never-smoker	82,733 (16.3)	1,687 (3.1)	81,046 (17.9)	31 (3.4)	81,015 (17.9)	--	81,046 (44.2)	--	81,046 (18.0)
Ex-smoker	324,255(63.8)	35,450 (65.0)	288,805(63.7)	516 (57.1)	288,289(63.8)	208,927(77.6)	79,878 (43.5)	2,442 (77.9)	286,363(63.7)
Current smoker	100,340(19.8)	17,419 (31.9)	82,921 (18.3)	357 (39.5)	82,564 (18.3)	60,384 (22.4)	22,537 (12.3)	695 (22.2)	82,226 (18.3)
**Index of Multiple Deprivation quintile**									
1 Least Deprived	110,793(21.8)	8,156 (15.0)	102,637(22.6)	135 (14.9)	102,502(22.7)	59,903 (22.2)	42,734 (23.3)	567 (18.1)	102,070(22.7)
2	110,295(21.7)	9,970 (18.3)	100,325(22.1)	172 (19.0)	100,153(22.1)	59,916 (22.2)	40,409 (22.0)	647 (20.6)	99,678 (22.1)
3	99,749 (19.6)	10,186 (18.7)	89,563 (19.8)	176 (19.5)	89,387 (19.8)	53,754 (19.9)	35,809 (19.5)	631 (20.1)	88,932 (19.8)
4	95,238 (18.8)	11,809 (21.6)	83,429 (18.4)	197 (21.8)	83,232 (18.4)	49,873 (18.5)	33,556 (18.3)	607 (19.3)	82,822 (18.4)
5 Most Deprived	91,346 (18.0)	14,406 (26.4)	76,940 (17.0)	223 (24.7)	76,717 (17.0)	46,125 (17.1)	30,815 (16.8)	689 (21.9)	76,251 (16.9)
Missing/Unknown	468 (0.1)	35 (0.1)	433 (0.1)	###	432 (0.1)	273 (0.1)	160 (0.1)	###	432 (0.1)
**Comorbidities***									
BMI (kg/m^2)									
Normal (18.5 to <25)	111,290(21.9)	15,652 (28.7)	95,638 (21.1)	223 (24.7)	95,415 (21.1)	56,326 (20.9)	39,312 (21.4)	634 (20.2)	95,004 (21.1)
Underweight (<18.5)	8,799 (1.7)	2,447 (4.5)	6,352 (1.4)	29 (3.2)	6,323 (1.4)	3,792 (1.4)	2,560 (1.4)	72 (2.3)	6,280 (1.4)
Overweight (25 to <30)	150,920(29.7)	16,893 (31.0)	134,027(29.6)	243 (26.9)	133,784(29.6)	79,880 (29.6)	54,147 (29.5)	894 (28.5)	133,133(29.6)
Obese (30+)	142,703(28.1)	17,277 (31.7)	125,426(27.7)	264 (29.2)	125,162(27.7)	71,988 (26.7)	53,438 (29.1)	1,033 (32.9)	124,393(27.6)
Missing/Unknown	94,177 (18.5)	2,293 (4.2)	91,884 (20.3)	145 (16.0)	91,739 (20.3)	57,858 (21.4)	34,026 (18.5)	509 (16.2)	91,375 (20.3)
Chronic Kidney Disease									
Yes	103,367(20.4)	12,882 (23.6)	90,485 (20.0)	198 (21.9)	90,287 (20.0)	55,487 (20.6)	34,998 (19.1)	926 (29.5)	89,559 (19.9)
No	312,399(61.5)	35,413 (64.9)	276,986(61.1)	552 (61.1)	276,434(61.1)	162,681(60.3)	114,305(62.3)	1,909 (60.8)	275,077(61.1)
Missing	92,123 (18.1)	6,267 (11.5)	85,856 (18.9)	154 (17.0)	85,702 (18.9)	51,676 (19.2)	34,180 (18.6)	307 (9.8)	85,549 (19.0)

Covariate relative to index (N (%) unless specified)	Total	COPD diagnosis	No COPD history	Incident COPD diagnosis	No COPD history vs incident	At risk of COPD (absence of infection)	No COPD history, nor at risk (vs at risk without infection)	At risk of COPD (presence of infection)	No COPD history, nor at risk (vs presence of infection)
Type II Diabetes	91,598 (18.0)	11,218 (20.6)	80,380 (17.7)	172 (19.0)	80,208 (17.7)	49,680 (18.4)	30,700 (16.7)	753 (24.0)	79,627 (17.7)
Hypertension	294,209(57.9)	33,733 (61.8)	260,476(57.5)	538 (59.5)	259,938(57.5)	157,032(58.2)	103,444(56.4)	2,051 (65.3)	258,425(57.4)
GORD	94,210 (18.6)	13,135 (24.1)	81,075 (17.9)	154 (17.0)	80,921 (17.9)	47,104 (17.5)	33,971 (18.5)	785 (25.0)	80,290 (17.8)
Asthma	150,911(29.7)	31,058 (56.9)	119,853(26.4)	274 (30.3)	119,579(26.4)	--	119,853(65.3)	--	119,853(26.6)
Depression	86,708 (17.1)	12,581 (23.1)	74,127 (16.4)	164 (18.1)	73,963 (16.4)	43,249 (16.0)	30,878 (16.8)	669 (21.3)	73,458 (16.3)
Anxiety	75,586 (14.9)	10,318 (18.9)	65,268 (14.4)	124 (13.7)	65,144 (14.4)	37,533 (13.9)	27,735 (15.1)	581 (18.5)	64,687 (14.4)
**Cardiovascular-related prescriptions****									
Any	387,009(76.2)	46,255 (84.8)	340,754(75.2)	708 (78.3)	340,046(75.2)	204,444(75.8)	136,310(74.3)	2,700 (85.9)	338,054(75.1)
Diuretics	168,167(33.1)	24,350 (44.6)	143,817(31.7)	344 (38.1)	143,473(31.7)	85,330 (31.6)	58,487 (31.9)	1,450 (46.2)	142,367(31.6)
Beta blockers	137,825(27.1)	11,632 (21.3)	126,193(27.8)	240 (26.6)	125,953(27.8)	79,633 (29.5)	46,560 (25.4)	1,088 (34.6)	125,105(27.8)
Hypertension, HF drugs	238,132(46.9)	27,978 (51.3)	210,154(46.4)	430 (47.6)	209,724(46.4)	126,892(47.0)	83,262 (45.4)	1,645 (52.4)	208,509(46.3)
Nitrates,CCBs,antianginals	201,296(39.6)	24,806 (45.5)	176,490(38.9)	390 (43.1)	176,100(38.9)	106,560(39.5)	69,930 (38.1)	1,433 (45.6)	175,057(38.9)
Antiplatelets	163,617(32.2)	21,592 (39.6)	142,025(31.3)	324 (35.8)	141,701(31.3)	87,532 (32.4)	54,493 (29.7)	1,259 (40.1)	140,766(31.3)
Statins	221,431(43.6)	28,541 (52.3)	192,890(42.6)	438 (48.5)	192,452(42.5)	118,096(43.8)	74,794 (40.8)	1,595 (50.8)	191,295(42.5)

Comorbidities defined as 'ever' in history except for BMI (nearest to study start within five years), cardiovascular medications (defined in the last two years), and CKD (nearest within past 2 years, as eGFR mL/min as a measure of kidney function, e.g., 40 mL/min eGFR = 40% kidney function)

** n(%) described for cardiovascular prescription categories may not be mutually exclusive, as they can be taken in combination elsewhere in the table (e.g., patient prescribed a diuretic and statins)
Too few to report (as per CPRD patient confidentiality policy)

Abbreviations: MACE = major adverse cardiovascular event (acute coronary syndrome; arrhythmias; heart failure; ischaemic stroke); COPD = chronic obstructive pulmonary disease; BMI = body mass index, CKD = chronic kidney disease; eGFR = estimated glomerular filtration rate; GORD = gastro-oesophageal reflux disease; HF = heart failure; CCB = calcium channel blocker

Table E9: ICS-specific baseline characteristics for MACE history population with COPD

Covariate relative to index (N (%) unless specified)	Total (N=34,114)	ICS (N=25,661, 75.2%)	Non-ICS (N=8,453, 24.8%)
Study follow-up in years (median, IQR) All range (1 day to 12 years)	1.5 [0.4, 3.4]	1.5 [0.4, 3.5]	1.5 [0.4, 3.2]
**Age at entry (mean, SD)**	74.6 (9.7)	74.6 (9.7)	74.9 (9.7)
**Sex**			
Male	19,251 (56.4)	14,251 (55.5)	5,000 (59.2)
Female	14,863 (43.6)	11,410 (44.5)	3,453 (40.9)
**Smoking status**			
Never-smoker	779 (2.3)	651 (2.5)	128 (1.5)
Ex-smoker	22,251 (65.2)	17,044 (66.4)	5,207 (61.6)
Current smoker	11,080 (32.5)	7,963 (31.0)	3,117 (36.9)
**Index of Multiple Deprivation quintile**			
1 Least Deprived	4,970 (14.6)	3,692 (14.4)	1,278 (15.1)
2	6,034 (17.7)	4,531 (17.7)	1,503 (17.8)
3	6,312 (18.5)	4,717 (18.4)	1,595 (18.9)
4	7,402 (21.7)	5,566 (21.7)	1,836 (21.7)
5 Most Deprived	9,374 (27.5)	7,138 (27.8)	2,236 (26.5)
Missing/Unknown	22 (0.1)	17 (0.1)	###
**Comorbidities***			
BMI (kg/m^2)			
Normal (18.5 to <25)	9,981 (29.3)	7,606 (29.6)	2,375 (28.1)
Underweight (<18.5)	1,697 (5.0)	1,354 (5.3)	343 (4.1)
Overweight (25 to <30)	10,322 (30.3)	7,642 (29.8)	2,680 (31.7)
Obese (30+)	11,036 (32.4)	8,249 (32.2)	2,787 (33.0)
Missing/Unknown	1,078 (3.2)	810 (3.2)	268 (3.2)
Chronic Kidney Disease			
Yes	7,758 (22.7)	5,664 (22.1)	2,094 (24.8)
No	22,593 (66.2)	17,150 (66.8)	5,443 (64.4)
Missing	3,763 (11.0)	2,847 (11.1)	916 (10.8)
Type II Diabetes	6,940 (20.3)	5,217 (20.3)	1,723 (20.4)
Hypertension	20,870 (61.2)	15,582 (60.7)	5,288 (62.6)
GORD	8,259 (24.2)	6,307 (24.6)	1,952 (23.1)
Asthma	20,338 (59.6)	16,939 (66.0)	3,399 (40.2)
Depression	8,145 (23.9)	6,225 (24.3)	1,920 (22.7)
Anxiety	6,679 (19.6)	5,081 (19.8)	1,598 (18.9)
**Cardiovascular-related prescriptions****			
Any	29,165 (85.5)	21,983 (85.7)	7,182 (85.0)
Diuretics	16,032 (47.0)	12,488 (48.7)	3,544 (41.9)
Beta blockers	6,869 (20.1)	4,729 (18.4)	2,140 (25.3)
Hypertension and HF drugs	17,505 (51.3)	13,100 (51.1)	4,405 (52.1)

Covariate relative to index (N (%) unless specified)	Total	ICS	Non-ICS
Nitrates, CCBs, Antianginals	15,503 (45.4)	11,664 (45.5)	3,839 (45.4)
Antiplatelets	13,509 (39.6)	10,069 (39.2)	3,440 (40.7)
Statins	18,112 (53.1)	13,451 (52.4)	4,661 (55.1)
**COPD characteristics**			
COPD exacerbations			
None	17,824 (52.3)	12,382 (48.3)	5,442 (64.4)
1 moderate, 0 severe	4,698 (13.8)	3,570 (13.9)	1,128 (13.3)
2 moderate, 0 severe	1,463 (4.3)	1,184 (4.6)	279 (3.3)
3+ moderate, 0 severe	859 (2.5)	732 (2.9)	127 (1.5)
Any moderate, 1 severe	6,436 (18.9)	5,251 (20.5)	1,185 (14.0)
Any moderate, 2+ severe	2,834 (8.3)	2,542 (9.9)	292 (3.5)
MRC dyspnoea group			
1	2,556 (7.5)	1,671 (6.5)	885 (10.5)
2	9,194 (27.0)	6,095 (23.8)	3,099 (36.7)
3	9,800 (28.7)	7,426 (28.9)	2,374 (28.1)
4	7,262 (1.3)	6,100 (23.8)	1,162 (13.8)
5	1,848 (5.4)	1,639 (6.4)	209 (2.5)
Missing	3,454 (10.1)	2,730 (10.6)	724 (8.6)
GOLD group			
1 (Mild)	5,223 (15.3)	3,444 (13.4)	1,779 (21.1)
2 (Moderate)	13,151 (38.6)	9,242 (36.0)	3,909 (46.2)
3 (Severe)	7,104 (20.8)	5,878 (22.9)	1,226 (14.5)
4 (Very severe)	1,877 (5.5)	1,702 (6.6)	175 (2.1)
Missing	6,759 (19.8)	5,395 (21.0)	1,364 (16.1)
Short-acting medications			
None	3,453 (10.1)	1,849 (7.2)	1,604 (19.0)
SABA	29,231 (85.7)	22,674 (88.4)	6,557 (77.6)
SAMA	170 (0.5)	116 (0.5)	54 (0.6)
SABA-SAMA	1,260 (3.7)	1,022 (4.0)	238 (2.8)

ICS = ICS monotherapy, ICS-LABA, ICS-LAMA, or ICS-LABA-LAMA (triple therapy)
Non-ICS = any long-acting bronchodilator, i.e., LABA, LAMA, or LABA-LAMA

Comorbidities defined as 'ever' in history, except BMI (defined as nearest to study start within five years), cardiovascular medications (defined within the 2 years before study start), CKD (defined as nearest to study start within 2 years, as a measure of kidney function, e.g., 40 mL/min eGFR = 40% kidney function), MRC dyspnoea group (defined within two years before and three months after study start), COPD exacerbations (defined in the year before study start), and short-acting inhalers (defined in the year before study start)

** n(%) described for cardiovascular prescription categories may not be mutually exclusive, as they can be taken in combination elsewhere in the table (e.g., patient prescribed a diuretic and statins)
Too few to report (as per CPRD patient confidentiality policy)

Abbreviations: ICS = inhaled corticosteroids; LABA = long-acting beta agonist; LAMA = long-acting muscarinic antagonist; MACE = major adverse cardiovascular event (acute coronary syndrome; arrhythmias; heart failure; ischaemic stroke); COPD = chronic obstructive pulmonary disease; BMI = body mass index; CKD = chronic kidney disease; GORD = gastro-oesophageal reflux disease; HF = heart failure; CCB = calcium channel blocker; MRC = Medical Research Council; GOLD = Global Initiative for Chronic Obstructive Lung Disease; SABA = short-acting beta agonist; SAMA = short-acting muscarinic antagonist

Age65+

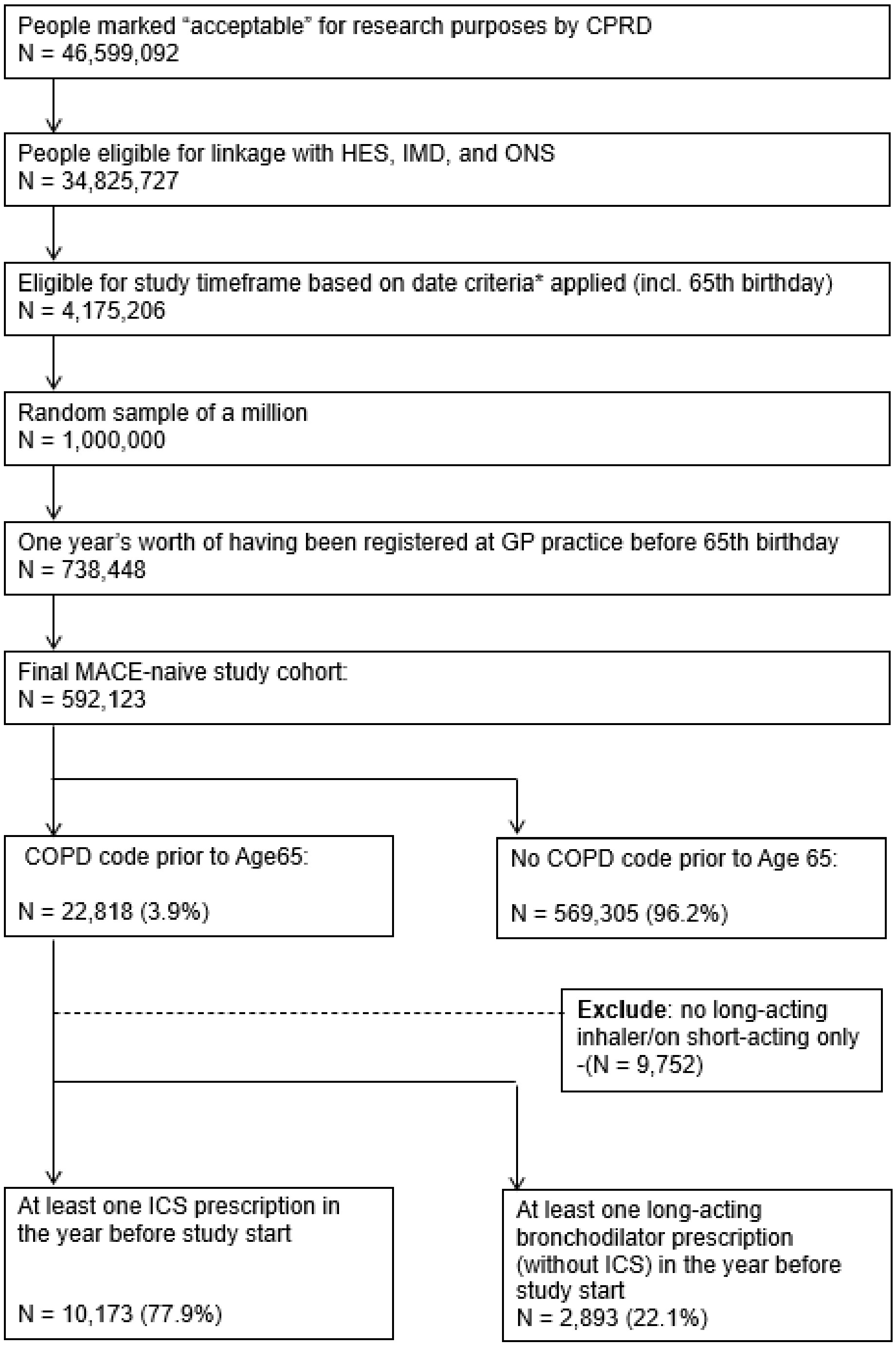

Figure E11: Inclusion and exclusion flow diagram for the Age65+ cohort
***Date criteria applied:** study start = latest of 1Jan2010, GP registration date + 1year, 65th birthday; study end = earliest of 29Mar2021, out-transfer (GP or CPRD), death date)
Abbreviations: CPRD (Clinical Practice Research Datalink), HES (Hospital Episode Statistics), IMD (Index of Multiple Deprivation), ONS (Office for National Statistics), GP (general practitioner), MACE (major adverse cardiovascular event = acute coronary syndrome, arrhythmias, heart failure, or ischemic stroke), COPD (chronic obstructive pulmonary disease), ICS (inhaled corticosteroids)

Table E10: COPD-specific baseline characteristics for Age65+ population

Covariate relative to index (N (%) unless specified)	Total (N=592,123)	COPD diagnosis (N=22,818, 3.9%)	No COPD history (N=569,305, 96.2%)	At risk of COPD (absence of infection) (N=324,612, 57.0%)	No COPD history, nor at risk (vs at risk without infection) (N=244,693, 43.0%)	At risk of COPD (presence of infection) (N=1,852, 0.3%)	No COPD history, nor at risk (vs presence of infection) (N=567,453, 99.7%)
Study follow-up in years (median, IQR)	5.9 [2.7, 9.9]	4.7 [1.9, 7.7]	6.0 [2.7, 10.0]	6.2 [2.7, 10.1]	5.8 [2.7, 9.9]	5.0 [2.3, 9.0]	6.0 [2.7, 10.0]
All range (1 day to 12 years)							
**Age at entry (mean, SD)**	70.4 (7.4)	66.9 (4.0)	70.5 (7.4)	70.6 (7.4)	70.5 (7.5)	72.3 (8.6)	70.5 (7.4)
**Sex**							
Male	264,841 (44.7)	11,544 (50.6)	253,297 (44.5)	155,620 (47.9)	97,677 (39.9)	787 (42.5)	252,510 (44.5)
Female	327,282 (55.3)	11,274 (49.4)	316,008 (55.5)	168,992 (52.1)	147,016 (60.1)	1,065 (57.5)	314,943 (55.5)
**Smoking status**							
Never-smoker	129,471 (21.9)	652 (2.9)	128,819 (22.6)	--	128,819 (52.7)	--	128,819 (22.7)
Ex-smoker	349,840 (59.1)	12,244 (53.7)	337,596 (59.3)	247,720 (76.3)	89,876 (36.7)	1,298 (70.1)	336,298 (59.3)
Current smoker	112,812 (19.1)	9,922 (43.5)	102,890 (18.1)	76,892 (23.4)	25,998 (10.6)	554 (29.9)	102,336 (18.0)
**Index of Multiple Deprivation quintile**							
1 Least Deprived	142,514 (24.1)	2,993 (13.1)	139,521 (24.5)	78,400 (24.2)	61,121 (25.0)	377 (20.4)	139,144 (24.5)
2	135,938 (23.0)	3,850 (16.8)	132,088 (23.2)	75,545 (23.3)	56,543 (23.1)	378 (20.4)	131,710 (23.2)
3	117,378 (19.8)	4,301 (18.9)	113,077 (19.9)	64,788 (20.0)	48,289 (19.7)	368 (19.9)	112,709 (19.9)
4	104,853 (17.7)	5,111 (22.4)	99,742 (17.5)	57,025 (17.6)	42,717 (17.5)	360 (19.4)	99,382 (17.5)
5 Most Deprived	89,508 (15.1)	6,535 (28.6)	82,973 (14.6)	47,833 (14.7)	35,140 (14.4)	366 (19.8)	82,607 (14.6)
Missing/Unknown	1,932 (0.3)	28 (0.1)	1,904 (0.3)	1,021 (0.3)	883 (0.4)	###	1,901 (0.3)
**Comorbidities***							
BMI (kg/m^2)							
Normal (18.5 to <25)	132,141 (22.3)	6,660 (29.2)	125,481 (22.0)	70,817 (21.8)	54,664 (22.3)	374 (20.2)	125,107 (22.1)
Underweight (<18.5)	8,066 (1.4)	974 (4.3)	7,092 (1.3)	4,150 (1.3)	2,942 (1.2)	56 (3.0)	7,036 (1.2)
Overweight (25 to <30)	171,826 (29.0)	7,251 (31.8)	164,575 (28.9)	94,008 (29.0)	70,567 (28.8)	579 (31.3)	163,996 (28.9)
Obese (30+)	126,802 (21.4)	6,686 (29.3)	120,116 (21.1)	67,447 (20.8)	52,669 (21.5)	490 (26.5)	119,626 (21.1)
Missing/Unknown	153,288 (25.9)	1,247 (5.5)	152,041 (26.7)	88,190 (27.2)	63,851 (26.1)	353 (19.1)	151,688 (26.7)
Chronic Kidney Disease							
Yes	68,240 (11.5)	1,722 (7.6)	66,518 (11.7)	39,629 (12.2)	26,889 (11.0)	319 (17.2)	66,199 (11.7)
No	320,270 (54.1)	15,216 (66.7)	305,054 (53.6)	175,464 (54.1)	129,590 (53.0)	1,145 (61.8)	303,909 (53.6)
Missing	203,613 (34.4)	5,880 (25.8)	197,733 (34.7)	109,519 (33.7)	88,214 (36.1)	388 (21.0)	197,345 (34.8)

Covariate relative to index (N (%) unless specified)	Total	COPD diagnosis	No COPD history	At risk of COPD (absence of infection)	No COPD history, nor at risk (vs at risk without infection)	At risk of COPD (presence of infection)	No COPD history, nor at risk (vs presence of infection)
Type II Diabetes	68,725 (11.6)	3,077 (13.5)	65,648 (11.5)	40,288 (12.4)	25,360 (10.4)	320 (17.3)	65,328 (11.5)
Hypertension	263,615 (44.5)	10,015 (43.9)	253,600 (44.6)	149,750 (46.1)	103,850 (42.4)	950 (51.3)	252,650 (44.5)
GORD	82,216 (13.9)	4,477 (19.6)	77,739 (13.7)	44,007 (13.6)	33,732 (13.8)	380 (20.5)	77,359 (13.6)
Asthma	153,450 (25.9)	13,820 (60.6)	139,630 (24.5)	--	139,630 (57.1)	--	139,630 (24.6)
Depression	82,703 (14.0)	5,790 (25.4)	76,913 (13.5)	44,942 (13.8)	31,971 (13.1)	389 (21.0)	76,524 (13.5)
Anxiety	70,791 (12.0)	4,267 (18.7)	66,524 (11.7)	38,487 (11.9)	28,037 (11.5)	321 (17.3)	66,203 (11.7)
**Cardiovascular-related prescriptions****							
Any	341,352 (57.7)	14,659 (64.2)	326,693 (57.4)	192,623 (59.3)	134,070 (54.8)	1,303 (70.4)	325,390 (57.3)
Diuretics	138,936 (23.5)	5,786 (25.4)	133,150 (23.4)	77,737 (24.0)	55,413 (22.7)	615 (33.2)	132,535 (23.4)
Beta blockers	78,401 (13.2)	1,710 (7.5)	76,691 (13.5)	49,555 (15.3)	27,136 (11.1)	351 (19.0)	76,340 (13.5)
Hypertension and HF drugs	196,200 (33.1)	8,007 (35.1)	188,193 (33.1)	112,202 (34.6)	75,991 (31.1)	770 (41.6)	187,423 (33.0)
Nitrates, CCBs, Antianginals	150,838 (25.5)	6,723 (29.5)	144,115 (25.3)	85,835 (26.4)	58,280 (23.8)	591 (31.9)	143,524 (25.3)
Antiplatelets	113,623 (19.2)	4,946 (21.7)	108,677 (19.1)	66,937 (20.6)	41,740 (17.1)	528 (28.5)	108,149 (19.1)
Statins	194,524 (32.9)	9,252 (40.6)	185,272 (32.5)	111,392 (34.3)	73,880 (30.2)	746 (40.3)	184,526 (32.5)

Comorbidities defined as 'ever' in history except for BMI (nearest to study start within five years), cardiovascular medications (defined in the last two years), and CKD (nearest within past 2 years, as eGFR mL/min as a measure of kidney function, e.g., 40 mL/min eGFR = 40% kidney function)

** n(%) described for cardiovascular prescription categories may not be mutually exclusive, as they can be taken in combination elsewhere in the table (e.g., patient prescribed a diuretic and statins)
Too few to report (as per CPRD patient confidentiality policy)

Abbreviations: MACE = major adverse cardiovascular event (acute coronary syndrome; arrhythmias; heart failure; ischaemic stroke); COPD = chronic obstructive pulmonary disease; BMI = body mass index, CKD = chronic kidney disease; eGFR = estimated glomerular filtration rate; GORD = gastro-oesophageal reflux disease; HF = heart failure; CCB = calcium channel blocker

Table E11: ICS-specific baseline characteristics for Age65+ population with COPD

Covariate relative to index (N (%) unless specified)	Total (N=13,066)	ICS (N=10,173, 77.9%)	Non-ICS (N=2,893, 22.1%)
Study follow-up in years (median, IQR)	4.3 [1.7, 7.7]	4.6 [1.9, 7.7]	3.7 [1.7, 6.7]
All range (1 day to 12 years)			
**Age at entry (mean, SD)**	66.9 (4.0)	67.2 (4.2)	66.1 (3.1)
**Sex**			
Male	6,540 (50.0)	4,999 (49.1)	1,541 (53.3)
Female	6,526 (50.0)	5,174 (50.9)	1,352 (46.7)
**Smoking status**			
Never-smoker	260 (2.0)	229 (2.3)	31 (1.1)
Ex-smoker	7,030 (53.8)	5,642 (55.5)	1,388 (48.0)
Current smoker	5,776 (44.2)	4,302 (42.3)	1,474 (51.0)
**Index of Multiple Deprivation quintile**			
1 Least Deprived	1,593 (12.2)	1,248 (12.3)	345 (11.9)
2	2,086 (16.0)	1,632 (16.0)	454 (15.7)
3	2,346 (18.0)	1,841 (18.1)	505 (17.5)
4	2,985 (22.9)	2,321 (22.8)	664 (23.0)
5 Most Deprived	4,045 (31.0)	3,124 (30.7)	921 (31.8)
Missing/Unknown	11 (0.1)	###	###
**Comorbidities***			
BMI (kg/m^2)			
Normal (18.5 to <25)	3,963 (30.3)	3,082 (30.3)	881 (30.5)
Underweight (<18.5)	632 (4.8)	505 (5.0)	127 (4.4)
Overweight (25 to <30)	4,076 (31.2)	3,143 (30.9)	933 (32.3)
Obese (30+)	3,894 (29.8)	3,047 (30.0)	847 (29.3)
Missing/Unknown	501 (3.8)	396 (3.9)	105 (3.6)
Chronic Kidney Disease			
Yes	978 (7.5)	780 (7.7)	198 (6.8)
No	8,873 (67.9)	6,852 (67.4)	2,021 (69.9)
Missing	3,215 (24.6)	2,541 (25.0)	674 (23.3)
Type II Diabetes	1,745 (13.4)	1,346 (13.2)	399 (13.8)
Hypertension	5,795 (44.4)	4,513 (44.4)	1,282 (44.3)
GORD	2,676 (20.5)	2,104 (20.7)	527 (19.8)
Asthma	8,462 (64.8)	7,179 (70.6)	1,283 (44.4)
Depression	3,496 (26.8)	2,663 (26.2)	833 (28.8)
Anxiety	2,545 (19.5)	1,952 (19.2)	593 (20.5)
**Cardiovascular-related prescriptions****			
Any	8,621 (66.0)	6,735 (66.2)	1,886 (65.2)
Diuretics	3,575 (27.4)	2,914 (28.6)	661 (22.9)
Beta blockers	905 (6.9)	607 (6.0)	298 (10.3)

Covariate relative to index (N (%) unless specified)	Total	ICS	Non-ICS
Hypertension and heart failure drugs	4,664 (35.7)	3,653 (35.9)	1,011 (35.0)
Nitrates, CCBs, other antianginals	3,991 (30.5)	3,134 (30.8)	857 (29.6)
Antiplatelets	2,941 (22.5)	2,319 (22.8)	622 (21.5)
Statins	5,409 (41.4)	4,155 (40.8)	1,254 (43.4)
**COPD characteristics**			
COPD exacerbations			
None	8,838 (67.6)	6,634 (65.2)	2,204 (76.2)
1 moderate, 0 severe	2,043 (15.6)	1,640 (16.1)	403 (13.9)
2 moderate, 0 severe	692 (5.3)	575 (5.7)	117 (4.0)
3+ moderate, 0 severe	419 (3.2)	369 (3.6)	50 (1.7)
Any moderate, 1 severe	781 (6.0)	681 (6.7)	100 (3.5)
Any moderate, 2+ severe	293 (2.2)	274 (2.7)	19 (0.7)
MRC dyspnoea group			
1	1,609 (12.3)	1,107 (10.9)	502 (17.4)
2	4,231 (32.4)	3,039 (29.9)	1,192 (41.2)
3	3,413 (26.1)	2,711 (26.7)	702 (24.3)
4	1,957 (15.0)	1,713 (16.8)	244 (8.4)
5	464 (3.6)	412 (4.1)	52 (1.8)
Missing/Unknown	1,392 (10.7)	1,191 (11.7)	201 (7.0)
GOLD group			
1 (Mild)	1,971 (15.1)	1,369 (13.5)	602 (20.8)
2 (Moderate)	5,203 (39.8)	3,782 (37.2)	1,421 (49.1)
3 (Severe)	2,931 (22.4)	2,481 (24.4)	450 (15.6)
4 (Very severe)	891 (6.8)	810 (8.0)	81 (2.8)
Missing	2,070 (15.8)	1,731 (17.0)	339 (11.7)
Short-acting medications			
None	1,264 (9.7)	754 (7.4)	510 (17.6)
SABA	10,870 (83.2)	8,660 (85.1)	2,210 (76.4)
SAMA	99 (0.8)	74 (0.7)	25 (0.9)
SABA-SAMA	833 (6.4)	685 (6.7)	148 (5.1)

ICS = ICS monotherapy, ICS-LABA, ICS-LAMA, or ICS-LABA-LAMA (triple therapy)
Non-ICS = any long-acting bronchodilator, i.e., LABA, LAMA, or LABA-LAMA

Comorbidities defined as 'ever' in history, except BMI (defined as nearest to study start within five years), cardiovascular medications (defined within the 2 years before study start), CKD (defined as nearest to study start within 2 years, as a measure of kidney function, e.g., 40 mL/min eGFR = 40% kidney function), MRC dyspnoea group (defined within two years before and three months after study start), COPD exacerbations (defined in the year before study start), and short-acting inhalers (defined in the year before study start)

** n(%) described for cardiovascular prescription categories may not be mutually exclusive, as they can be taken in combination elsewhere in the table (e.g., patient prescribed a diuretic and statins)
Too few to report (as per CPRD patient confidentiality policy)

Abbreviations: ICS = inhaled corticosteroids; LABA = long-acting beta agonist; LAMA = long-acting muscarinic antagonist; MACE = major adverse cardiovascular event (acute coronary syndrome; arrhythmias; heart failure; ischaemic stroke); COPD = chronic obstructive pulmonary disease; BMI = body mass index; CKD = chronic kidney disease; GORD = gastro-oesophageal reflux disease; HF = heart failure; CCB = calcium channel blocker; MRC = Medical Research Council; GOLD = Global Initiative for Chronic Obstructive Lung Disease; SABA = short-acting beta agonist; SAMA = short-acting muscarinic antagonist

KAPLAN MEIER (KM) PLOTS: COPD EXPOSURE

Chronic Kidney Disease (CKD)

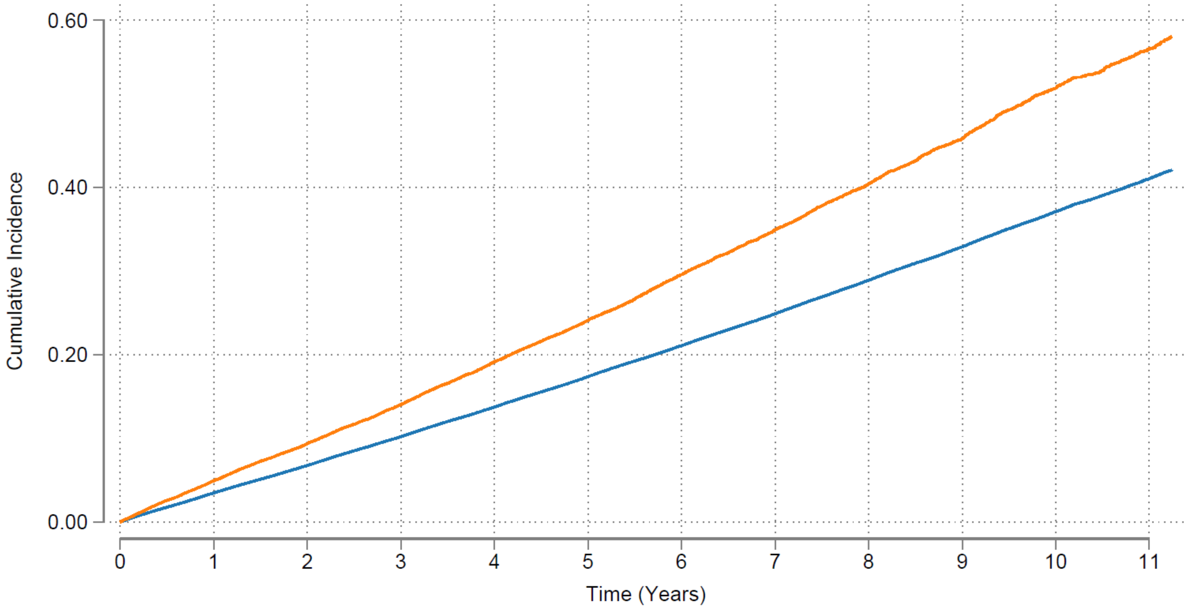

Number at Risk												
No COPD history	531998	463200	401539	344974	294550	250865	213332	179931	150128	123757	99088	76758
COPD history	41628	34036	27692	22134	17412	13617	10582	8131	6126	4559	3216	2213

Figure E12: KM plot for pre-existing COPD versus no COPD analysis for CKD cohort

Type-II Diabetes Mellitus (T2DM)

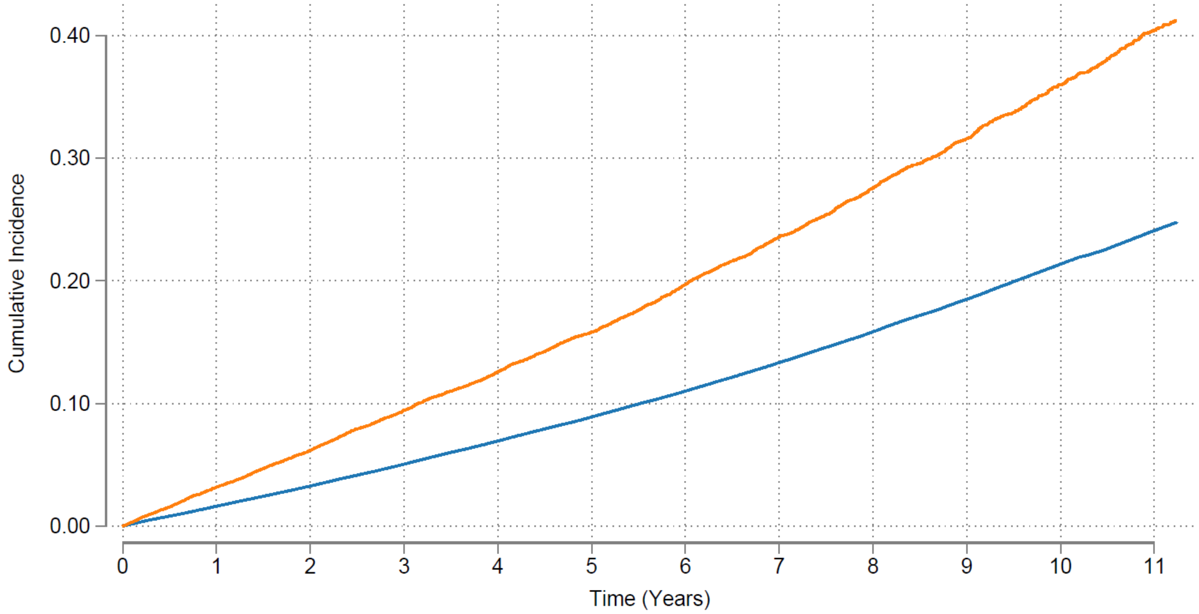

Number at Risk												
No COPD history	620148	557630	496254	439911	389735	343138	299160	260482	223899	189653	157692	130154
COPD history	29358	25034	20892	17263	14159	11489	8981	7079	5358	4063	2966	2068

Figure E13: KM plot for pre-existing COPD versus no COPD analysis for T2DM cohort

Obesity

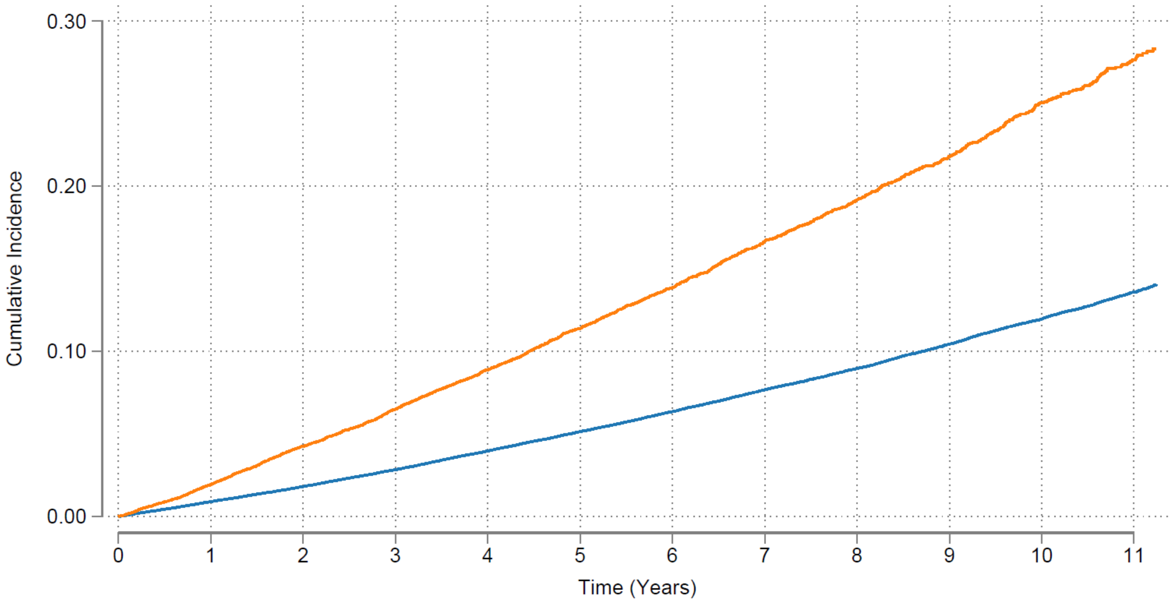

Number at Risk												
No COPD history	201912	183207	156209	133960	114958	97468	82053	69551	56158	45243	34075	22005
COPD history	23361	21423	17780	14584	11680	9166	7206	5672	4441	3359	2218	1033

No COPD history   COPD history

Figure E14: KM plot for pre-existing COPD versus no COPD analysis for Obesity cohort

MACE history

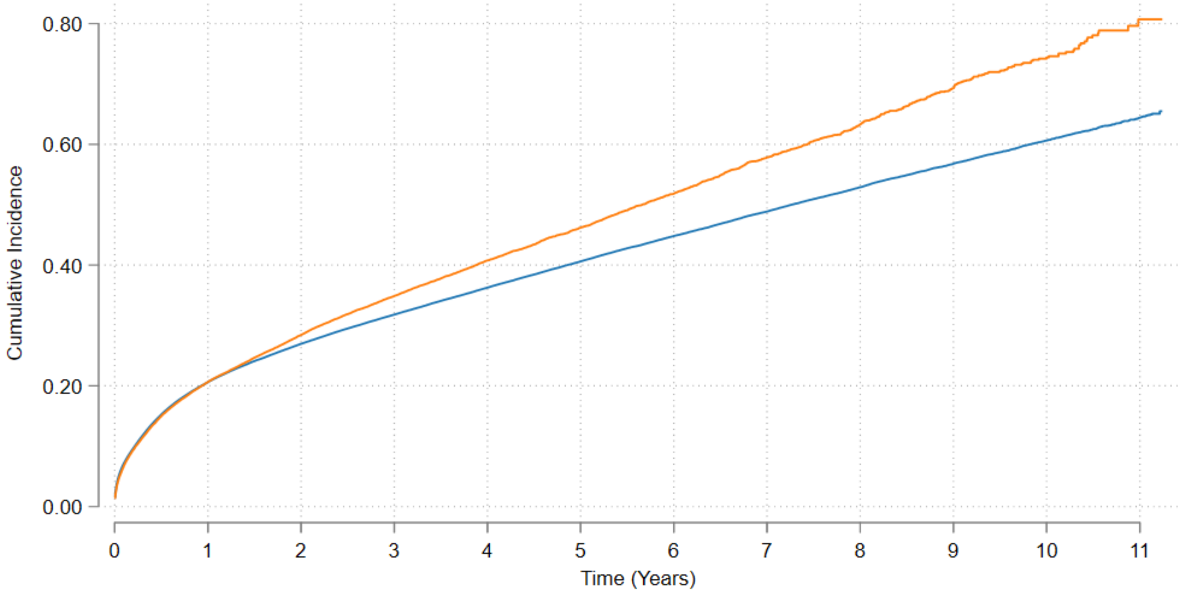

Number at Risk												
No COPD history	453327	296309	226308	174102	132433	99321	72627	50903	34196	20512	9878	1788
COPD history	54562	33465	23733	16865	11891	8308	5604	3609	2162	1179	501	86

No COPD history   COPD history

Figure E15: KM plot for pre-existing COPD versus no COPD analysis for MACE history cohort

Age65+

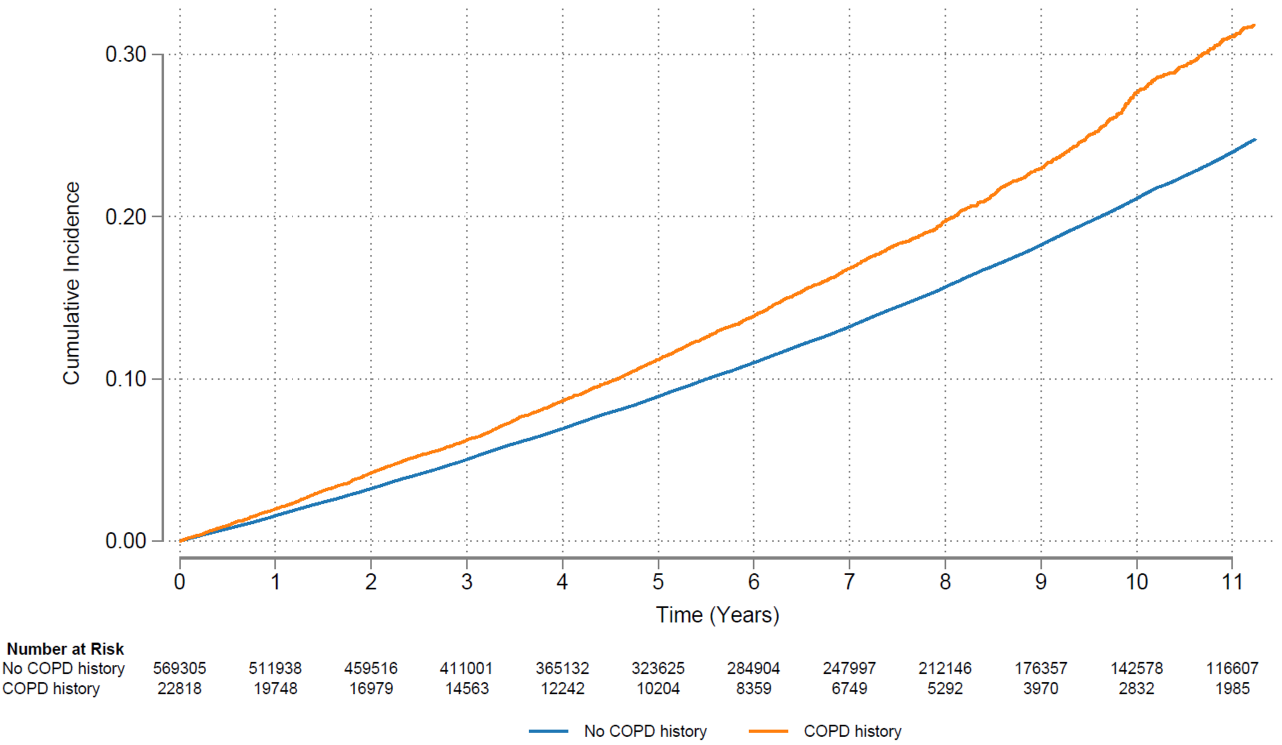

Figure E16: KM plot for pre-existing COPD versus no COPD analysis for Age65+ cohort

SENSITIVITY ANALYSIS RESULTS

Pre-existing COPD versus no COPD (Exposure 1a)

Table E12: Relationship between pre-existing COPD and subsequent MACE amongst six populations at elevated risk of MACE

Cohort	Metric	Outcome											
		MACE		***ACS***		***Arrhythmia***		***Ischaemic stroke***		***Heart failure***		***CV-specific death***	
		COPD	*No COPD*	*COPD*	*No COPD*	*COPD*	*No COPD*	*COPD*	*No COPD*	*COPD*	*No COPD*	*COPD*	*No COPD*
**CKD**	*Crude N*	41,628	531,998	60,923	693,483	56,416	651,788	67,433	729,520	64,768	740,144	41,628	531,998
	Crude N(%) outcome	8,390 (20.2)	102,588 (19.3)	2,581 (4.2)	27,742 (4.0)	2,787 (4.9)	32,292 (5.0)	2,423 (3.6)	33,228 (4.6)	4,413 (6.8)	43,300 (5.9)	3,689 (8.9)	44,960 (8.5)
	Crude HR (95%CI)	**1.39 (1.36, 1.42)**		**1.40 (1.35, 1.46)**		**1.35 (1.29, 1.40)**		**1.06 (1.02, 1.10)**		**1.58 (1.53, 1.63)**		**1.40 (1.36, 1.45)**	
	aHR (95%CI)	**1.29 (1.26, 1.32)**		**1.22 (1.17, 1.28)**		**1.25 (1.20, 1.30)**		1.06 (1.01, 1.10)		**1.42 (1.37, 1.47)**		**1.35 (1.31, 1.40)**	
	Sens: -BMI	**1.29 (1.26, 1.32)**		**1.23 (1.18, 1.28)**		**1.25 (1.20, 1.31)**		1.05 (1.01, 1.10)		**1.44 (1.39, 1.48)**		**1.36 (1.31, 1.40)**	
**T2DM**	*Crude N*	29,358	620,148	37,391	699,601	37,171	704,760	41,280	734,019	40,877	745,276	29,358	620,148
	Crude N(%) outcome	4,497 (15.3)	76,142 (12.3)	1,467 (3.9)	24,976 (3.6)	1,537 (4.1)	24,069 (3.4)	1,256 (3.0)	22,263 (3.0)	2,159 (5.3)	28,307 (3.8)	1,575 (5.4)	25,606 (4.1)
	Crude HR (95%CI)	**1.75 (1.70, 1.81)**		**1.53 (1.45, 1.61)**		**1.71 (1.63, 1.80)**		**1.42 (1.35, 1.51)**		**2.03 (1.94, 2.12)**		**1.84 (1.74, 1.93)**	
	aHR (95%CI)	**1.30 (1.26, 1.35)**		**1.20 (1.13, 1.27)**		**1.31 (1.24, 1.39)**		1.07 (1.01, 1.14)		**1.44 (1.38, 1.51)**		**1.36 (1.28, 1.43)**	
	Sens: -CKD	**1.28 (1.24, 1.32)**		**1.19 (1.13, 1.26)**		**1.29 (1.22, 1.36)**		1.06 (1.00, 1.13)		**1.42 (1.36, 1.49)**		**1.32 (1.25, 1.40)**	
	Sens: -BMI	**1.32 (1.27, 1.36)**		**1.19 (1.13, 1.26)**		**1.34 (1.27, 1.41)**		1.08 (1.01, 1.14)		**1.48 (1.41, 1.55)**		**1.36 (1.29, 1.44)**	
**Obesity**	*Crude N*	23,631	201,912	27,464	218,072	27,315	216,036	29,479	224,131	29,118	225,174	23,361	201,912
	Crude N(%) outcome	2,566 (11.0)	12,199 (6.0)	797 (2.9)	3,627 (1.7)	982 (3.6)	4,537 (2.1)	537 (1.8)	2,983 (1.3)	1,207 (4.2)	3,844 (1.7)	669 (2.9)	3,091 (1.5)
	Crude HR (95%CI)	**2.15 (2.06, 2.24)**		**2.04 (1.89, 2.21)**		**2.02 (1.89, 2.17)**		**1.63 (1.49, 1.79)**		**2.91 (2.72, 3.10)**		**2.18 (2.01, 2.37)**	
	aHR (95%CI)	**1.41 (1.34, 1.48)**		**1.40 (1.28, 1.53)**		**1.33 (1.22, 1.44)**		1.03 (0.92, 1.14)		**1.82 (1.69, 1.96)**		**1.31 (1.19, 1.45)**	
	Sens: -CKD	**1.40 (1.33, 1.46)**		**1.36 (1.25, 1.48)**		**1.35 (1.25, 1.45)**		1.04 (0.95, 1.15)		**1.80 (1.68, 1.93)**		**1.31 (1.20, 1.43)**	

Cohort	Metric	*MACE*		*ACS*		*Arrhythmia*		*Ischaemic stroke*		*Heart failure*		*CV-specific death*	
		COPD	*No COPD*	*COPD*	*No COPD*	*COPD*	*No COPD*	*COPD*	*No COPD*	*COPD*	*No COPD*	*COPD*	*No COPD*
**Age65+**	*Crude N*	22,818	569,305	25,925	634,889	26,456	625,413	27,451	652,635	27,738	665,535	22,818	569,305
	Crude N(%) outcome	2,866 (12.7)	71,569 (12.6)	861 (3.3)	18,635 (2.9)	962 (3.6)	23,821 (3.8)	687 (2.5)	20,468 (5.6)	870 (3.1)	20,370 (3.1)	908 (4.0)	25.921 (4.6)
	Crude HR (95%CI)	**1.27 (1.22, 1.32)**		**1.40 (1.31, 1.50)**		**1.22 (1.15, 1.30)**		1.01 (0.93, 1.09)		**1.33 (1.24, 1.43)**		**1.11 (1.04, 1.19)**	
	aHR (95%CI)	**1.59 (1.52, 1.66)**		**1.43 (1.32, 1.56)**		**1.37 (1.27, 1.48)**		**1.33 (1.22, 1.46)**		**1.81 (1.68, 1.96)**		**1.87 (1.73, 2.03)**	
	Sens: -CKD	**1.61 (1.54, 1.68)**		**1.47 (1.36, 1.58)**		**1.39 (1.30, 1.49)**		**1.38 (1.27, 1.50)**		**1.79 (1.67, 1.93)**		**1.85 (1.72, 1.98)**	
	Sens: -BMI	**1.60 (1.54, 1.67)**		**1.44 (1.33, 1.56)**		**1.37 (1.27, 1.48)**		**1.34 (1.23, 1.47)**		**1.84 (1.68, 1.96)**		**1.92 (1.78, 2.08)**	
**MACE history**	*Crude N*	54,562	453,327	54,562	453,327	54,562	453,327	54,562	453,327	54,562	453,327	54,562	453,327
	Crude N(%) outcome	15,112 (27.7)	126,419 (27.9)	3,333 (6.1)	25,951 (5.7)	6,222 (11.4)	61,487 (13.6)	2,413 (4.4)	22,657 (5.0)	5,222 (9.6)	32,040 (7.1)	887 (1.6)	6,328 (1.4)
	Crude HR (95%CI)	**1.09 (1.07, 1.11)**		**1.19 (1.14, 1.23)**		0.91 (0.89, 0.94)		1.00 (0.96, 1.05)		**1.58 (1.54, 1.63)**		**1.35 (1.26, 1.45)**	
	aHR (95%CI)	**1.04 (1.02, 1.06)**		**1.06 (1.02, 1.10)**		0.99 (0.96, 1.02)		0.91 (0.87, 0.96)		**1.26 (1.22, 1.30)**		1.11 (1.02, 1.20)	
	Sens: -CKD	**1.03 (1.01, 1.05)**		1.04 (1.00, 1.08)		0.98 (0.95, 1.01)		0.90 (0.86, 0.95)		**1.26 (1.22, 1.30)**		1.10 (1.02, 1.19)	
	Sens: -BMI	**1.04 (1.02, 1.06)**		**1.07 (1.02, 1.11)**		0.99 (0.96, 1.02)		0.90 (0.86, 0.95)		**1.27 (1.23, 1.32)**		**1.11 (1.03, 1.20)**	
**QRISK >10%**	*Crude N*	72,682	1,194,978	72,682	1,194,978	72,682	1,194,978	72,682	1,194,978	72,682	1,194,978	72,682	1,194,978
	Crude N(%) outcome	11,950 (16.4)	184,898 (15.5)	1,459 (2.0)	21,463 (1.8)	1.917 (2.6)	32,555 (2.7)	1,308 (1.8)	24,967 (2.1)	2,280 (3.1)	30,756 (2.6)	3,592 (4.9)	53,715 (4.5)
	Crude HR (95%CI)	**1.43 (1.41, 1.46)**		1.49 (1.41, 1.57)		1.34 (1.27, 1.40)		1.17 (1.10, 1.23)		1.73 (1.66, 1.81)		1.43 (1.38, 1.48)	
	aHR (95%CI)	**1.33 (1.30, 1.35)**		**1.30 (1.23, 1.38)**		**1.28 (1.22, 1.34)**		**1.16 (1.09, 1.23)**		**1.51 (1.44, 1.58)**		**1.39 (1.34, 1.45)**	
	Sens: -CKD	**1.33 (1.30, 1.35)**		1.31 (1.23, 1.38)		1.27 (1.21, 1.34)		1.15 (1.08, 1.22)		1.52 (1.45, 1.59)		1.41 (1.36, 1.46)	
	Sens: -BMI	**1.34 (1.31, 1.36)**		1.31 (1.23, 1.38)		1.29 (1.23, 1.36)		1.16 (1.09, 1.23)		1.54 (1.47, 1.62)		1.40 (1.35, 1.46)	

COPD: A COPD diagnostic code at any time before cohort-specific disease code (i.e., CKD, T2DM, Obesity, Age65+, MACE and QRISK calculation date)
Bold indicates positive statistical significance; red indicates negative statistical significance
Green model = main model presented in manuscript; adjusted for age, sex, socioeconomic deprivation, smoking status, hypertension, asthma, GORD, depression, anxiety, CVD medications, T2DM (except T2DM cohort), BMI (except obesity cohort), CKD (except CKD cohort) and cardiovascular history (MACE-subtypes only)
Sens: -VARIABLE = sensitivity analysis citing the covariate removed from the analysis
Abbreviations: COPD = chronic obstructive pulmonary disease; MACE = major adverse cardiovascular event; CKD = chronic kidney disease; T2Dm = Type-II diabetes mellitus; ACS = acute coronary syndrome; HR = hazard ratio; aHR = adjusted hazard ratio, BMI = body mass index

Descriptive characteristics: QRISK>10%

Table E13: COPD-specific baseline characteristics for QRISK>10% population

Covariate relative to index (N (%) unless specified)	Total (N=1,267,660)	COPD diagnosis (N=72,682, 5.7%)	No COPD history (N=1,194,978, 4.3%)
Study follow-up in years (median, IQR)	5.5 [2.4, 9.7]	3.8 [1.7, 6.9]	5.7 [2.5, 9.9]
All range (1 day to 12 years)			
**Age at entry (mean, SD)**	71.1 (10.7)	71.7 (9.4)	71.1 (10.8)
**Sex**			
Male	656,139 (51.8)	37,995 (52.3)	618,144 (51.7)
Female	611,521 (48.2)	34,687 (47.7)	576,834 (48.3)
**Smoking status**			
Never-smoker	238,288 (18.8)	2,405 (3.3)	235,883 (19.7)
Ex-smoker	761,048 (60.0)	43,257 (59.5)	717,791 (60.1)
Current smoker	268,324 (21.2)	27,020 (37.2)	241,304 (20.2)
**Index of Multiple Deprivation quintile**			
1 Least Deprived	265,461 (20.9)	9,839 (13.5)	255,622 (21.4)
2	270,738 (21.4)	12,669 (17.4)	258,069 (21.6)
3	250,648 (19.8)	13,561 (18.7)	237,087 (19.8)
4	247,388 (19.5)	16,237 (22.3)	231,151 (19.3)
5 Most Deprived	231,281 (18.2)	20,315 (28.0)	210,966 (17.7)
Missing/Unknown	2,144 (0.2)	61 (0.1)	2,083 (0.2)
**Comorbidities***			
BMI (kg/m^2)			
Normal (18.5 to <25)	233,228 (18.4)	16,252 (22.4)	216,976 (18.2)
Underweight (<18.5)	15,442 (1.2)	2,091 (2.9)	13,351 (1.1)
Overweight (25 to <30)	362,337 (28.6)	20,472 (28.2)	341,865 (28.6)
Obese (30+)	441,893 (34.9)	30,718 (42.3)	411,175 (34.4)
Missing/Unknown	214,760 (16.9)	3,149 (4.3)	211,611 (17.7)
Chronic Kidney Disease			
Yes	548,858 (43.3)	34,378 (47.3)	514,480 (43.1)
No	543,979 (42.9)	31,228 (43.0)	512,751 (42.9)
Missing	174,823 (13.8)	7,076 (9.7)	167,747 (14.0)
Type II Diabetes	526,887 (41.6)	28,567 (39.3)	498,320 (41.7)
Hypertension	741,018 (58.5)	41,513 (57.1)	699,505 (58.5)
GORD	193,765 (15.3)	15,464 (21.3)	178,301 (14.9)
Asthma	347,517 (27.4)	43,517 (59.9)	304,000 (25.4)
Depression	189,135 (14.9)	16,755 (23.1)	172,380 (14.4)
Anxiety	152,769 (12.1)	12,604 (17.3)	140,165 (11.7)
**Cardiovascular-related prescriptions****			
Any	941,161 (74.2)	57,291 (78.8)	883,870 (74.0)
Diuretics	414,368 (32.7)	28,346 (39.0)	386,022 (32.3)
Beta blockers	229,340 (18.1)	7,506 (10.3)	221,834 (18.6)
Hypertension and HF drugs	606,748 (47.9)	34,444 (47.4)	572,304 (47.9)
Nitrates, CCBs, Antianginals	437,810 (34.5)	27,483 (37.8)	410,327 (34.3)
Antiplatelets	352,970 (27.8)	21,777 (30.0)	331,193 (27.7)
Statins	570,673 (45.0)	35,620 (49.0)	535,053 (44.8)

Comorbidities defined as ‘ever’ in history except for BMI (nearest to study start within five years), cardiovascular medications (defined in the last two years), and CKD (nearest within past 2 years, as eGFR mL/min as a measure of kidney function, e.g., 40 mL/min eGFR = 40% kidney function

** n(%) described for cardiovascular prescription categories may not be mutually exclusive, as they can be taken in combination elsewhere in the table (e.g., patient prescribed a diuretic and statins)

Abbreviations: MACE = major adverse cardiovascular event (acute coronary syndrome; arrhythmias; heart failure; ischaemic stroke); COPD = chronic obstructive pulmonary disease; BMI = body mass index, CKD = chronic kidney disease; eGFR = estimated glomerular filtration rate; GORD = gastro-oesophageal reflux disease; HF = heart failure; CCB = calcium channel blocker

Incident COPD versus no COPD (Exposure 1b)

Table E14: Relationship between incident COPD and subsequent MACE amongst five populations at elevated risk of MACE

Cohort	Metric	Outcome<br>*MACE*<br>*Incident COPD*	 <br>*No COPD*	 *ACS*<br>*Incident COPD*	 <br>*No COPD*	 *Arrhythmia*<br>*Incident COPD*	 <br>*No COPD*	 *Ischaemic stroke*<br>*Incident COPD*	 <br>*No COPD*	 *Heart failure*<br>*Incident COPD*	 <br>*No COPD*	 *CV-specific death*<br>*Incident COPD*	 <br>*No COPD*
**CKD**	*Crude N*	1,265	531,210	1,750	692,437	1,662	650,768	1,935	727,923	1,863	739,016	1,265	531,210
	Crude N(%) outcome	281 (22.2)	102,396 (19.3)	87 (5.0)	27,689 (4.0)	89 (5.4)	32,236 (5.0)	73 (3.8)	33,183 (4.6)	164 (8.8)	43,198 (5.9)	121 (9.6)	44,877 (8.5)
	Crude HR (95%CI)	**1.34 (1.19, 1.51)**		**1.41 (1.14, 1.74)**		1.25 (1.02, 1.54)		0.95 (0.75, 1.19)		**1.76 (1.51, 2.05)**		**1.30 (1.09, 1.56)**	
	aHR (95%CI)	**1.28 (1.13, 1.45)**		1.29 (1.04, 1.60)		1.10 (0.88, 1.37)		0.98 (0.77, 1.24)		**1.53 (1.30, 1.79)**		**1.32 (1.10, 1.59)**	
	Sens: -BMI	**1.30 (1.15, 1.46)**		1.29 (1.05, 1.59)		1.16 (0.94, 1.43)		0.95 (0.76, 1.20)		**1.57 (1.35, 1.83)**		**1.31 (1.09, 1.560**	
**T2DM**	*Crude N*	1,222	619,585	1,450	698,926	1,497	704,065	1,593	733,287	1,585	744,543	1,222	619,585
	Crude N(%) outcome	180 (14.7)	76,053 (12.3)	60 (4.1)	24,947 (3.6)	52 (3.5)	24,043 (3.4)	46 (2.9)	22,243 (3.0)	73 (4.6)	28,274 (3.8)	78 (6.4)	25,567 (4.1)
	Crude HR (95%CI)	**1.49 (1.29, 1.73)**		**1.41 (1.10, 1.82)**		1.28 (0.98, 1.68)		1.19 (0.89, 1.58)		**1.54 (1.22, 1.93)**		**1.95 (1.56, 2.43)**	
	aHR (95%CI)	1.19 (1.02, 1.40)		1.13 (0.86, 1.49)		1.09 (0.82, 1.45)		0.99 (0.73, 1.35)		1.16 (0.91, 1.48)		**1.69 (1.33, 2.15)**	
	Sens: -CKD	1.21 (104, 1.41)		1.18 (0.90, 1.53)		1.06 (0.80, 1.41)		0.97 (0.71, 1.31)		1.22 (0.96, 1.54)		**1.68 (1.33, 2.12)**	
	Sens: -BMI	**1.23 (1.06, 1.44)**		1.15' (0.88, 1.50)		1.14 (0.86, 1.50)		1.02 (0.76, 1.37)		1.14 (0.89, 1.45)		**1.74 (1.38, 2.19)**	
**MACE history**	*Crude N*	904	452,423	904	452,423	904	452,423	904	452,423	904	452,423	904	452,423
	Crude N(%) outcome	296 (32.7)	126,123 (27.9)	57 (6.3)	25,894 (5.7)	127 (14.1)	61,360 (13.6)	39 (4.3)	22,618 (5.0)	121 (13.4)	31,919 (7.1)	17 (1.9)	6,311 (1.4)
	Crude HR (95%CI)	**1.24 (1.11, 1.39)**		1.15 (0.88, 1.49)		1.07 (0.90, 1.27)		0.89 (0.65, 1.22)		**2.07 (1.73, 2.47)**		1.42 (0.88, 2.29)	
	aHR (95%CI)	1.14 (0.99, 1.30)		1.04 (0.77, 1.40)		1.11 (0.91, 1.36)		0.70 (0.47, 1.03)		**1.72 (1.41, 2.10)**		0.98 (0.53, 1.82)	
	Sens: -CKD	**1.19 (1.05, 1.34)**		1.04 (0.79, 1.38)		1.11 (0.91, 1.34)		0.82 (0.58, 1.15)		**1.84 (1.53, 2.22)**		0.97 (0.53, 1.74)	
	Sens: -BMI	1.12 (0.98, 1.27)		0.97 (0.72, 1.30)		1.13 (0.93, 1.37)		0.70 (0.48, 1.02)		**1.66 (1.37, 2.02)**		1.05 (0.60, 1.85)	

Incident COPD: first diagnosis of COPD occurred within six weeks (in either direction) of the first evidence of cohort-specific disease (i.e., CKD, T2DM and MACE).
Green model = main model presented in manuscript; adjusted for age, sex, socioeconomic deprivation, smoking status, hypertension, asthma, GORD, depression, anxiety, CVD medications, T2DM (except T2DM cohort), BMI (except obesity cohort), CKD (except CKD cohort) and cardiovascular history (MACE-subtypes only)
Sens: -VARIABLE = sensitivity analysis citing the covariate removed from the analysis
Abbreviations: COPD = chronic obstructive pulmonary disease; MACE = major adverse cardiovascular event; CKD = chronic kidney disease; T2Dm = Type-II diabetes mellitus; ACS = acute coronary syndrome; HR = hazard ratio; aHR = adjusted hazard ratio, BMI = body mass index

At risk of COPD versus no COPD and no risk (Exposure 1c; without infection)

Table E15: Relationship between being at risk of COPD in the absence of history of frequent antibiotic-treated lower respiratory tract infections and subsequent MACE amongst five populations at elevated risk of MACE

Cohort	Metric	Outcome MACE		ACS		Arrhythmia		Ischaemic stroke		Heart failure		CV-specific death	
		At risk of COPD	*No COPD*	*At risk of COPD*	*No COPD*	*At risk of COPD*	*No COPD*	*At risk of COPD*	*No COPD*	*At risk of COPD*	*No COPD*	*At risk of COPD*	*No COPD*
**CKD**	*Crude N*	314,001	217,997	314,001	379,482	314,001	337,787	314,001	415,086	314,001	426,143	314,001	217,997
	Crude N(%) outcome	62,998 (20.1)	39,590 (18.2)	13,077 (4.2)	14,665 (3.9)	14,749 (4.7)	17,543 (5.2)	13,323 (4.2)	19,905 (4.8)	13,807 (4.4)	29,493 (6.9)	28,258 (9.0)	16,702 (7.7)
	Crude HR (95%CI)	**1.10 (1.09, 1.12)**		0.96 (0.93, 0.98)		0.84 (0.82, 0.86)		0.79 (0.77, 0.80)		0.56 (0.55, 0.57)		**1.17 (1.15, 1.19)**	
	aHR (95%CI)	0.99 (0.95, 1.03)		0.95 (0.91, 1.00)		0.98 (0.93, 1.02)		0.96 (0.92, 1.00)		0.90 (0.86, 0.93)		1.00 (0.93, 1.07)	
	Sens: -BMI	0.97 (0.93, 1.01)		0.96 (0.92, 1.01)		0.98 (0.94, 1.03)		0.97 (0.93, 1.01)		0.91 (0.88, 0.94)		0.97 (0.91, 1.03)	
**T2DM**	*Crude N*	351,015	269,133	351,015	348,586	351,015	353,745	351,015	383,004	351,015	394,261	351,015	269,133
	Crude N(%) outcome	47,478 (13.5)	28,664 (10.7)	13,025 (3.7)	11,951 (3.4)	11,515 (3.3)	12,554 (3.6)	10,033 (2.9)	12,230 (3.2)	10,366 (3.0)	17,941 (4.6)	16,544 (4.7)	9,062 (3.4)
	Crude HR (95%CI)	**1.22 (1.20, 1.24)**		0.99 (0.96, 1.01)		0.86 (0.83, 0.88)		0.82 (0.79, 0.84)		0.58 (0.57, 0.60)		**1.33 (1.29, 1.39)**	
	aHR (95%CI)	1.00 (0.95, 1.05)		1.00 (0.94, 1.06)		0.99 (0.94, 1.04)		1.01 (0.96, 1.06)		0.94 (0.90, 0.98)		0.92 (0.84, 1.01)	
	Sens: -CKD	1.01 (0.96, 1.06)		0.98 (0.93, 1.04)		0.99 (0.94, 1.04)		1.00 (0.95, 1.06)		0.94 (0.90, 0.98)		0.92 (0.84, 1.01)	
	Sens: -BMI	1.00 (0.95, 1.05)		1.00 (0.94, 1.05)		0.98 (0.93, 1.03)		1.01 (0.96, 1.06)		0.93 (0.89, 0.97)		0.91 (0.83, 1.00)	
**Obesity**	*Crude N*	108,524	93,388	108,524	109,548	108,524	107,512	108,524	115,607	108,524	116,650	108,524	93,388
	Crude N(%) outcome	7,632 (7.0)	4,567 (4.9)	2,026 (1.9)	1,601 (1.5)	2,280 (2.1)	2,257 (2.1)	1,466 (1.4)	1,517 (1.3)	1,463 (1.4)	2,381 (2.0)	2,046 (1.9)	1,045 (1.1)
	Crude HR (95%CI)	**1.28 (1.24, 1.33)**		**1.12 (1.05, 1.19)**		0.88 (0.83, 0.93)		0.90 (0.83, 0.96)		0.57 (0.54, 0.61)		**1.48 (1.37, 1.60)**	
	aHR (95%CI)	1.07. (0.94, 1.22)		1.23 (1.04, 1.46)		0.98 (0.85, 1.13)		**1.30 (1.11, 1.53)**		0.91 (0.81, 1.03)		1.07 (0.82, 1.41)	
	Sens: -CKD	0.99 (0.88, 1.12)		1.20 (1.03, 1.40)		0.95 (0.83, 1.08)		1.20 (1.04, 1.39)		0.88 (0.79, 0.99)		0.99 (0.77, 1.28)	

Cohort	Metric	MACE		ACS		Arrhythmia		Ischaemic stroke		Heart failure		CV-specific death	
		At risk of COPD	*No COPD*	*At risk of COPD*	*No COPD*	*At risk of COPD*	*No COPD*	*At risk of COPD*	*No COPD*	*At risk of COPD*	*No COPD*	*At risk of COPD*	*No COPD*
**Age65+**	*Crude N*	324,612	244,693	324,612	310,277	324,612	300,801	324,612	328,023	324,612	340,923	324,612	244,693
	Crude N(%) outcome	43,192 (13.3)	28,377 (11.6)	8,991 (2.9)	9,644 (3.0)	11,840 (3.7)	11,981 (4.0)	9,513 (2.9)	10,955 (3.3)	7,521 (2.3)	12,849 (3.8)	15,915 (4.9)	10,006 (4.1)
	Crude HR (95%CI)	**1.12 (1.11, 1.14)**		0.97 (0.94, 1.00)		0.87 (0.85, 0.89)		0.83 (0.81, 0.85)		0.58 (0.56, 0.59)		**1.17 (1.14, 1.20)**	
	aHR (95%CI)	0.99 (0.93, 1.05)		1.00 (0.92, 1.07)		0.98 (0.92, 1.05)		1.03 (0.97, 1.10)		0.91 (0.86, 0.96)		0.97 (0.87, 1.08)	
	Sens: -CKD	0.99 (0.94, 1.05)		1.00 (0.93, 1.08)		0.98 (0.92, 1.04)		1.01 (0.95, 1.08)		0.91 (0.87, 0.96)		0.96 (0.87, 1.05)	
	Sens: -BMI	0.98 (0.93, 1.04)		0.99 (0.93, 1.07)		0.99 (0.93, 1.05)		1.05 (0.99, 1.12)		0.91 (0.87, 0.96)		0.97 (0.89, 1.07)	
**MACE history**	*Crude N*	269,844	183,483	269,844	183,483	269,844	183,483	269,844	183,483	269,844	183,483	269,844	183,483
	Crude N(%) outcome	75,301 (27.9)	51,118 (27.9)	16,058 (6.0)	9,893 (5.4)	35,467 (13.1)	26,020 (14.2)	13,828 (5.1)	8,829 (4.8)	19,343 (7.2)	12,697 (6.9)	3,917 (1.5)	2,411 (1.3)
	Crude HR (95%CI)	1.00 (0.99, 1.01)		**1.11 (1.08, 1.13)**		0.92 (0.90, 0.93)		**1.06 (1.04, 1.09)**		1.03 (1.01, 1.06)		**1.10 (1.05, 1.16)**	
	aHR (95%CI)	1.01 (0.97, 1.05)		1.10 (1.00, 1.21)		0.97 (0.92, 1.03)		0.95 (0.86, 1.05)		1.03 (0.95, 1.12)		1.16 (0.97, 1.40)	
	Sens: -CKD	1.00 (0.97, 1.04)		1.09 (1.00, 1.19)		0.97 (0.92, 1.03)		0.94 (0.86, 1.04)		1.01 (0.94, 1.09)		1.11 (0.93, 1.32)	
	Sens: -BMI	1.00 (0.96, 1.04)		1.10 (1.01, 1.21)		0.98 (0.93, 1.03)		0.94 (0.85, 1.03)		1.00 (0.93, 1.08)		1.15 (0.97, 1.35)	

At risk of COPD: history of smoking, age 40 years or older, and without asthma, without a COPD diagnostic code
Bold indicates positive statistical significance; red indicates negative statistical significance

Green model = main model presented in manuscript; adjusted for age, sex, socioeconomic deprivation, smoking status, hypertension, asthma, GORD, depression, anxiety, CVD medications, T2DM (except T2DM cohort), BMI (except obesity cohort), CKD (except CKD cohort) and cardiovascular history (MACE-subtypes only)
Sens: -VARIABLE = sensitivity analysis citing the covariate removed from the analysis

Abbreviations: COPD = chronic obstructive pulmonary disease; MACE = major adverse cardiovascular event; CKD = chronic kidney disease; T2Dm = Type-II diabetes mellitus; ACS = acute coronary syndrome; HR = hazard ratio; aHR = adjusted hazard ratio, BMI = body mass index

At risk of COPD versus no COPD and no risk (Exposure 1c; with infection)

Table E16: Relationship between being at risk of COPD (history of smoking, age 40 years or older, and without asthma) in the presence of history of frequent antibiotic-treated lower respiratory tract infections and subsequent MACE amongst five populations at elevated risk of MACE

Cohort	Metric	Outcome											
		MACE		***ACS***		***Arrhythmia***		***Ischaemic stroke***		***Heart failure***		***CV-specific death***	
		At risk of COPD with infections	*No COPD*	*At risk of COPD with infections*	*No COPD*	*At risk of COPD with infections*	*No COPD*	*At risk of COPD with infections*	*No COPD*	*At risk of COPD with infections*	*No COPD*	*At risk of COPD with infections*	*No COPD*
**CKD**	*Crude N*	2,336	529,662	2,336	691,147	2,336	649,452	2,336	726,751	2,336	737,808	2,336	529,662
	Crude N(%) outcome	479 (20.5)	102,109 (19.3)	106 (4.5)	27,636 (4.0)	99 (4.2)	32,193 (5.0)	86 (3.7)	33,142 (4.6)	128 (5.5)	43,172 (5.9)	212 (9.1)	44,748 (8.5)
	Crude HR (95%CI)	**1.32 (1.21, 1.45)**		**1.29 (1.07, 1.56)**		1.02 (0.84, 1.24)		0.93 (0.75, 1.14)		1.09 (0.92, 1.30)		**1.33 (1.16, 1.52)**	
	aHR (95%CI)	**1.18 (1.07, 1.30)**		1.25 (1.02, 1.54)		1.06 (0.85, 1.31)		1.10 (0.88, 1.38)		**1.34 (1.11, 1.63)**		1.10 (0.94, 1.28)	
	Sens: -BMI	**1.20 (1.10, 1.31)**		1.24 (1.02, 1.50)		1.09 (0.89, 1.32)		1.01 (0.82, 1.25)		**1.43 (1.20, 1.71)**		1.15 (1.00, 1.32)	
**T2DM**	*Crude N*	1,716	618,432	1,716	697,885	1,716	703,044	1,716	732,303	1,716	743,560	1,716	618,432
	Crude N(%) outcome	235 (13.7)	75,907 (12.3)	65 (3.8)	24,911 (3.6)	68 (4.0)	24,001 (3.4)	36 (2.1)	22,227 (3.0)	57 (3.3)	28,250 (3.8)	75 (4.4)	25,531 (4.1)
	Crude HR (95%CI)	**1.42 (1.25, 1.61)**		1.27 (1.00, 1.63)		**1.45 (1.14, 1.84)**		0.84 (0.60, 1.16)		1.09 (0.84, 1.41)		**1.35 (1.08, 1.70)**	
	aHR (95%CI)	1.16 (1.01, 1.33)		1.08 (0.82, 1.43)		**1.47 (1.14, 1.88)**		0.82 (0.57, 1.17)		1.31 (1.00, 1.72)		1.02 (0.79, 1.31)	
	Sens: -CKD	1.15 (1.00, 1.32)		1.08 (0.83, 1.42)		**1.44 (1.13, 1.85)**		0.84 (0.59, 1.19)		1.34 (1.03, 1.75)		0.99 (0.77, 1.27)	
	Sens: -BMI	**1.22 (1.07, 1.39)**		1.20 (0.94, 1.55)		**1.51 (1.18, 1.92)**		0.83 (0.59, 1.17)		1.33 (1.02, 1.73)		1.16 (0.92, 1.46)	
**Obesity**	*Crude N*	625	201,287	625	217,447	625	215,411	625	223,506	625	224,549	625	201,287
	Crude N(%) outcome	48 (7.7)	12,151 (6.0)	11 (1.8)	3,616 (1.7)	16 (2.6)	4,521 (2.1)	11 (1.8)	2,972 (1.3)	###	3,837 (1.7)	14 (2.2)	3,077 (1.5)
	Crude HR (95%CI)	**1.70 (1.28, 2.26)**		1.35 (0.75, 2.45)		1.61 (0.99, 2.63)		1.72 (0.95, 3.11)		0.86 (0.41, 1.80)		**1.96 (1.16, 3.31)**	
	aHR (95%CI)	1.39 (1.05, 1.86)		1.26 (0.69, 2.28)		1.66 (1.01, 2.72)		1.60 (0.86, 2.98)		0.94 (0.45, 1.97)		1.53 (0.90, 2.58)	

Cohort	Metric	***MACE***		***ACS***		***Arrhythmia***		***Ischaemic stroke***		***Heart failure***		***CV-specific death***	
		At risk of COPD with infections	*No COPD*	*At risk of COPD with infections*	*No COPD*	*At risk of COPD with infections*	*No COPD*	*At risk of COPD with infections*	*No COPD*	*At risk of COPD with infections*	*No COPD*	*At risk of COPD with infections*	*No COPD*
**Age65+**	*Crude N*	1,852	567,453	1,852	633,037	1,852	623,561	1,852	650,783	1,852	663,683	1,852	567,453
	Crude N(%) outcome	323 (17.4)	71,246 (12.6)	80 (4.3)	18,555 (2.9)	64 (3.5)	23,757 (3.8)	1,777 (4.1)	20,393 (3.1)	65 (3.5)	20,305 (3.1)	136 (7.3)	25,785 (4.5)
	Crude HR (95%CI)	**1.57 (1.40, 1.75)**		**1.60 (1.29, 2.00)**		0.99 (0.78, 1.27)		**1.41 (1.13, 1.77)**		1.26 (0.99, 1.61)		**1.79 (1.51, 2.12)**	
	aHR (95%CI)	**1.28 (1.13, 1.46)**		1.34 (1.03, 1.75)		1.04 (0.79, 1.36)		1.39 (1.06, 1.81)		1.21 (0.90, 1.63)		1.31 (1.07, 1.61)	
	Sens: -CKD	**1.25 (1.11, 1.41)**		1.32 (1.03, 1.69)		0.98 (0.92, 1.04)		1.01 (0.95, 1.08)		1.21 (0.91, 1.59)		**1.31 (1.08, 1.57)**	
	Sens: -BMI	**1.27 (1.13, 1.44)**		1.39 (1.09, 1.77)		0.97 (0.74, 1.26)		1.35 (1.05, 1.74)		1.40 (1.08, 1.82)		**1.32 (1.10, 1.59)**	
**MACE history**	*Crude N*	3,142	450,185	3,142	450,185	3,142	450,185	3,142	450,185	3,142	450,185	3,142	450,185
	Crude N(%) outcome	956 (30.4)	125,463 (27.9)	223 (7.1)	25,728 (5.7)	420 (13.4)	61,067 (13.6)	122 (3.9)	22,535 (5.0)	322 (10.3)	31,718 (7.1)	57 (1.8)	6,271 (1.4)
	Crude HR (95%CI)	**1.23 (1.15, 1.31)**		**1.38 (1.21, 1.57)**		1.08 (0.99, 1.19)		0.86 (0.72, 1.03)		**1.68 (1.51, 1.88)**		**1.49 (1.15, 1.93)**	
	aHR (95%CI)	**1.16 (1.08, 1.25)**		**1.28 (1.11, 1.48)**		1.13 (1.01, 1.25)		0.79 (0.65, 0.97)		**1.34 (1.19, 1.51)**		1.13 (0.84, 1.53)	
	Sens: -CKD	**1.18 (1.10, 1.26)**		**1.33 (1.16, 1.53)**		1.15 (1.03, 1.27)		0.77 (0.63, 0.93)		**1.35 (1.20, 1.51)**		1.11 (0.83, 1.49)	
	Sens: -BMI	**1.14 (1.07, 1.22)**		**1.22 (1.06, 1.40)**		**1.15 (1.04, 1.27)**		0.75 (0.63, 0.91)		**1.30 (1.16, 1.45)**		1.11 (0.84, 1.47)	

At risk of COPD: history of smoking, age 40 years or older, without asthma, and with two or more lower respiratory tract infections requiring antibiotic treatment within the two years before start of follow-up, without a COPD diagnostic code
Bold indicates positive statistical significance; red indicates negative statistical significance

Green model = main model presented in manuscript; adjusted for age, sex, socioeconomic deprivation, smoking status, hypertension, asthma, GORD, depression, anxiety, CVD medications, T2DM (except T2DM cohort), BMI (except obesity cohort), CKD (except CKD cohort) and cardiovascular history (MACE-subtypes only)
Sens: -VARIABLE = sensitivity analysis citing the covariate removed from the analysis
Too few to report (as per CPRD patient confidentiality policy)

Abbreviations: COPD = chronic obstructive pulmonary disease; MACE = major adverse cardiovascular event; CKD = chronic kidney disease; T2Dm = Type-II diabetes mellitus; ACS = acute coronary syndrome; HR = hazard ratio; aHR = adjusted hazard ratio, BMI = body mass index

KAPLAN MEIER (KM) PLOTS: ICS EXPOSURE

Chronic Kidney Disease (CKD)

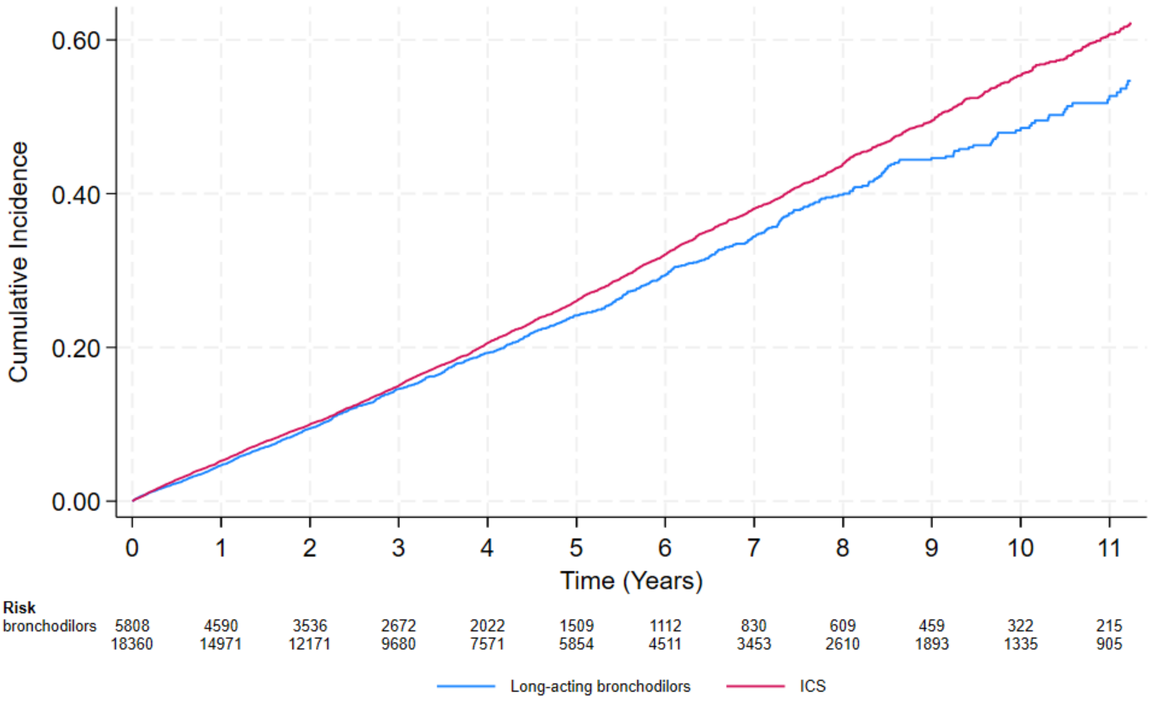

Figure E17: KM plot for pre-existing ICS versus no ICS analysis for CKD cohort

Type-II Diabetes Mellitus (T2DM)

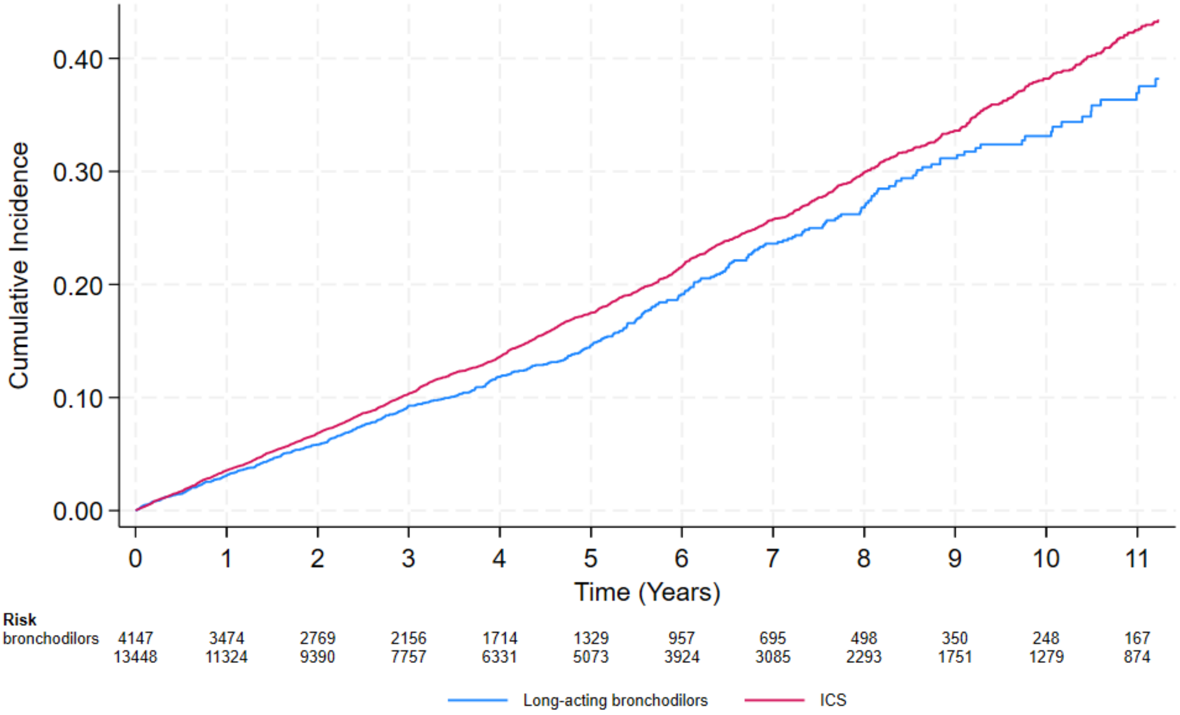

Figure E18: KM plot for pre-existing ICS versus no ICS analysis for T2DM cohort

Obesity

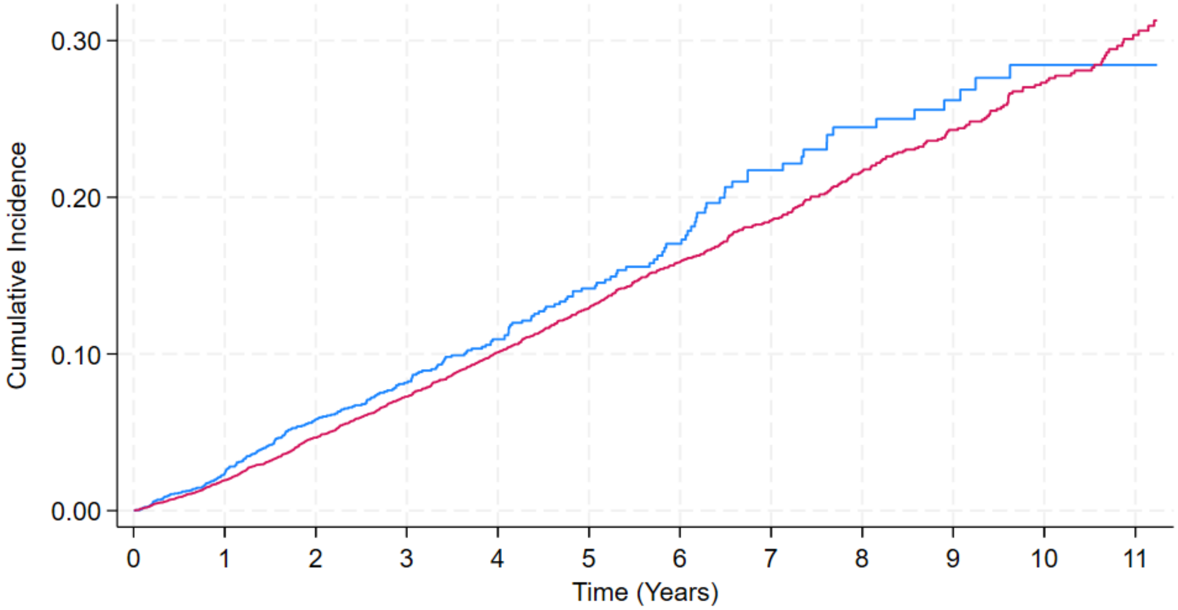

Number at Risk

	0	1	2	3	4	5	6	7	8	9	10	11
Long-acting bronchodilors	2637	2351	1715	1203	808	563	373	250	192	155	103	68
ICS	8345	7554	6140	4913	3801	2806	2110	1588	1244	975	687	383

Long-acting bronchodilors — ICS

Figure E19: KM plot for pre-existing ICS versus no ICS analysis for Obesity cohort

MACE history

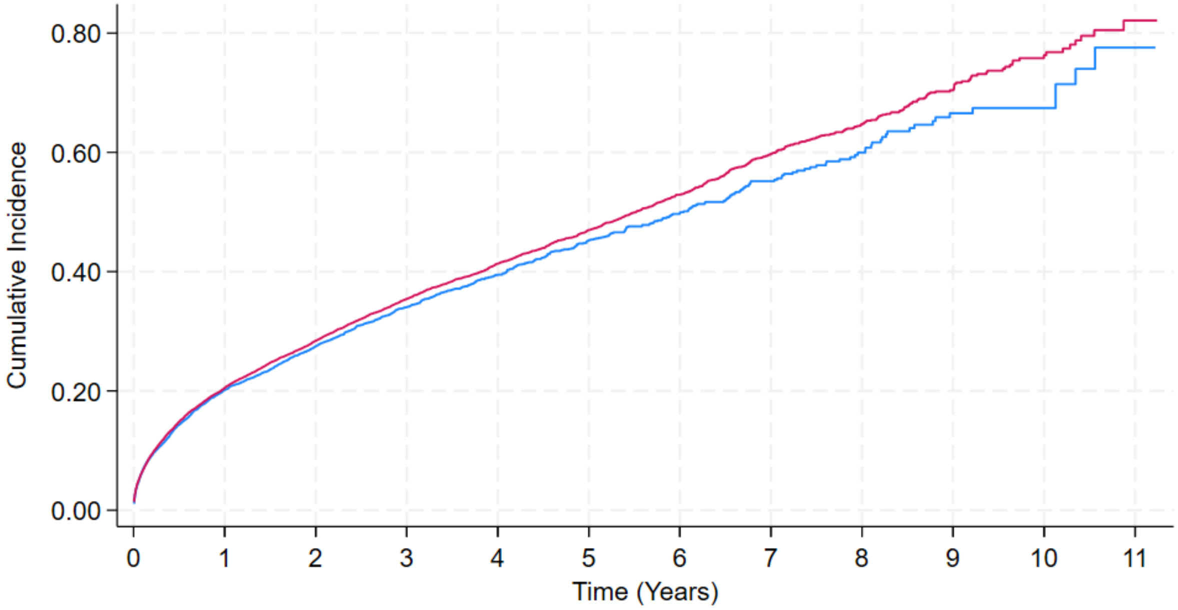

Number at Risk

	0	1	2	3	4	5	6	7	8	9	10	11
Long-acting bronchodilors	8453	5072	3449	2288	1553	1049	691	429	247	142	56	8
ICS	25661	15611	10880	7651	5355	3664	2408	1501	893	477	200	42

Long-acting bronchodilors — ICS

Figure E20: KM plot for pre-existing ICS versus no ICS analysis for MACE history cohort

Age65+

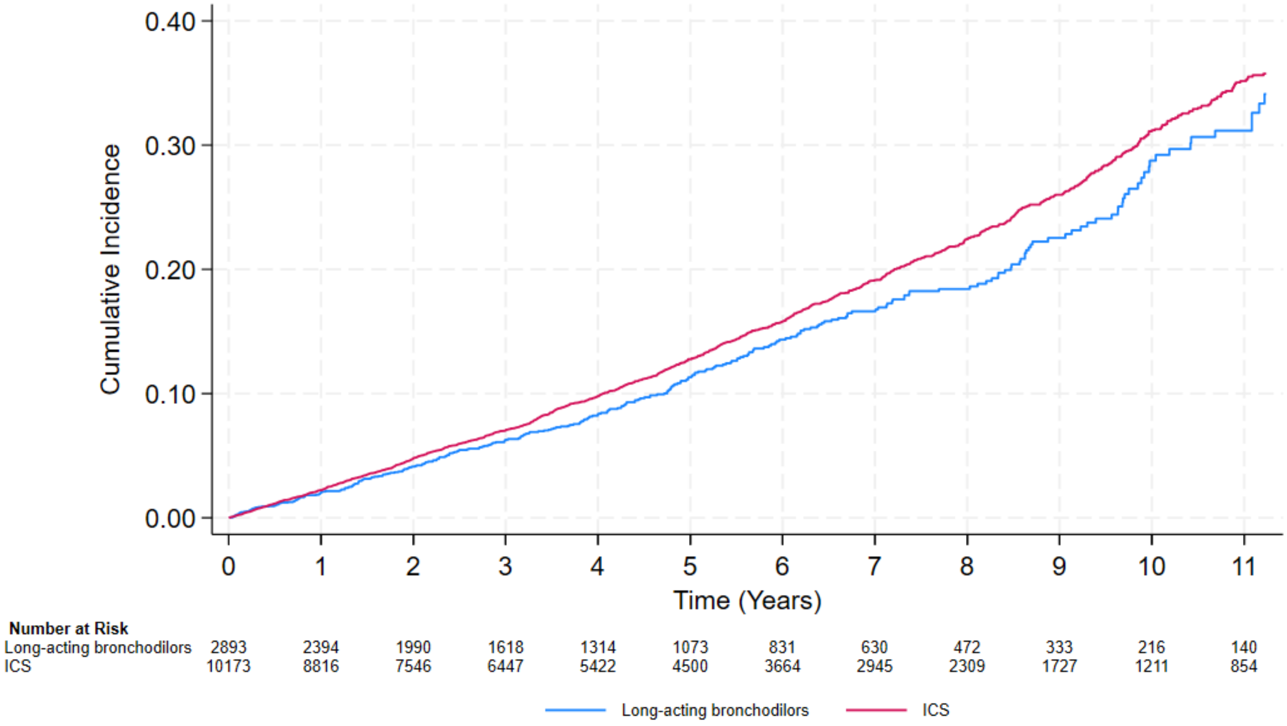

Figure E21: KM plot for pre-existing ICS versus no ICS analysis for Age65+ cohort

SENSITIVITY ANALYSIS RESULTS: ICS EXPOSURE

Table E17: Relationship between ICS and subsequent MACE amongst five populations at elevated risk of MACE

Cohort	Metric	Outcome: *MACE*		*ACS*		*Arrhythmia*		*Ischaemic stroke*		*Heart failure*		*CV-specific death*	
		ICS	*No ICS*	*ICS*	*No ICS*	*ICS*	*No ICS*	*ICS*	*No ICS*	*ICS*	*No ICS*	*ICS*	*No ICS*
**CKD**	Crude N	18,360	5,808	18,360	5,808	18,360	5,808	18,360	5,808	18,360	5,808	18,360	5,808
	Crude N(%) outcome	3,918 (21.3)	998 (17.2)	842 (4.6)	202 (3.5)	893 (4.9)	250 (4.3)	591 (3.2)	173 (3.0)	1,011 (5.5)	234 (4.0)	1,704 (9.3)	439 (7.6)
	Crude HR (95%CI)	1.09 (1.02, 1.17)		1.16 (1.00, 1.36)		0.98 (0.85, 1.12)		0.95 (0.80, 1.12)		1.19 (1.03, 1.37)		1.07 (0.96, 1.18)	
	aHR (95%CI)	1.05 (0.97, 1.14)		1.11 (0.92, 1.32)		0.92 (0.78, 1.09)		0.93 (0.76, 1.14)		1.11 (0.94, 1.31)		1.06 (0.94, 1.21)	
	Sens: psHR(95%CI)	1.05 (0.97, 1.15)		1.11 (0.93, 1.33)		0.93 (0.79, 1.10)		0.93 (0.76, 1.14)		1.10 (0.93, 1.31)		1.06 (0.93, 1.20)	
	Sens: -BMI	1.04 (0.96, 1.13)		1.10 (0.92, 1.31)		0.94 (0.80, 1.10)		0.93 (0.76, 1.14)		1.09 (0.93, 1.29)		1.04 (0.92, 1.18)	
	Sens: -MRC	1.08 (0.99, 1.17)		1.11 (0.93, 1.32)		0.95 (0.81, 1.12)		0.95 (0.78, 1.15)		1.13 (0.96, 1.34)		1.10 (0.97, 1.24)	
	Sens: -GOLD	1.05 (0.98, 1.14)		1.10 (0.93, 1.30)		0.92 (0.79, 1.07)		0.96 (0.80, 1.17)		1.11 (0.95, 1.30)		1.06 (0.94, 1.20)	
**T2DM**	Crude N	13,448	4,147	13,448	4,147	13,448	4,147	13,448	4,147	13,448	4,147	13,448	4,147
	Crude N(%) outcome	2,165 (16.1)	519 (12.5)	551 (4.1)	131 (3.2)	509 (3.8)	129 (3.1)	354 (2.6)	82 (2.0)	527 (3.9)	143 (3.5)	785 (5.8)	171 (4.1)
	Crude HR (95%CI)	1.13 (1.03, 1.25)		1.14 (0.95, 1.38)		1.06 (0.88, 1.29)		1.16 (0.91, 1.48)		0.97 (0.81, 1.17)		1.21 (1.03, 1.43)	
	aHR (95%CI)	1.06 (0.94, 1.18)		1.09 (0.87, 1.36)		1.05 (0.83, 1.31)		1.21 (0.90, 1.61)		0.87 (0.70, 1.09)		1.11 (0.91, 1.35)	
	Sens: psHR(95%CI)	1.05 (0.94, 1.18)		1.09 (0.87, 1.36)		1.05 (0.83, 1.31)		1.22 (0.92, 1.64)		0.85 (0.68, 1.07)		1.12 (0.92, 1.36)	
	Sens: -BMI	1.05 (0.94, 1.18)		1.10 (0.88, 1.37)		1.03 (0.83, 1.29)		1.23 (0.92, 1.63)		0.87 (0.69, 1.08)		1.10 (0.91, 1.33)	
	Sens: -CKD	1.05 (0.94, 1.18)		1.09 (0.88, 1.36)		1.05 (0.84, 1.31)		1.19 (0.90, 1.58)		0.87 (0.70, 1.08)		1.11 (0.91, 1.34)	
	Sens: -MRC	1.09 (0.98, 1.22)		1.11 (0.89, 1.38)		1.07 (0.86, 1.34)		1.29 (0.97, 1.71)		0.86 (0.70, 1.07)		1.16 (0.96, 1.40)	
	Sens: -GOLD	1.09 (0.98, 1.21)		1.10 (0.89, 1.36)		1.03 (0.83, 1.28)		1.23 (0.93, 1.63)		0.91 (0.74, 1.13)		1.21 (1.00, 1.47)	

Cohort	Metric	*MACE*		*ACS*		*Arrhythmia*		*Ischaemic stroke*		*Heart failure*		*CV-specific death*	
		ICS	*No ICS*	*ICS*	*No ICS*	*ICS*	*No ICS*	*ICS*	*No ICS*	*ICS*	*No ICS*	*ICS*	*No ICS*
**Obesity**	Crude N	8,345	2,637	8,345	2,637	8,345	2,637	8,345	2,637	8,345	2,637	8,345	2,637
	Crude N(%) outcome	936 (11.2)	253 (9.6)	236 (2.8)	70 (2.7)	245 (2.9)	59 (2.2)	137 (1.6)	25 (1.0)	264 (3.2)	78 (3.0)	279 (3.3)	80 (3.0)
	Crude HR (95%CI)	0.90 (0.78, 1.04)		0.83 (0.64, 1.09)		1.01 (0.76, 1.35)		1.28 (0.84, 1.97)		0.80 (0.62, 1.03)		0.82 (0.64, 1.06)	
	aHR (95%CI)	0.97 (0.81, 1.17)		1.03 (0.72, 1.47)		1.19 (0.82, 1.73)		1.24 (0.73, 2.11)		0.70 (0.51, 0.97)		1.00 (0.71, 1.41)	
	Sens: psHR(95%CI)	0.97 (0.80, 1.16)		1.01 (0.71, 1.45)		1.21 (0.83, 1.76)		1.26 (0.74, 2.15)		0.69 (0.50, 0.95)		0.97 (0.69, 1.37)	
	Sens: -CKD	0.96 (0.82, 1.13)		0.94 (0.69, 1.28)		1.17 (0.84, 1.62)		1.27 (0.79, 2.04)		0.71 (0.53, 0.96)		0.90 (0.67, 1.21)	
	Sens: -MRC	0.97 (0.81, 1.15)		0.95 (0.68, 1.32)		1.15 (0.81, 1.64)		1.31 (0.79, 2.18)		0.71 (0.52, 0.98)		0.97 (0.70, 1.35)	
	Sens: -GOLD	0.99 (0.84, 1.18)		1.02 (0.73, 1.44)		1.11 (0.79, 1.58)		1.48 (0.88, 2.48)		0.74 (0.54, 1.01)		1.02 (0.74, 1.40)	
**Age65+**	Crude N	10,173	2,893	10,173	2,893	10,173	2,893	10,173	2,893	10,173	2,893	10,173	2,893
	Crude N(%) outcome	1,431 (14.1)	311 (10.8)	350 (3.4)	68 (2.4)	392 (3.9)	85 (2.9)	247 (2.4)	64 (2.2)	278 (2.7)	48 (1.7)	461 (4.5)	103 (3.6)
	Crude HR (95%CI)	1.13 (1.00, 1.27)		1.27 (0.98, 1.65)		1.10 (0.87, 1.39)		0.92 (0.70, 1.22)		1.39 (1.02, 1.88)		1.08 (0.87, 1.34)	
	aHR (95%CI)	1.09 (0.92, 1.29)		1.36 (0.96, 1.92)		1.09 (0.80, 1.50)		0.90 (0.62, 1.31)		1.46 (0.95, 2.24)		0.88 (0.66, 1.18)	
	Sens: psHR(95%CI)	1.07 (0.91, 1.27)		1.38 (0.97, 1.96)		1.04 (0.75, 1.42)		0.92 (0.63, 1.34)		1.38 (0.90, 2.12)		0.86 (0.64, 1.15)	
	Sens: -BMI	1.08 (0.91, 1.27)		1.37 (0.97, 1.94)		1.07 (0.78, 1.46)		0.88 (0.61, 1.28)		1.28 (0.86, 1.92)		0.88 (0.66, 1.17)	
	Sens: -CKD	1.05 (0.91, 1.21)		1.32 (0.97, 1.81)		1.05 (0.80, 1.39)		0.86 (0.63, 1.19)		1.49 (1.00, 2.21)		0.84 (0.65, 1.08)	
	Sens: -MRC	1.14 (0.97, 1.34)		1.44 (1.02, 2.03)		1.14 (0.83, 1.55)		0.97 (0.67, 1.40)		1.45 (0.96, 2.17)		0.93 (0.70, 1.24)	
	Sens: -GOLD	1.11 (0.95, 1.30)		1.30 (0.94, 1.81)		1.07 (0.79, 1.44)		0.94 (0.65, 1.35)		1.37 (0.92, 2.02)		0.98 (0.75, 1.29)	
**MACE history**	Crude N	25,661	8,453	25,661	8,543	25,661	8,453	25,661	8,453	25,661	8,453	25,661	8,453
	Crude N(%) outcome	7,054 (27.5)	2,166 (25.6)	1,564 (6.1)	465 (5.5)	2,889 (11.3)	898 (10.6)	1,015 (4.0)	334 (4.0)	2,568 (10.0)	762 (9.0)	427 (1.7)	112 (1.3)
	Crude HR (95%CI)	1.04 (1.00, 1.10)		1.07 (0.97, 1.19)		1.03 (0.95, 1.11)		0.96 (0.85, 1.09)		1.07 (0.98, 1.15)		1.19 (0.97, 1.47)	
	aHR (95%CI)	1.03 (0.97, 1.09)		1.01 (0.89, 1.14)		1.03 (0.94, 1.13)		1.00 (0.86, 1.17)		1.01 (0.92, 1.12)		1.00 (0.77, 1.31)	

Cohort	Metric	*MACE*	*ACS*	*Arrhythmia*	*Ischaemic stroke*	*Heart failure*	*CV-specific death*
	Sens: psHR(95%CI)	1.02 (0.96, 1.09)	1.00 (0.88, 1.14)	1.03 (0.94, 1.13)	1.00 (0.85, 1.16)	1.00 (0.91, 1.11)	1.01 (0.77, 1.31)
	Sens: -BMI	1.03 (0.97, 1.09)	1.01 (0.89, 1.15)	1.02 (0.93, 1.12)	1.00 (0.86, 1.17)	1.01 (0.92, 1.12)	1.00 (0.77, 1.30)
	Sens: -CKD	1.02 (0.96, 1.08)	1.02 (0.91, 1.16)	1.02 (0.94, 1.12)	1.02 (0.88, 1.18)	0.99 (0.90, 1.09)	1.04 (0.81, 1.33)
	Sens: -MRC	1.03 (0.97, 1.09)	1.01 (0.89, 1.15)	1.02 (0.93, 1.11)	1.03 (0.88, 1.20)	1.02 (0.93, 1.12)	1.07 (0.83, 1.39)
	Sens: -GOLD	1.03 (0.98, 1.09)	0.99 (0.88, 1.12)	1.05 (0.96, 1.15)	0.98 (0.85, 1.14)	1.04 (0.94, 1.14)	1.02 (0.80, 1.31)

ICS: at least one prescription for ICS in the year preceding follow-up (ICS = ICS monotherapy, ICS-LABA, ICS-LAMA, or ICS-LABA-LAMA)
No ICS: at least one prescription for a long-acting bronchodilator in the year preceding follow-up (LABA, LAMA, or LABA-LAMA)

Green model = main model presented in manuscript; adjusted for age, sex, socioeconomic deprivation, smoking status, hypertension, asthma, GORD, depression, anxiety, CVD medications, T2DM (except T2DM cohort), BMI (except obesity cohort), CKD (except CKD cohort), cardiovascular history (MACE-subtypes only), COPD exacerbations, MRC dyspnoea group, GOLD group, and short-acting bronchodilators

Sens: psHR = doublu robust propensity score adjusted models (adjusted for all main model variables as well as propensity scores generated using covariates)
Sens: -VARIABLE = sensitivity analysis citing the covariate removed from the analysis

Abbreviations: COPD = chronic obstructive pulmonary disease; MACE = major adverse cardiovascular event; CKD = chronic kidney disease; T2Dm = Type-II diabetes mellitus; ACS = acute coronary syndrome; HR = hazard ratio; aHR = adjusted hazard ratio, BMI = body mass index

TIME INTERACTION MODELS (ANNUAL HAZARD RATIOS)

Chronic Kidney Disease (CKD)

Table E18: Yearly rate of HF among people with CKD during follow-up, amongst people who are at risk of COPD without infections compared with people with no COPD and without being at risk of COPD, where the Proportional Hazards Assumption was violated

Exposure	Average aHR (95% CI)	Year 0	Year 1	Year 2	Year 3	Year 4	Year 5	Year 6	Year 7	Year 8	Year 9	Year 10	Year 11 (Jan to March)
At risk of COPD without infection (vs. no COPD or COPD risk)	0.90 (0.86-0.93)	0.80 (0.76-0.84) <0.0001	0.82 (0.79-0.86) <0.0001	0.84 (0.81-0.87) <0.0001	0.86 (0.83-0.89) <0.0001	0.88 (0.85-0.91) <0.0001	0.90 (0.87-0.93) <0.0001	0.92 (0.89-0.96) <0.0001	0.94 (0.89, 0.96 0.0035	0.97 (0.93-1.01 0.1136	0.99 (0.94-1.04) 0.6554	1.01 (0.96-1.07) 0.6258	1.04 (0.98-1.10) 0.2130
Statistically significant: blue cells													

Table E19: Yearly rate of HF among people with CKD during follow-up, amongst people who are at risk of COPD with infections compared with people with no COPD and without being at risk of COPD, where the Proportional Hazards Assumption was violated

Exposure	Average aHR (95% CI)	Year 0	Year 1	Year 2	Year 3	Year 4	Year 5	Year 6	Year 7	Year 8	Year 9	Year 10	Year 11 (Jan to March)
At risk of COPD without infection (vs. no COPD or COPD risk)	1.34 (1.11, 1.63)	1.76 (1.29-2.40) P=0.0004	1.64 (1.26-2.13) P=0.0002	1.53 (1.23-1.92) P=0.0003	1.43 (1.23-1.92) P=0.0003	1.34 (1.11-1.63) P=0.0027	1.25 (1.02-1.55) P=0.0343	1.17 (0.92-1.50) P=0.20244	1.10 (0.82-1.47) P=0.5355	1.03 (0.73-1.5) P=0.8877	0.96 (0.64-1.43) P=0.8357	0.90 (0.57-1.42) P=0.6407	0.84 (0.50-1.41) P=0.5064
Statistically significant: blue cells													

Type-II Diabetes Mellitus (T2DM)

Table E20: Yearly rate of MACE among people with T2DM during follow-up, amongst people with COPD pre-diagnosis compared with no COPD, where the Proportional Hazards Assumption was violated

Exposure	Average aHR (95% CI)	Year 0	Year 1	Year 2	Year 3	Year 4	Year 5	Year 6	Year 7	Year 8	Year 9	Year 10	Year 11 (Jan to March)
Pre-existing COPD (vs. no COPD)	1.30 (1.26-1.35)	1.40 (1.33-1.47) p<0.0001	1.37 (1.31-1.44) p<0.0001	1.35 (1.30-1.40) p<0.0001	1.32 (1.28-1.37) p<0.0001	1.30 (1.26-1.34) p<0.0001	1.28 (1.23-1.32) p<0.0001	1.25 (1.21-1.31) p<0.0001	1.23 (1.18-1.29) p<0.0001	1.21 (1.14-1.28) p<0.0001	1.19 (1.11-1.27) p<0.0001	1.17 (1.08-1.26) p<0.0001	1.15 (1.05-1.25) p=0.002
Statistically significant: gold cells													

Table E21: Yearly rate of heart failure among people with T2DM during follow-up, amongst people with COPD pre-diagnosis compared with no COPD, where the Proportional Hazards Assumption was violated

Exposure	Average aHR (95% CI)	Year 0	Year 1	Year 2	Year 3	Year 4	Year 5	Year 6	Year 7	Year 8	Year 9	Year 10	Year 11 (Jan to March)
Pre-existing COPD (vs. no COPD)	1.44 (1.38-1.51)	1.61 (1.49-1.74) p<0.0001	1.57 (1.47-1.68) p<0.0001	1.53 (1.44-1.61) p<0.0001	1.49 (1.41-1.56) p<0.0001	1.45 (1.38-1.52) p<0.0001	1.41 (1.34-1.48) p<0.0001	1.37 (1.30-1.45) p<0.0001	1.33 (1.25-1.43) p<0.0001	1.30 (1.20-1.41) p<0.0001	1.27 (1.15-1.39) p<0.0001	1.23 (1.11-1.37) p<0.0001	1.20 (1.06-1.35) p=0.003
Statistically significant: gold cells													

Obesity [none]

MACE history

Table E22: Yearly rate of MACE among people with MACE history during follow-up, amongst people with COPD pre-diagnosis compared with no COPD, where the Proportional Hazards Assumption was violated

**Exposure**	Average aHR (95% CI)	**Year 0**	**Year 1**	**Year 2**	**Year 3**	**Year 4**	**Year 5**	**Year 6**	**Year 7**	**Year 8**	**Year 9**	**Year 10**	**Year 11 (Jan to March)**
Pre-existing COPD (vs. no COPD)	1.04 (1.02, 1.06)	0.97 (0.94-0.99) 0.0031	1.02 (1.00-1.04) 0.0637	1.08 (1.05-1.10) <0.0001	1.13 (1.11-1.16) <0.0001	1.20 (1.16-1.24) <0.0001	1.26 (1.21-1.32) <0.0001	1.33 (1.27-1.40) <0.0001	1.41 (1.33-1.49) <0.0001	1.49 (1.39-1.59) <0.0001	1.57 (1.45-1.69) <0.0001	1.65 (1.52-1.80) <0.0001	1.75 (1.59-1.92 <0.0001
Statistically significant: purple cells													

Table E23: Yearly rate of ACS among people with MACE history during follow-up, amongst people with COPD pre-diagnosis compared with no COPD, where the Proportional Hazards Assumption was violated

Exposure	Average aHR (95% CI)	Year 0	Year 1	Year 2	Year 3	Year 4	Year 5	Year 6	Year 7	Year 8	Year 9	Year 10	Year 11 (Jan to March)
Pre-existing COPD (vs. no COPD)	1.06 (1.02, 1.10)	0.97 (0.92-1.02) 0.2745	1.03 (0.98-1.07) 0.2510	1.08 (1.04-1.13) 0.0003	1.14 (1.08-1.20) <0.0001	1.20 (1.13-1.28) <0.0001	1.27 (1.17-1.37) <0.0001	1.33 (1.21-1.47) <0.0001	1.41 (1.26-1.58) <0.0001	1.48 (1.30-1.69) <0.0001	1.56 (1.35-1.82) <0.0001	1.65 (1.39-1.95) <0.0001	1.74 (1.44-2.09) <0.0001
Statistically significant: purple cells													

Table E24: Yearly rate of HF among people with MACE history during follow-up, amongst people with COPD pre-diagnosis compared with no COPD, where the Proportional Hazards Assumption was violated

**Exposure**	Average aHR (95% CI)	Year 0	Year 1	Year 2	Year 3	Year 4	Year 5	Year 6	Year 7	Year 8	Year 9	Year 10	Year 11 (Jan to March)
Pre-existing COPD (vs. no COPD)	1.26 (1.22, 1.30)	1.19 (1.14-1.24) <0.0001	1.22 (1.18-1.26) <0.0001	1.25 (1.21-1.29) <0.0001	1.29 (1.24-1.33) <0.0001	1.32 (1.26-1.38) <0.0001	1.35 (1.28-1.43) <0.0001	1.39 (1.30-1.49) <0.0001	1.43 (1.32-1.55) <0.0001	1.47 (1.33-1.61) <0.0001	1.51 (1.35-1.68) <0.0001	1.55 (1.37-1.75) <0.0001	1.59 (1.38-1.82) <0.0001
Statistically significant: purple cells													

Age65+

Table E25: Yearly rate of arrhythmias among people Age65+ during follow-up, amongst people with COPD pre-diagnosis compared with no COPD, where the Proportional Hazards Assumption was violated

**Exposure**	**Average aHR (95% CI)**	**Year 0**	**Year 1**	**Year 2**	**Year 3**	**Year 4**	**Year 5**	**Year 6**	**Year 7**	**Year 8**	**Year 9**	**Year 10**	**Year 11 (Jan to March)**
Pre-existing COPD (vs. no COPD)	1.37 (1.27-1.48) <0.0001	1.57 (1.37-1.79) <0.0001	1.52 (1.36-1.70) <0.0001	1.47 (1.34-1.62) <0.0001	1.43 (1.31-1.55) <0.0001	1.38 (1.28-1.49) <0.0001	1.33 (1.24-1.45) <0.0001	1.30 (1.19-1.42) <0.0001	1.25 (1.13-1.39) <0.0001	1.21 (1.07-1.37) 0.002	1.18 (1.02-1.36) 0.027	1.14 (0.96-1.34) 0.124	1.01 (1.10-1.33) 0.310
Statistically significant: red cells													

QRISK>10%

Table E26: Yearly rate of MACE among people with QRISK>10% during follow-up, amongst people with COPD pre-diagnosis compared with no COPD, where the Proportional Hazards Assumption was violated

**Exposure**	Average aHR (95% CI)	Year 0	Year 1	Year 2	Year 3	Year 4	Year 5	Year 6	Year 7	Year 8	Year 9	Year 10	Year 11 (Jan to March)
**Pre-existing COPD (vs. no COPD)**		1.39 (1.34-1.44) <0.0001	1.37 (1.33-1.41) <0.0001	1.36 (1.32-1.39) <0.0001	1.34 (1.31-1.37) <0.0001	1.33 (1.30-1.35) <0.0001	1.31 (1.28-1.34) <0.0001	1.29 (1.26-1.33) <0.0001	1.28 (1.24-1.32) <0.0001	1.26 (1.22-1.31) <0.0001	1.25 (1.20-1.30) <0.0001	1.24 (1.18-1.29) <0.0001	1.22 (1.16-1.29) <0.0001
Statistically significant: grey cells													

Table E27: Yearly rate of MACE among people with HF history during follow-up, amongst people with COPD pre-diagnosis compared with no COPD, where the Proportional Hazards Assumption was violated

**Exposure**	Average aHR (95% CI)	Year 0	Year 1	Year 2	Year 3	Year 4	Year 5	Year 6	Year 7	Year 8	Year 9	Year 10	Year 11 (Jan to March)
**Pre-existing COPD (vs. no COPD)**		1.72 (1.59-1.87) <0.0001	1.68 (1.56-1.80) <0.0001	1.63 (1.53-1.73) <0.0001	1.59 (1.50-1.67) <0.0001	1.54 (1.47-1.62) <0.0001	1.50 (1.43-1.57) <0.0001	1.46 (1.39-1.54) <0.0001	1.42 (1.34-1.51) <0.0001	1.38 (1.29-1.48) <0.0001	1.34 (1.24-1.46) <0.0001	1.31 (1.19-1.43) <0.0001	1.27 (1.15-1.41) <0.0001
Statistically significant: grey cells													
